# Thermodynamic and Kinetic Characteristics of Molnupiravir Tautomers and Its Complexes with RNA Purine Bases as an Explanation of the Possible Mechanism of Action of This Novel Antiviral Medicine: A Quantum-Chemical Study

**DOI:** 10.1021/acs.joc.3c01580

**Published:** 2023-09-27

**Authors:** Wojciech Piotr Oziminski, Agata Bycul

**Affiliations:** Department of Organic and Physical Chemistry, Faculty of Pharmacy, Medical University of Warsaw, 1 Banacha Street, 02-097 Warsaw, Poland

## Abstract

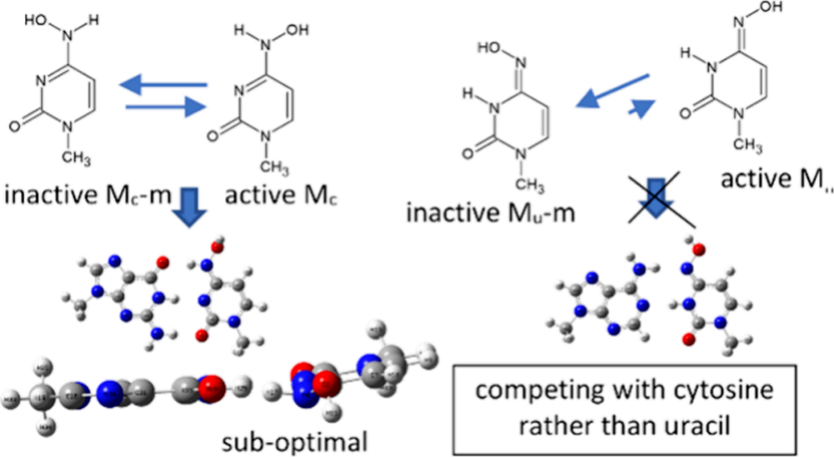

The mechanism of action of molnupiravir, a novel antiviral drug, was analyzed from the point of view of its tautomerism by means of quantum-mechanical calculations. It was established that although the uracil-like tautomer M_u_ (3 kcal/mol in the water environment) is the most thermodynamically stable, in fact, it is the cytosine-like tautomer M_c_ that plays the main role. There are several reasons, as follows: (1) A large part of M_u_ exists as a more stable but inactive form M_u_-m that is unable to pair with adenine. (2) The phosphorylated form of M_c_ is only 1 kcal/mol less stable than M_u_ in the water environment and thus is readily available for building into the RNA strand, where the M_u_/M_c_ energy gap increases and the probability of M_c_ → M_u_ interconversion leading to C → U mutation is high. (3) The guanine-M_c_ complex has similar stability to guanine-cytosine, but the adenine-M_u_ complex has lower stability than adenine-uracil. Additionally, the guanine-M_c_ complex has a suboptimal distorted geometry that further facilitates the mutations. (4) The activation barrier for proton transfer leading to M_u_-m interconversion into a cytosine-like tautomer is higher than for M_u_, which makes the uracil-like form even less available. These facts confirm an intriguing experimental observation that molnupiravir competes mainly with cytosine and not uracil.

## 1 Introduction

**Scheme 1:**
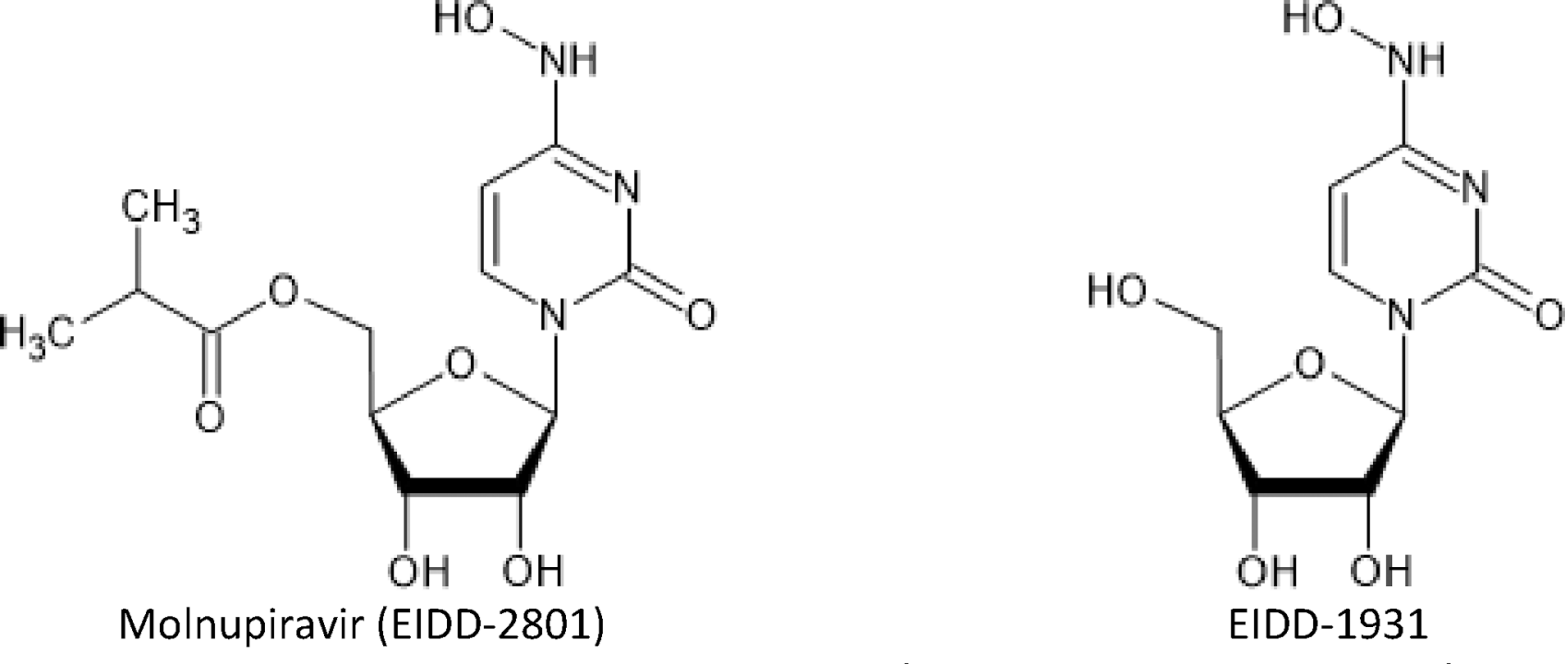
Schematic Representation of Molnupiravir and Its Active form

Viral disease treatment is difficult if the condition of the patient becomes serious and the natural immune system is unable to combat the infection. It was especially clear during the Covid-19 pandemic when several molecular targets for antiviral drugs were tried with various degrees of effectivity. In most cases, it was hard to definitely conclude if the drug was significantly helping the patient or not. Classical antiviral drugs like acyclovir and ribavirin were tried but rather without success.^1^ Next, chloroquine and hydroxychloroquine were used for treatment as they suppress the glycosylation of the ACE2 receptor, which should reduce the number of viruses entering the cell.^2^ Another promising and well-known medicine, amantadine, is an ion channel inhibitor and also showed some potential.^3^ Different molecular targets are virus enzymes. Nirmarelvir is a protease enzyme inhibitor used for Covid-19 treatment.^4^ Another interesting enzyme target is RNA-dependent RNA polymerase, RdRP, which is specific for viruses and does not exist in human cells. Thus, the RdRP inhibitors are potentially good and relatively safe antiviral drugs. For example, remdesivir was first developed for Ebola treatment and is an adenosine analogue.^5^ It was the first drug approved by the FDA for Covid-19 treatment.^6^*In vitro*^7^ and animal model^8^ studies showed its efficacy. Another drug from this class, molnupiravir, is a cytosine analogue and was studied already in 2013 at the Emory University in Atlanta for horse encephalitis treatment.^9^ It was tested first in animal models against Covid-19 and showed its efficacy.^10^ Recently, its efficacy also for Covid-19 treatment in humans was shown. *In vitro* studies showed its efficacy against Alpha, Beta, Gamma, Delta, and Omicron variants and the high sensitivity of the virus for the drug.^11^ It reduces the amount of secretion from the nasopharynx and has good bioavailability and good tolerance among patients.^12^ It reduces the time of infection by 50%.^13^ It was the first FDA- and MHRA-approved oral anti-Covid-19 medicine. Molnupiravir (EIDD-2801) is a prodrug and is depicted in Scheme 1 along with its postulated active form EIDD-1931.

**Scheme 2:**
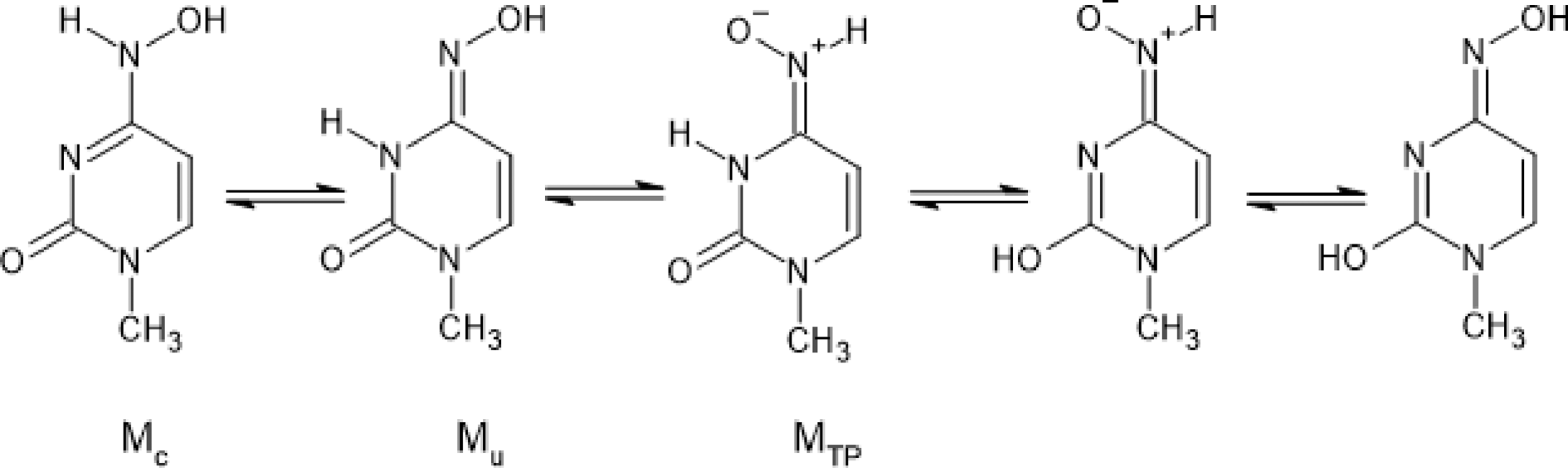
Possible Tautomers of Molnupiravir

Molnupiravir consists of an *N*-hydroxylated cytosine analogue connected via the ring nitrogen atom to the ribose acylated by isobutyric acid. Molnupiravir is hydrolyzed *in vivo* to EIDD-1931, which is subsequently phosphorylated to its active triphosphate form MTP.^14,15^ It is widely believed that molnupiravir exists as an analogue of cytosine, and because of this, it can compete with CTP in pairing with guanine and building into a viral RNA strand. However, the quantum-chemical calculations undoubtedly show that the uracil-like tautomeric form is more stable. Should it be thus pictured rather as a uracil-like tautomer? It turns out that the answer to this question is not simple, and we will try to shed some light on this topic. The tautomerism of molnupiravir is schematically depicted in Scheme 2 where the sugar part of the molecule was replaced by a methyl group for clarity.

In this study, we focus on two stable tautomers of molnupiravir that resemble cytosine and uracil; thus, they will be called M_c_ and M_u_. Only these two tautomeric forms can be paired with RNA bases—M_c_ with guanine and M_u_ with adenine—and thus can be incorporated into the virus genome and disturb its proliferation. Apart of these two, other tautomers have much higher energy and additionally are unable to be paired with RNA bases and thus will be not covered in this study. One exception is M_TP_, which is close in energy to M_c_ and is a transition product in one of the pathways of interconversion of molnupiravir tautomers and will be covered in the section about kinetics. There are some literature data about the energy difference between M_u_ and M_c_ tautomeric forms, but the question is not finally resolved. Jena calculated the monophosphate forms of these tautomers and claims that M_u_ (EIDD-2801) is more stable by 4.17 kcal/mol in the gas phase and only by 0.99 kcal/mol in water solution than M_c_ at the B3LYP-D3/6-311++G(d,p) level of theory.^16^ According to Sharov et al.,^17^ the energy gap is 6.84 kcal/mol in the gas phase at the B3LYP/6-311++G(d,p) level of theory for bare molnupiravir. However, both authors give the total electronic energy without thermochemical corrections, but a more proper quantity for stability comparisons would be the Gibbs free energy, which directly corresponds to the tautomeric equilibrium constant and the percentage of tautomers in the tautomeric mixture. Additionally, they have not analyzed in detail possible rotamers and geometric isomers of the tautomeric forms. Thus, further investigation and more reliable calculations are needed. In general, a possibility of the existence of a pyramidic base as two tautomers introduces the possibility of RNA mutations.^18^ The smaller the energy gap between the two tautomers of molnupiravir is, the larger is its potential for mutagenesis. Gordon et al.^19^ and Kabinger et al.^20^ investigated the competition of molnupiravir triphosphate (MTP) with natural nucleosides uridine triphosphate (UTP) and cytidine triphosphate (CTP), and they determined that MTP competes most effectively with CTP. MTP also competes with UTP, but according to these authors, this is less effective. This fact is intriguing and should be explained because keeping in mind that M_u_ is more stable, molnupiravir should be present mainly as a uracil-like tautomer and should compete with uracil rather than cytosine. If we assume that M_c_ is paired with guanine (G) and then, after being built into the RNA strain, tautomerizes to M_u,_ it can then be paired in the next cycle of replication with adenine (A), resulting in G → A mutation. Subsequently, the A will pair with uracil (U); thus, we can also speak of C → U mutations. If these mutations are frequent enough, they will push the replication over the “error threshold” that is too large for replication fidelity; thus, the activity of the virus will diminish.^21^ So, the important questions of this study are the following: (1) What is the Gibbs free energy difference between the M_c_ and M_u_ tautomers? Is it small enough to enable this kind of mutations? (2) How do ribose and phosphate change the tautomeric equilibrium of molnupiravir? (3) Why does molnupiravir seem to compete with cytosine rather than uracil? (4) How does the energy of the complexes of molnupiravir with adenine and guanine compare to natural complexes AU and GC? It was also postulated by Gordon et al.^19^ and Kabinger et al.^20^ that the hydrogen bonds in M_u_-A and M_c_-G are “suboptimal”, and this assumption will also be checked by optimizing the appropriate complexes.

## 2 Results and Discussion

### 2.1 Tautomerism of Molnupiravir

**Figure 1:**
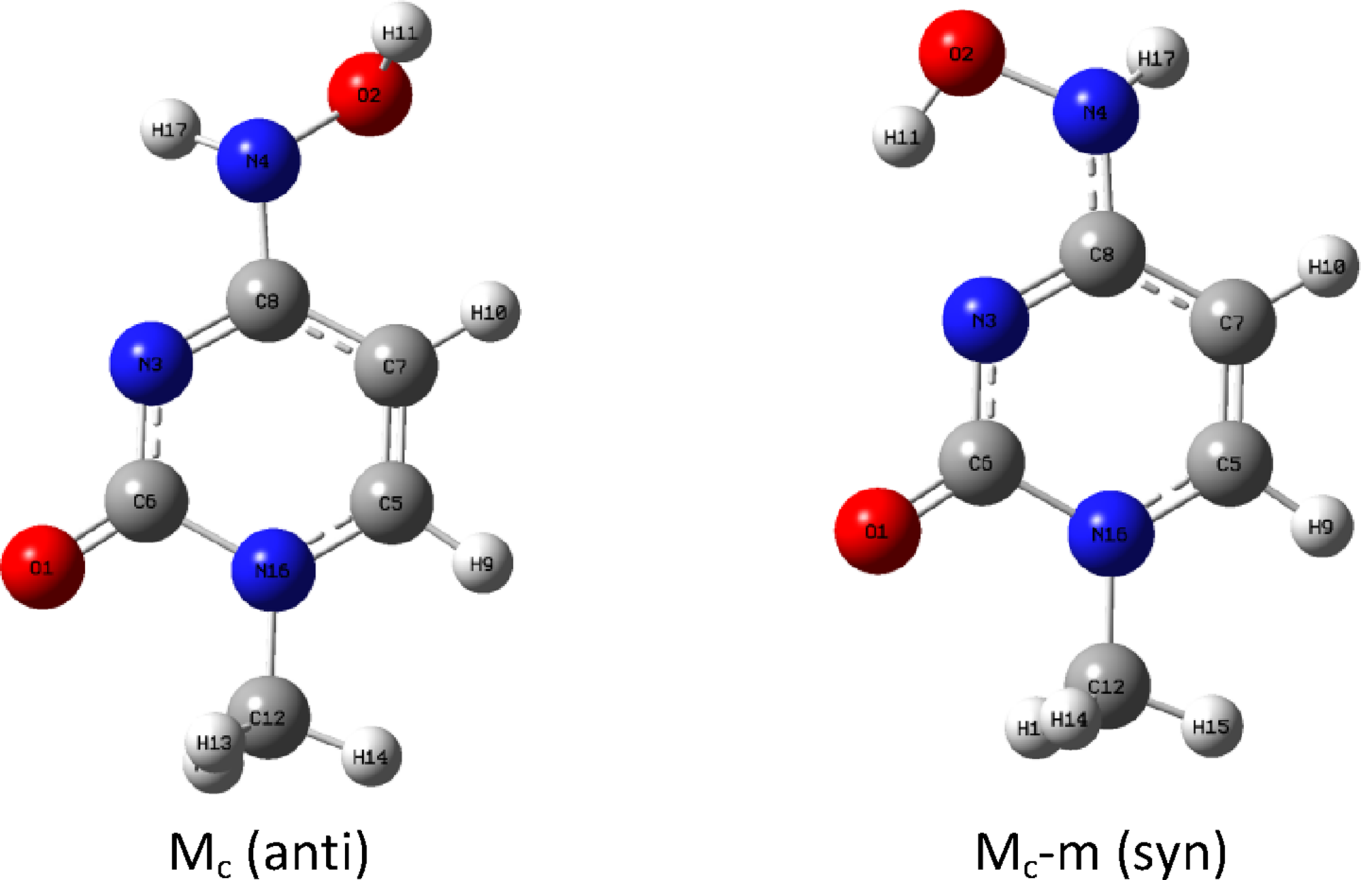
Two possible rotamers of amino tautomer M_c_: (a) M_c_ (anti) and (b) M_c_-m (syn).

First, the acylated ribose was substituted by a methyl group to form the simplest model of molnupiravir. However, the influence of externally connected ribose and phosphate will be analyzed later. This simple scheme of only two tautomeric forms is complicated by the fact that in the amino tautomer M_c_, we have two rotatable single bonds, i.e., C–N and N–O, and because of these, there are two possible conformations of the OH group. Thus, it can exist as an anti rotamer in the shape proper for forming a complex with guanine but also as a second, more stable syn rotamer with an intramolecular hydrogen bond but unable to pair with guanine. We will call the first “proper” rotamer M_c_ (anti) and the second M_c_-m (syn) where “m” stands for “minimum energy” (Figure 1).

**Figure 2:**
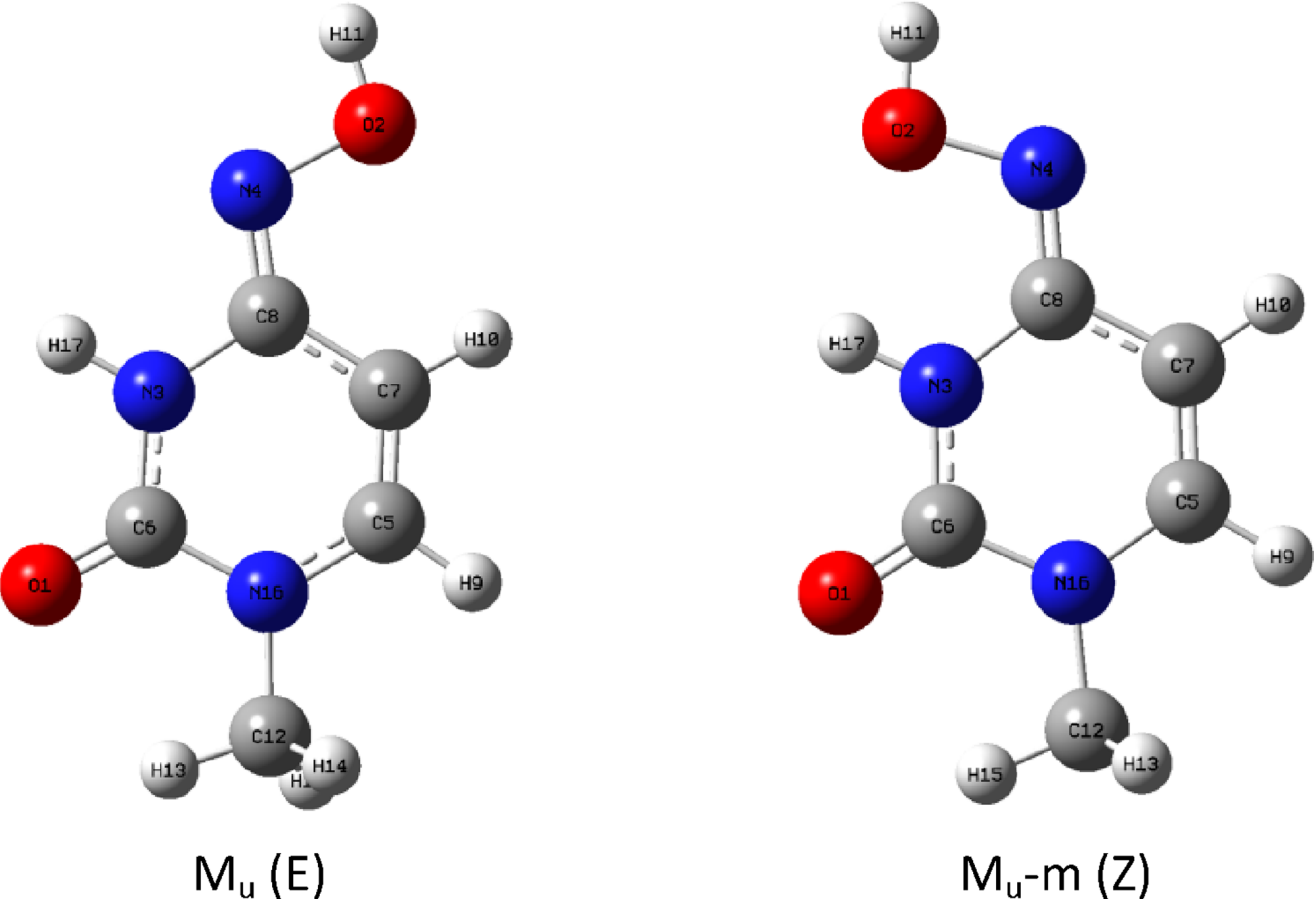
Two possible rotamers of imino tautomer M_u_: (a) M_u_ (E) and (b) M_u_-m (Z).

A similar situation exists in the case of the imino tautomer M_u_ where, because of the double C=N bond, we have two geometric isomers: Z and E. The E-isomer has proper orientation to form a complex with adenine, but the second Z-isomer has lower energy. We will call the first “proper” isomer M_u_ (E) and the second M_u_-m (Z) (Figure 2).

In this case, again, M_u_-m is unable to mimic uracil and pair with adenine. This is maybe less clear than in the case of M_c_ because it may seem that the oxygen atom from the OH group could make a hydrogen bond with adenine. However, calculations show that the AM_u_-m complex is heavily distorted and much less stable than the normal AM_u_ complex, which will be covered in more detail in the chapter about RNA complexes. One important thing to notice is that in the case of M_c_/M_c_-m, the interconversion between rotamers is easy, but in the case of M_u_/M_u_-m, the C=N bond is double, and there is no direct interconversion between E and Z isomers.

In Table 1, the energetic data for the two tautomers and its rotamers optimized using orbital basis sets of increasing quality in the gas phase and the modeled via PCM model are shown. The positive values mean that the first isomer is less stable. The last column shows the results of CCSD(T) single point energy calculations that yield very accurate energetic results.

**Table 1:** Gas Phase Relative Gibbs Free Energies of Molnupiravir and Its Isomers in kcal/mol

	6-31G(d)	6-311++G(d,p)^a^	aug-cc-pVDZ	aug-cc-pVTZ	CCSD(T)^b^
M_c_/M_c_-m	0.62	0.76 (0.64)	0.89	1.11	0.93
M_u_/M_u_-m	2.81	2.58 (2.22)	2.52	2.44	2.85
M_c_-m/M_u_-m	9.52	9.02 (9.27)	8.00	8.17	9.17
M_c_/M_u_	6.47	7.20 (7.70)	6.37	6.84	7.26

a In parentheses are given values optimized at the B3LYP-D3/6-311++G(d,p) level of theory.

b CCSD(T)/aug-cc-pVTZ//B3LYP/aug-cc-pVTZ single point energy.

To check if the inclusion of dispersion correction has a significant impact on the results, we performed B3LYP-D3/6-311++G(d,p) optimizations on all molnupiravir isomers. It follows from Tables 1 and 2 that, the effect of dispersion is very small in the case of the M_c_/M_u_ tautomer pair, and the difference is zero in the water environment and only 0.5 kcal/mol in gas phase. The only significant difference can be seen for M_u_/M_u_-m geometric isomers: it is almost 1 kcal/mol (PCM). We also checked the CCSD(T)/6-311++G(d,p) single point energy calculations for M_u_/M_c_ on both B3LYP/6-311++G(d,p) and B3LYP-D3/6-311++G(d,p) geometries, and the results are very similar: 7.21 (CCSD(T)//B3LYP-D3) vs 6.73 kcal/mol (CCSD(T)/B3LYP) in the gas phase and the same value of 2.62 kcal/mol in the water environment. Therefore, in all calculations, the standard B3LYP functional was employed. It turns out that the energy difference between M_c_ and M_c_-m rotamers is very small, especially in a water environment. It is well below the calculation error, so we can assume that both rotamers have similar stability and exist in similar amounts. The probable reason for this is that in M_c_-m, the strong hydrogen bond stabilization is compensated for by unfavorable steric interaction between N–H and C–H on the other side of the molecule. A different situation exists for M_u_ where the difference in Gibb free energy between two geometric isomers M_u_-m (Z) and M_u_ (E) is about 2 kcal/mol in favor of the M_u_-m isomer in the gas phase and in the water environment. This indicates that the M_u_-m isomer should dominate in the mixture. The possible reason for the lower energy of M_u_-m is that the weak intramolecular hydrogen bond interaction in this isomer is stronger than in M_u_. The fact that the more stable uracil-like form M_u_-m is unable to make a pair with adenine is important, as it means that the majority of the uracil-like tautomer of molnupiravir cannot be incorporated into the RNA strand and remains inactive. This may be one of the reasons why it was observed experimentally that molnupiravir competes with cytosine rather than with uracil. Comparing the two tautomers M_c_ and M_u_, it follows that the M_u_ is more stable, especially in the gas phase. If we compare the minimum-energy isomers M_c_-m and M_u_-m, the same remains true, but the energy gap is larger by about 2 kcal/mol. A closer look at the details of tautomeric interconversion reveals an interesting fact. Only M_u_ can directly tautomerize to M_c_ via a single proton transfer. The M_u_-m geometric isomer is unable to tautomerize via a single proton transfer. However, it could tautomerize to M_c_-m via some kind of transition product with a probably higher activation barrier. This will be investigated in detail in the chapter about kinetics. Comparing the results obtained by orbital basis sets of increasing accuracy, it follows from Tables 1 and 2 that the results show clear trends especially when comparing M_u_/M_u_-m or M_c_/M_c_-m. This is because these molecules are so similar; there is the same type of bonding, and they are only different in geometric isomers. When comparing the energy difference of different tautomers, namely, M_c_/M_u_ or M_c_-m/M_u_-m, the trends are not always clear. Sometimes, energy diminishes, and sometimes, it rises. This is because tautomers are different molecules with partially different bonding patterns. However, we should trust mostly the results obtained with the largest basis set, aug-cc-pVTZ, and especially the single point energy CCSD(T) values. The comparison of the relative stability in the gas phase and the water environment is interesting. We see that for each tautomer of molnupiravir, its isomers have similar relative stability (M_c_/M_c_-m and M_u_/M_u_-m) when going from the gas phase to the water environment. However, when we compare the tautomers (M_c_/M_u_ and M_c_-m/M_u_-m), we see that the differences are large. M_u_ is more stable in the gas phase than M_c_ by 7.26 kcal/mol, but in the water environment, this is reduced to only 3.06 kcal/mol. What is the reason for this? To understand this behavior, we should take a look at dipole moments that are gathered in Table 3.

**Table 2:** Water Environment (PCM) Relative Gibbs Free Energies of Molnupiravir and Its Isomers in kcal/mol^a^

	6-31G(d)	6-311++G(d,p)^b^	aug-cc-pVDZ	aug-cc-pVTZ	CCSD(T)^c^
M_c_/M_c_-m	0.12	0.01 (−0.09)	0.13	0.28	0.30
M_u_/M_u_-m	2.47	2.43 (1.55)	2.39	2.33	2.76
M_c_-m/M_u_-m	4.91	5.07 (4.30)	4.42	4.51	5.52
M_c_/M_u_	2.57	2.65 (2.65)	2.16	2.46	3.06

a Absolute Gibbs free energies are available as Supporting Information (Tables S1 and S2).

b In parentheses are given values optimized at the B3LYP-D3/6-311++G(d,p) level of theory.

c CCSD(T)/aug-cc-pVTZ//B3LYP/aug-cc-pVTZ single point energy.

**Table 3:** Dipole Moments of Molnupiravir Isomers Calculated at the B3LYP/6-311++G(d,p) Level

	dipole moment
M_u_	3.12
M_u_-min	3.02
M_c_	5.60
M_c_-min	6.32

The differences in dipole moments between rotamers or geometric isomers are very small, but the differences between the tautomers are large. Thus, the energy of M_c_ and M_c_-min will be lowered in the water environment by the solvation effect more than M_u_ and M_u_-min, which explains the trends.

### 2.2 Tautomerism of Cytosine and Uracil

Cytosine and uracil can also exist as alternative tautomers, and the same methodology and model chemistry were used for the estimation of their relative energy. The situation is however simpler as cytosine and uracil and their tautomers are in their lowest energy conformations. Only two high-energy rotamers are possible, which we can safely ignore in our analysis.

**Figure 3:**
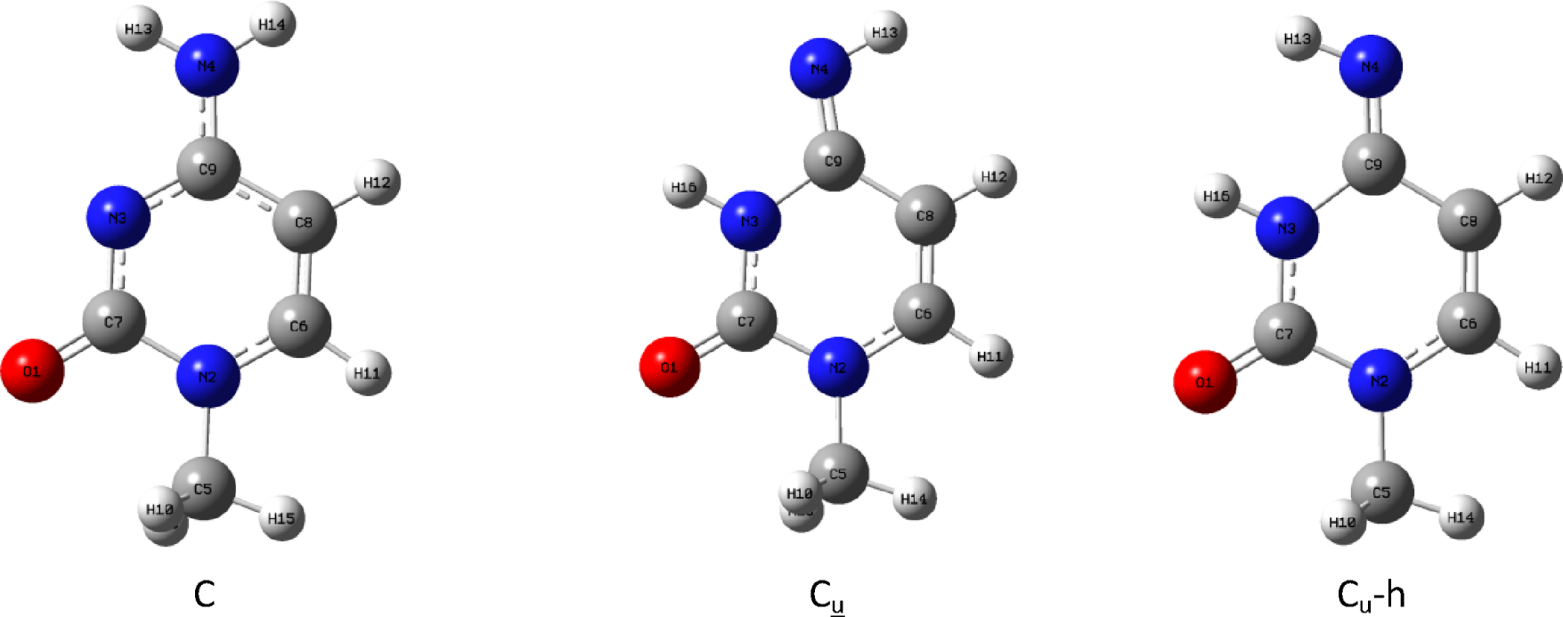
Main tautomer of cytosine: C, its alternative tautomer: C_u_ and the high-energy rotamer: C_u_-h

**Figure 4:**
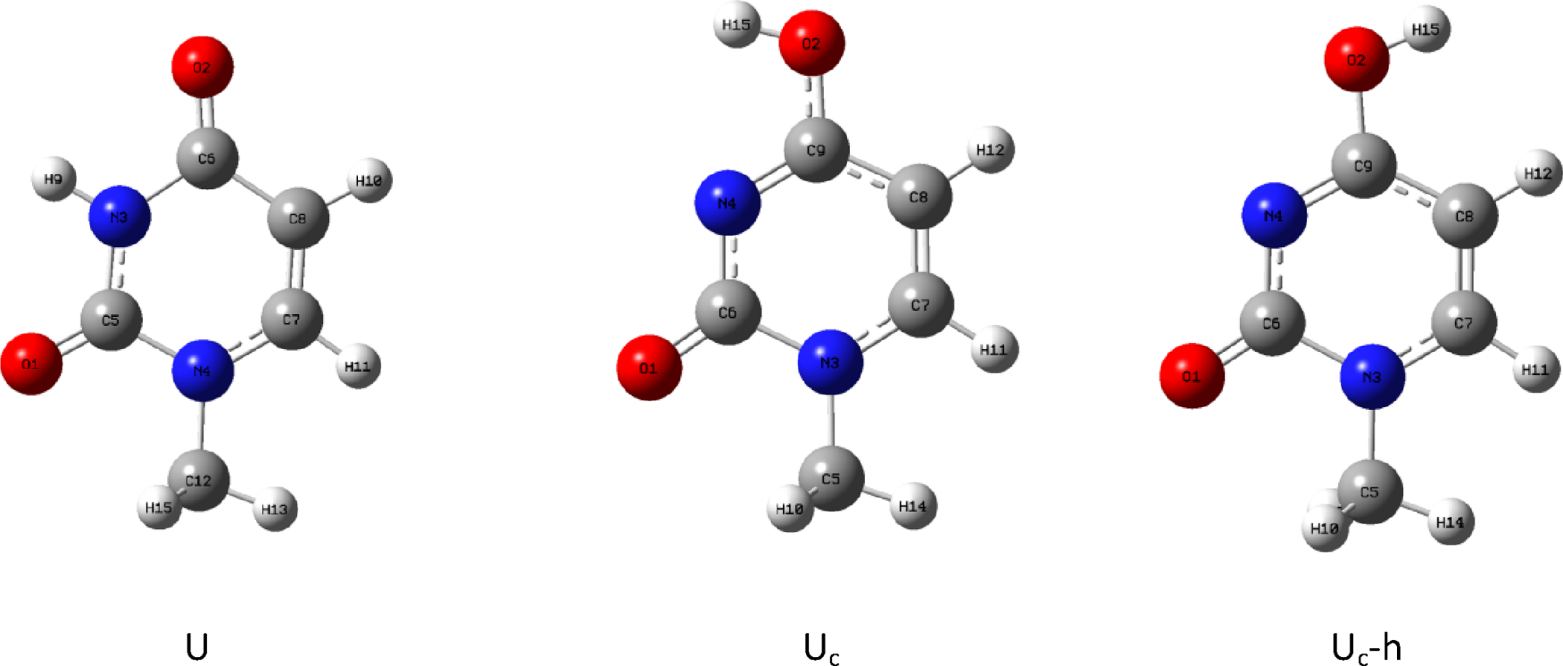
Main tautomer of uracil: U, its alternative tautomer: U_c_, and the high-energy rotamer: U_c_-h.

As cytosine is more symmetric than molnupiravir, there is only one possible rotamer for cytosine. In the case of its tautomer C_u_, there is a possible rotamer that has the configuration other than needed for the creation of complex with G, but it is less stable; therefore, we will call it C_u_-h (“h” for high energy) (Figure 3). In the case of uracil, we have again one rotamer for uracil but two possible rotamers for U_c_ tautomer. The rotamer with conformation not suitable for the creation of complex with adenine has again higher energy and therefore will be called U_c_-h (Figure 4). All energetic data are gathered in Tables 4 and 5.

**Table 4:** Gas Phase Relative Gibbs Free Energies of Cytosine and Uracil and Their Isomers in kcal/mol

	6-31G(d)	6-311++G(d,p)	aug-cc-pVDZ	aug-cc-pVTZ	CCSD(T)^a^
C	0.00	0.00	0.00	0.00	0.00
C_u_	2.09	1.84	3.13	2.94	**1.83**
C_u_-h	3.65	3.79	4.60	4.50	3.28
U	0.00	0.00	0.00	0.00	0.00
U_c_	12.93	12.07	10.75	11.10	**10.43**
U_c_-h	19.73	18.63	16.65	16.98	16.34

a CCSD(T)/aug-cc-pVTZ//B3LYP/aug-cc-pVTZ single point energy.

**Table 5:** Water Environment (PCM) Relative Gibbs Free Energies of Cytosine and Uracil and Their Isomers in kcal/mol^a^

	6-31G(d)	6-311++G(d,p)	aug-cc-pVDZ	aug-cc-pVTZ	CCSD(T)^b^
C	0.00	0.00	0.00	0.00	0.00
C_u_	6.15	7.18	7.08	7.07	**6.08**
C_u_-h	7.29	8.26	8.13	8.19	7.15
U	0.00	0.00	0.00	0.00	0.00
U_c_	11.84	11.05	10.14	10.45	**9.41**
U_c_-h	14.63	13.40	12.29	12.54	11.54

a Absolute Gibbs free energies are available as Supporting Information (Tables S3 and S4).

b CCSD(T)/aug-cc-pVTZ//B3LYP/aug-cc-pVTZ single point energy.

It follows from Tables 4 and 5 that the C_u_ tautomer is less stable by 1.83 kcal/mol in the gas phase, but this is increased to 6.08 kcal/mol in the water environment. Thus, we can assume that this tautomer is present in negligible amounts in the tautomeric mixture and does not compete effectively with uracil in complex creation with adenine. The alternative rotamer C_u_-h is even less stable in both environments, and we will not consider it in the discussion. The case of uracil is similar, but the energy gap between tautomers is larger, i.e., 10.43 kcal/mol in the gas phase and 9.41 kcal/mol in the water environment. The alternative U_c_-h rotamer is also less stable and unable to create a complex with guanine. The large difference in Gibbs free energy between the gas phase and the water environment for cytosine tautomers and the small difference for uracil can again be simply explained by simple comparison of dipole moments, which are gathered in Table 6

**Table 6:** Dipole Moments of Cytosine and Uracil Isomers Calculated at the B3LYP/6-311++G(d,p) Level

	dipole moment (gp)
C	6.15
C_u_	4.89
U	4.94
U_c_	4.73

Similarly to the case of molnupiravir, cytosine has a larger dipole moment than its C_u_ tautomer; thus, its stability in the water environment will increase. In the case of uracil, its dipole moment is similar to that of U_c_.

### 2.3 A Comparison of the Structure, Electronic Properties, and Aromaticity of Molnupiravir, Cytosine, and Uracil

**Figure 5:**
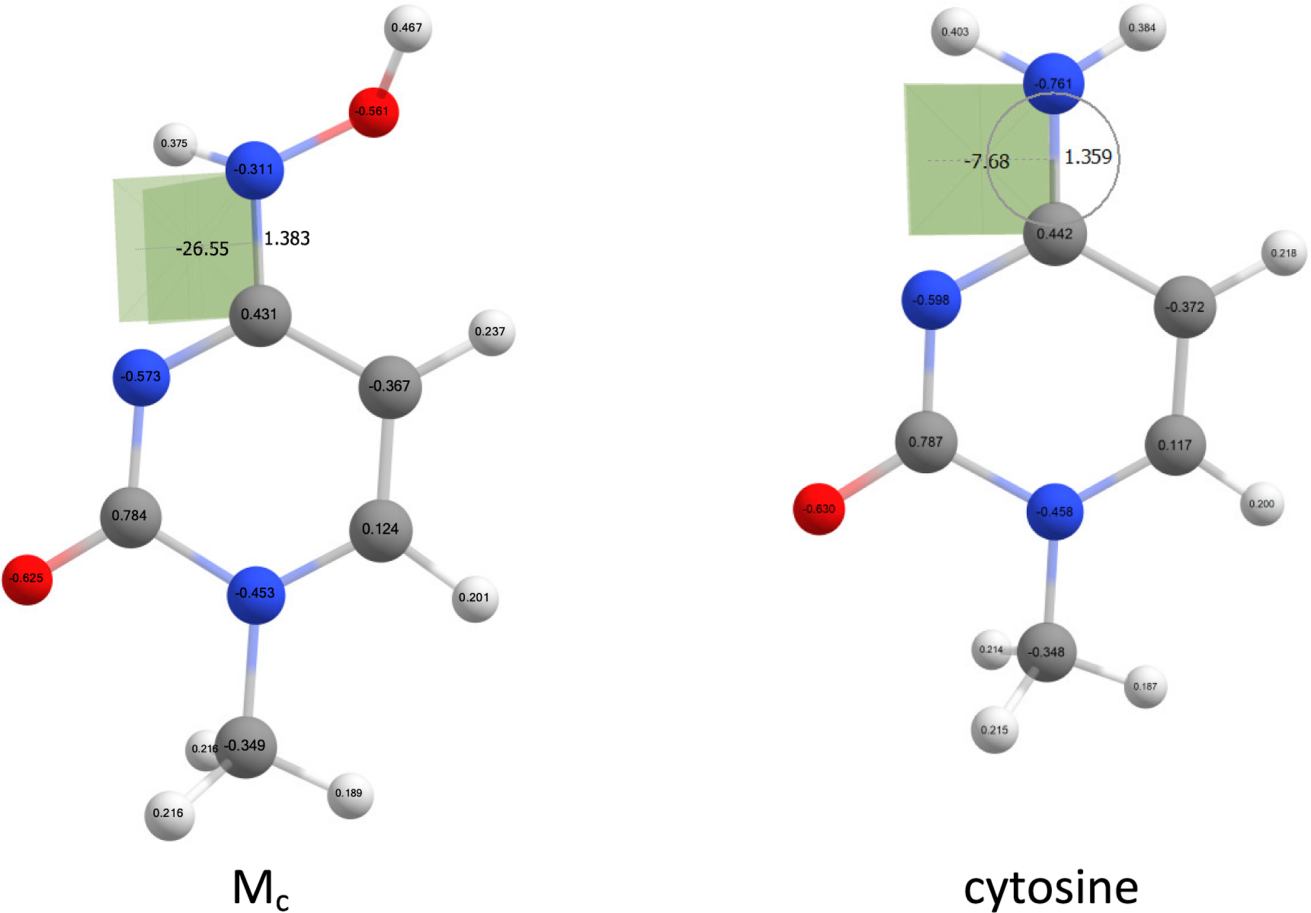
NBO charges and selected bond length and dihedral angle for M_c_ and cytosine obtained at the B3LYP/6-311++G(d,p) level of theory.

It follows that the substitution of one of the hydrogen atoms in the amino group of cytosine by an OH group has a deep impact on the behavior of molnupiravir: the M_u_ tautomer becomes more stable than M_c_. However, the energy difference between tautomers is small enough for the existence of both forms in water conditions. Thus, molnupiravir can compete either with cytosine or with uracil in complex creation with purine bases. This is in striking contrast to cytosine and uracil where the alternative tautomers are much less stable especially in the water environments and the probability of building into RNA the improper tautomer is very small. From the structural point of view, the impact of hydroxy group substitution to cytosine is also large. In Figure 5, structures of M_c_ and cytosine are compared.

**Figure 6:**
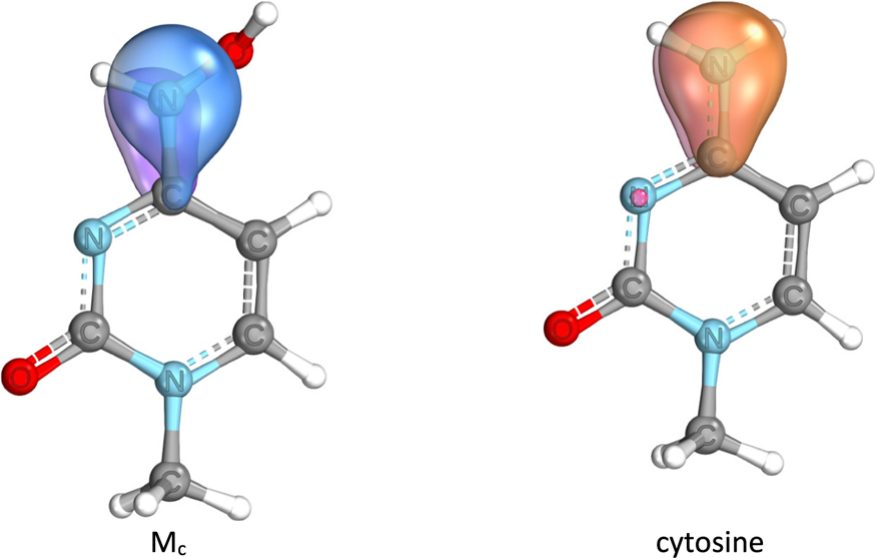
IBO localized orbital of nitrogen atom in molnupiravir and cytosine.

It follows that the NHOH group in M_c_ is much more pyramidal than the NH_2_ group in cytosine; the NCNH dihedral angle is only 7.68° for cytosine but increases up to almost 27° in M_c_. This may be caused by the electron-withdrawing properties of the hydroxyl group. The NBO charge on amino nitrogen atom is −0.761e in cytosine but only −0.311e in M_c_. Weaker conjugation in M_c_ can be also seen from the lengthening of the C–N bond from 1.359 Å in cytosine to 1.383 Å in M_c_. Another perspective is to look at the amino nitrogen lone pair in the IBO (intrinsic bond orbital) picture (Figure 6).

It follows from Figure 6 that the free lone electron pair in molnupiravir is effectively attracted to the oxygen atom, and thus, its delocalization to the ring is weaker. To understand better these structural and electronic features, the aromaticity of studied molecules was investigated using geometric HOMA, electronic pEDA, and magnetic NICS indices.

**Figure 7:**
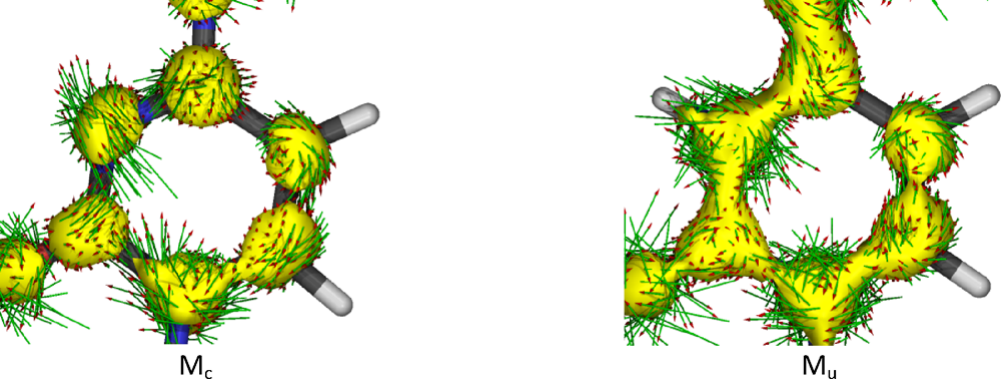
ACID maps for M_c_ and M_u_ calculated at the B3LYP/6-311++G(d,p) level of theory.

The pEDA index shows that all studied molecules are pi excessive, and M_u_ and its rotamer possess the largest pi excess. These molecules involve formally two pyrrole-like nitrogen atoms with their free lone electron pairs contributing to the ring pi density, and additionally, the external OH group is positioned in the plane of the ring; thus, it can also act as a pi donor to the ring. This pi excess causes the lowering of aromaticity that is reflected in both NICS(1)_ZZ_ and HOMA. On the contrary, the M_c_ tautomer has smaller pi excess and thus higher aromaticity, being the most aromatic compound in Table 7 according to NICS(1)_ZZ_ and HOMA. The external OH group is not in the ring plane in M_c_ and cannot act as a pi donor but only as a sigma acceptor. Interestingly, in the M_c_-m rotamer, the OH group lies in the ring plane, and indeed, the pi excess of M_c_-m is larger than that of M_c_. Lower values of pEDA for M_c_ than cytosine confirm the electron-withdrawing properties of the hydroxy group picture already in Figure 6. To confirm that low values of NICS(1)_ZZ_ for M_c_ really reflect its aromaticity, ACID (anisotropy of current induced density) maps were calculated for M_c_ and M_u_ and are shown in Figure 7.

**Table 7:** Geometric, Electronic, and Magnetic Aromaticity Indices Calculated at the B3LYP/6-311++G(d,p) Level of Theory for Molnupiravir, Cytosine, and Uracil and Their Alternative Tautomers

	HOMA	pEDA	NICS(1)_*zz*_
M_c_	0.740	0.637	–7.08
M_c_-m	0.726	0.736	–5.47
M_u_	0.607	1.156	1.86
M_u_-m	0.612	1.162	1.37
C	0.706	0.683	–6.12
C_u_	0.569	1.101	0.56
U	0.570	0.959	–2.68
U_c_	0.738	0.625	–7.92

It follows from Figure 7 that there is more delocalized pi-electron density in M_u_ (higher pi excess), but there is no consistent ring current. On the contrary, in M_c_, we see the clockwise ring current symbolized by the arrows that shows that the negative NICS(1)_ZZ_ value of M_c_ indeed shows its moderately high aromaticity.

### 2.4 The Influence of Ribose and Phosphate Addition on Molnupiravir Tautomerism

**Figure 8:**
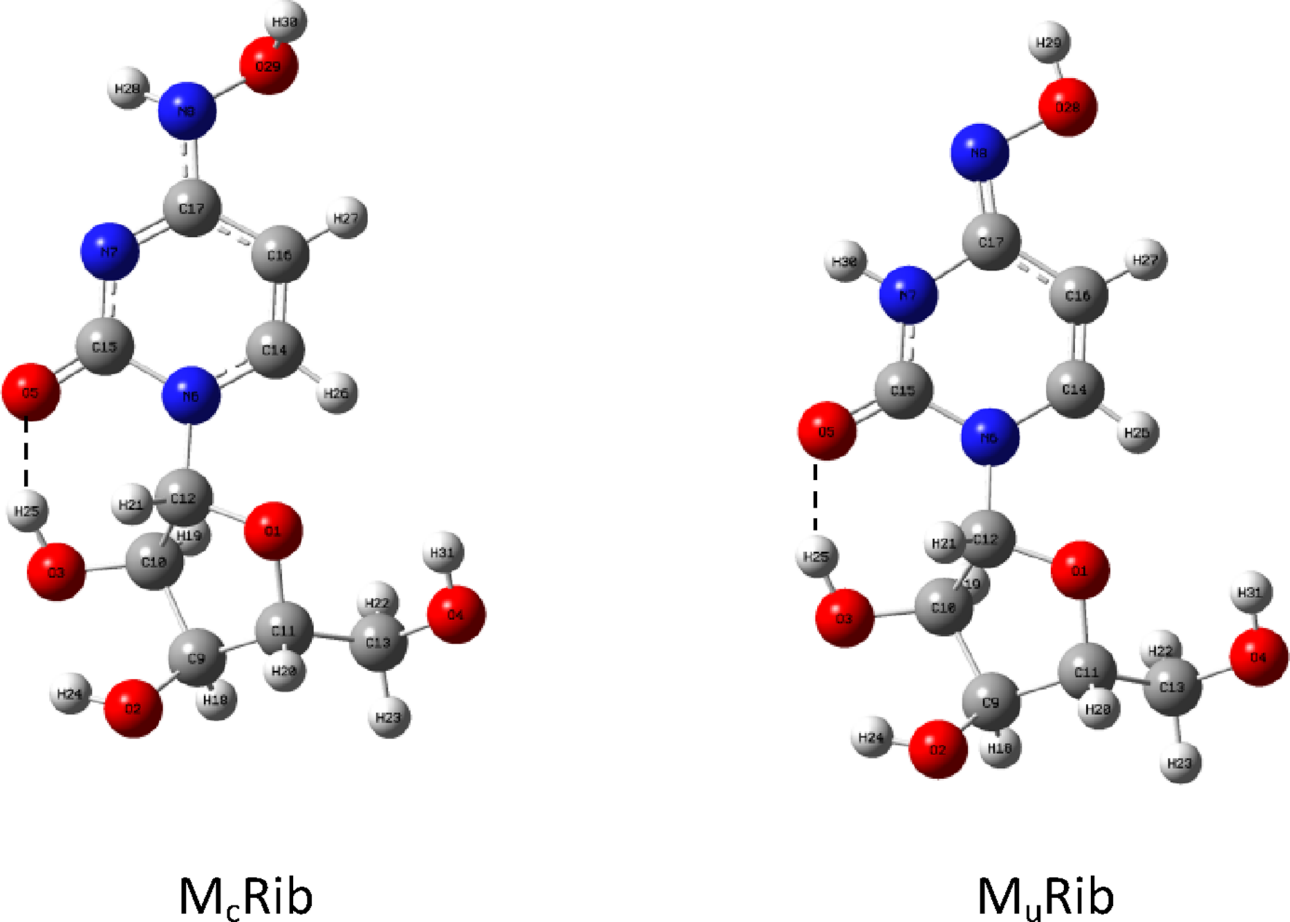
Molnupiravir tautomers with ribose.

To check the influence of a molecule of ribose on tautomer energy difference, a model of ribose adduct to M_c_ and M_u_ was built (M_c_Rib and M_u_Rib), and the lowest energy conformers were found and optimized at the B3LYP/6-311++G(d,p) level of theory (Figure 8).

The difference in Gibbs free energy between tautomers is lowered from 7.2 to 4.3 kcal/mol for the gas phase and from 2.6 to only 1.2 kcal/mol for the water environment. It follows that the energy gap decrease is substantial. This model could be used to estimate the tautomer energy difference when built into the RNA strand; the problem is, however, that the spatial conformation of the molnupiravir-ribose adduct in RNA differs from that of the free molecule. In fact, only in the free adduct can an intramolecular hydrogen bond between the nucleobase oxygen atom and ribose OH group (O5···O3H25 in Figure 8) be formed. The formation of this bond (dashed line in Figure 8) may strongly influence the energy gap between tautomers. Therefore, it is worth to check the energy difference with the absence of this bond. For this purpose, a model was built where the −O3H25 hydroxy group was replaced by a hydrogen atom. They will be called M_c_dRib and M_u_dRib, and their structures are available in the Supporting Information. In this case, the M_c_dRib/M_u_dRib energy gap for the gas phase is 6.5 kcal/mol, and that in the water environment is 2.9 kcal/mol; therefore, we see that these values are close to molnupiravir without ribose. It follows from looking at the tautomers' energy difference when built into the RNA strand that we should rather take the M_c_dRib/M_u_dRib or M_c_/M_u_ values; thus, the M_u_ tautomer is still definitely more stable than M_c_. However, regarding free molecules in the solution, we see that the energy gap becomes small; thus, a substantial part of molnupiravir exists as M_c_ tautomer. The influence of ribose addition on M_c_/M_c_-m and M_u_/M_u_-m energy gaps was also checked and is almost negligible.

**Figure 9:**
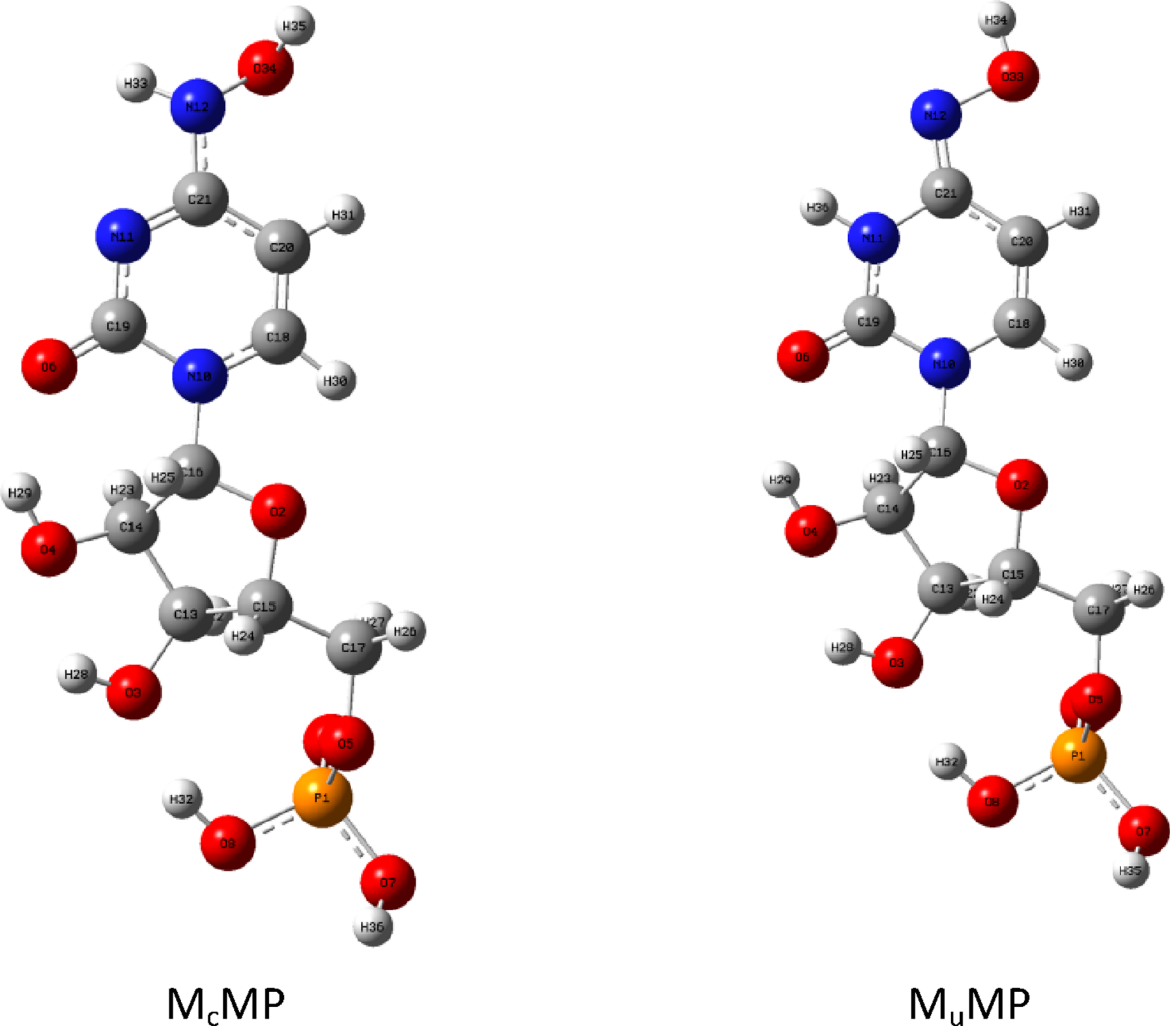
Molnupiravir tautomers with ribose and phosphate.

Before the insertion of molnupiravir into the RNA strand, it exists in the water environment *in vivo* in the form of triphosphate. Therefore, the influence of the phosphate group will also be examined. For this purpose, the MMP (monupiravir monophosphate) model will be adequate as the addition of two more phosphate groups should have negligible results on monupiravir tautomerism. The monophosphate analogues of M_c_ and M_u_ are shown in Figure 9. Again, the conformational analysis was performed, and the most stable conformers were reoptimized at the B3LYP/6-311++G(d,p) level of theory. The Gibbs free energy difference between the M_c_MP and M_u_MP is 3.99 kcal/mol for the gas phase and only 0.96 kcal/mol in the water environment. Thus, we observe even further slight lowering of the energy gap compared to the molnupiravir-ribose adduct. The MMP models are shown in the figure below.

Values of the M_c_/M_u_ Gibbs free energy gap depending on the moiety bound to nitrogen atom are summarized in the table below.

It follows from Table 8 that the lowering of the M_c_/M_u_ energy gap is almost monotonic. The only exception is a slight increase from methyl to deoxyribose in the water environment. The addition of the phosphate group has a small effect compared to ribose addition, but it was expected. It follows that we can draw the conclusion that the energy difference between uracil-like and cytosine-like tautomers of molnupiravir monophosphate (and thus triphosphate) is small enough (0.96 kcal/mol) to enable the existence of substantial amounts of M_c_MP available for building into the RNA strand. When molnupiravir is built into the RNA, the energy gap between tautomers substantially increases. The M_u_ tautomer becomes much more stable (2.94 kcal/mol), and thus, there is a strong tendency for M_c_ → M_u_ tautomerization that results in G → A and C → U mutations. This would be a second explanation to the fact that Gordon et al.^19^ and Kabinger et al.^20^ determined that MTP competes most effectively with CTP.

**Table 8:** Gibbs Free Energy Difference between M_c_ and M_u_ Tautomers Calculated at the B3LYP/6-311++G(d,p) Level of Theory for Various Moieties Connected to the Nitrogen Atom

moiety connected to the nitrogen atom	gas phase [kcal/mol]	water environment (PCM) [kcal/mol]
M_c_-M_u_	7.20	2.65
M_c_dRib-M_u_dRib	6.53	2.94
M_c_Rib-M_u_Rib	4.27	1.15
M_c_MP-M_u_MP	3.99	0.96

### 2.5 The Kinetics of Proton Transfer in Molnupiravir

**Figure 10:**
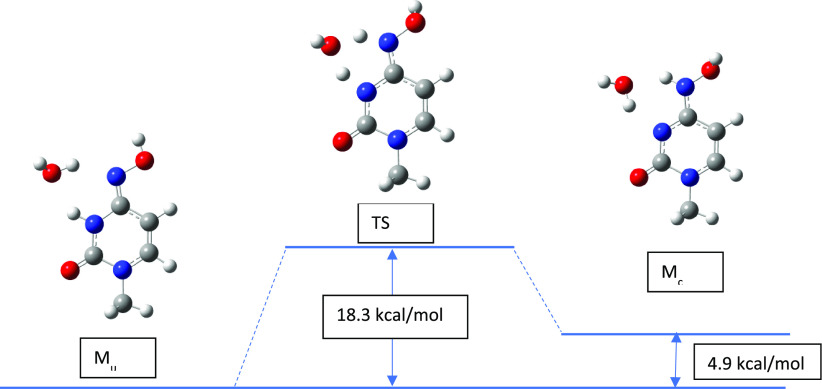
Reactant, transition state, and product for the M_u_ → M_c_ tautomerization process with a single water helper molecule optimized at B3LYP/6-311++G(d,p) level of theory.

To calculate the activation barrier for proton transfer in molnupiravir, two alternative pathways were analyzed. First, for the M_u_ → M_c_ interconversion, a simple one-step mechanism was proposed where a single water molecule is employed to facilitate the proton transfer because, without it, the activation barrier would be very high. Tautomerization occurs most often in water solution, so there are many water molecules available. The reactants and transition states are depicted in Figure 10.

**Figure 11:**
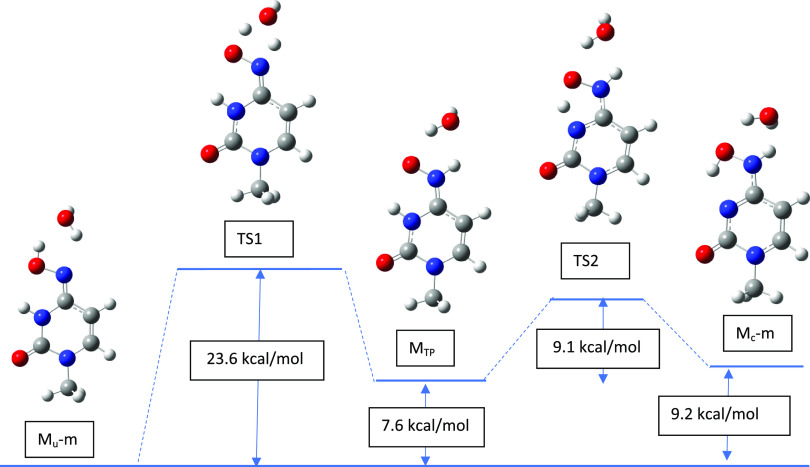
Reactant, transition state, and product for the M_u_-m → M_c_-m tautomerization process with a single water helper molecule optimized at B3LYP/6-311++G(d,p) level of theory.

The structure of the transition state is typical, with two protons lying between the water oxygen and two nitrogen atoms. A similar picture in the water environment is available in the Supporting Information (Figure S1) where the two protons are closer to nitrogen atoms. The activation Gibbs free energy is 18.3 kcal/mol in the gas phase and 16.6 kcal/mol in the water environment, which shows that proton transfer occurs easily. For the process of interconversion of M_u_-m → M_c_-m, a different pathway is proposed with teh inclusion of a transition product M_TP_ (see Figure 11). In this case, the first step also involves a water molecule as a helper and leads to a transition product via an activation barrier of 23.6 kcal/mol in the gas phase and 21.6 kcal/mol in the water environment. Thus, the interconversion process is much slower. Next, the transition product M_TP_ can easily interconvert to the final tautomer M_c_-m with an activation barrier of only about 9–10 kcal/mol.

The reverse process M_c_-m → M_u_-m would have a smaller activation barrier for the slowest step; it would be 16.09 kcal/mol in the gas phase and 19.59 kcal/mol in the water environment (Supporting Information, Figure S2). This may also contribute to the lower availability of active M_u_ as there is a kinetic tendency for some kind of accumulation of M_u_-m because of the higher activation barrier. Also, this seems to be in line with the concept of molnupiravir competing mainly with cytosine. The transition product M_TP_ is quite interesting as its energy (Figure 11) is lower than M_c_-m. However, we performed additional B3LYP/aug-cc-pVTZ calculations (without a water molecule), and they show that M_TP_ is less stable by 1.4 kcal/mol in the gas phase and more stable by 0.11 kcal/mol in the water environment than M_c_-m. This molecule and its stability are interesting by themselves; however, we will not analyze it further here as it cannot form complexes with guanine just as M_u_-m or M_c_-m. The activation energy data are summarized in Table 9 where cytosine and uracil are also shown for comparison.

**Table 9:** Enthalpy of Activation and Gibbs Free Energy of Activation of the Highest Energy Transition State Calculated in the Gas Phase and the Water Environment Relative to the Appropriate Reactant

	gas phase		water environment (PCM)	
transition state	Δ*H*^#^ [kcal/mol]	Δ*G*^#^[kcal/mol]	Δ*H*^#^ [kcal/mol]	Δ*G*^#^[kcal/mol]
M_u_ → M_c_	15.4	18.3	14.0	16.6
M_u_-m → M_c_-m	21.6	23.6	18.8	21.6
C → C_u_	13.6	15.8	13.3	15.8
U → U_c_	15.0	17.6	14.6	17.0

It follows that the activation barrier for M_u_ → M_c_ proton transfer is comparable to those of cytosine and uracil and that the barrier for M_u_-m → M_c_-m is substantially higher. As expected in all cases, the Gibbs free energy is higher than enthalpy as the entropy contribution is negative.

### 2.6 Complexes with RNA Bases

**Figure 12:**
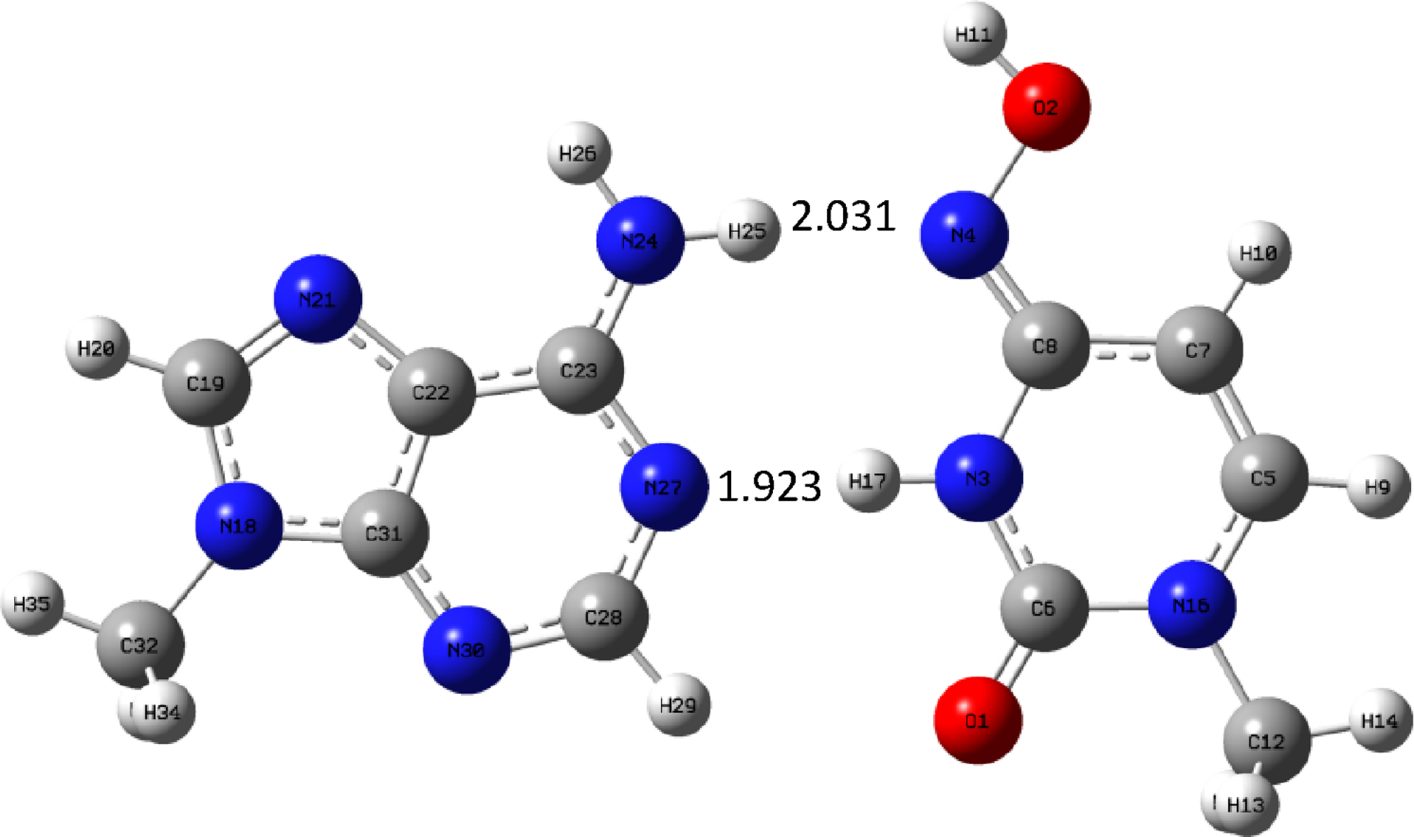
AM_u_ complex optimized at the B3LYP/aug-cc-pVTZ (PCM) level.

**Figure 13:**
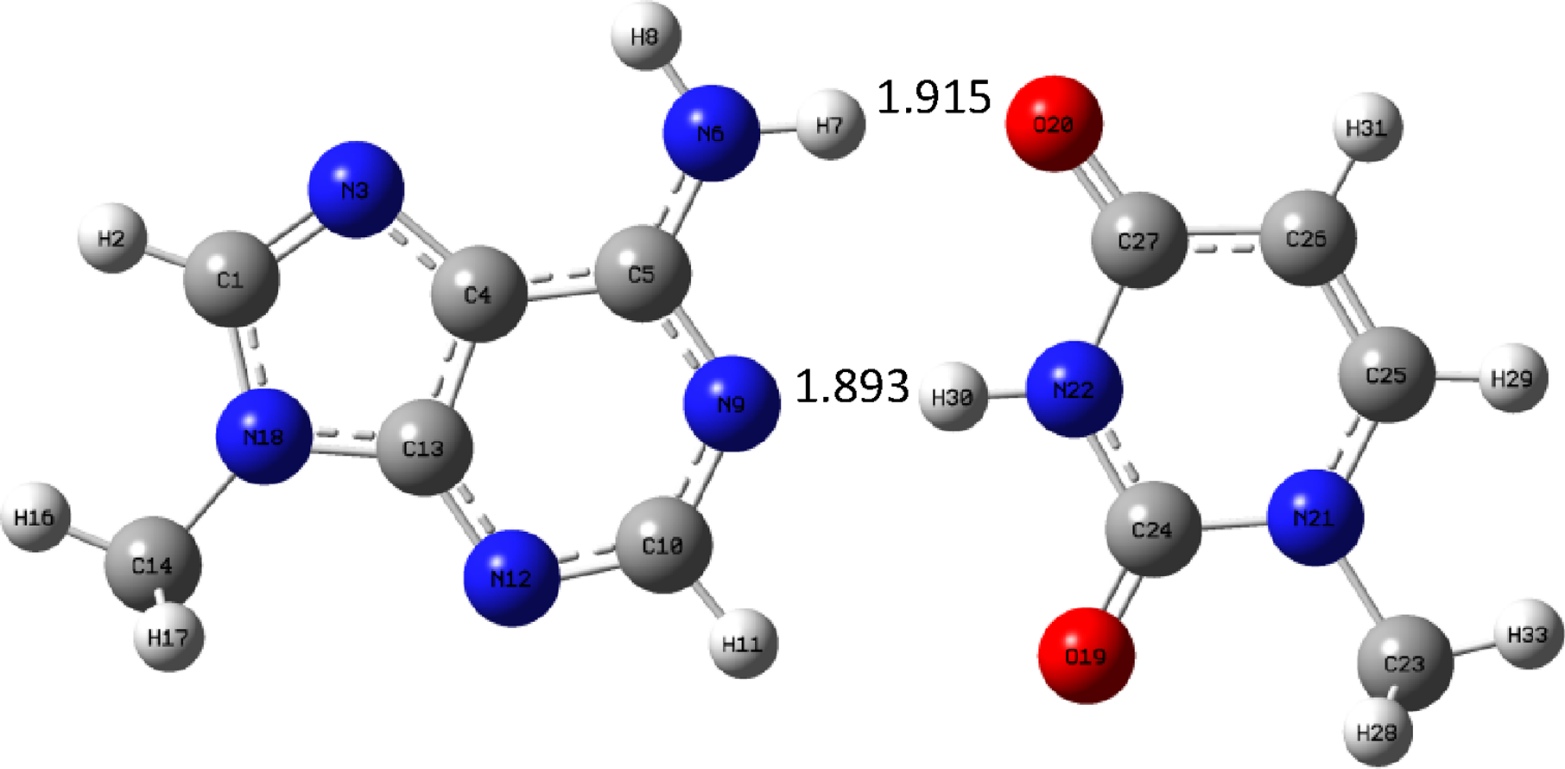
Complex AU optimized at the B3LYP/aug-cc-pVTZ (PCM) level.

**Figure 14:**
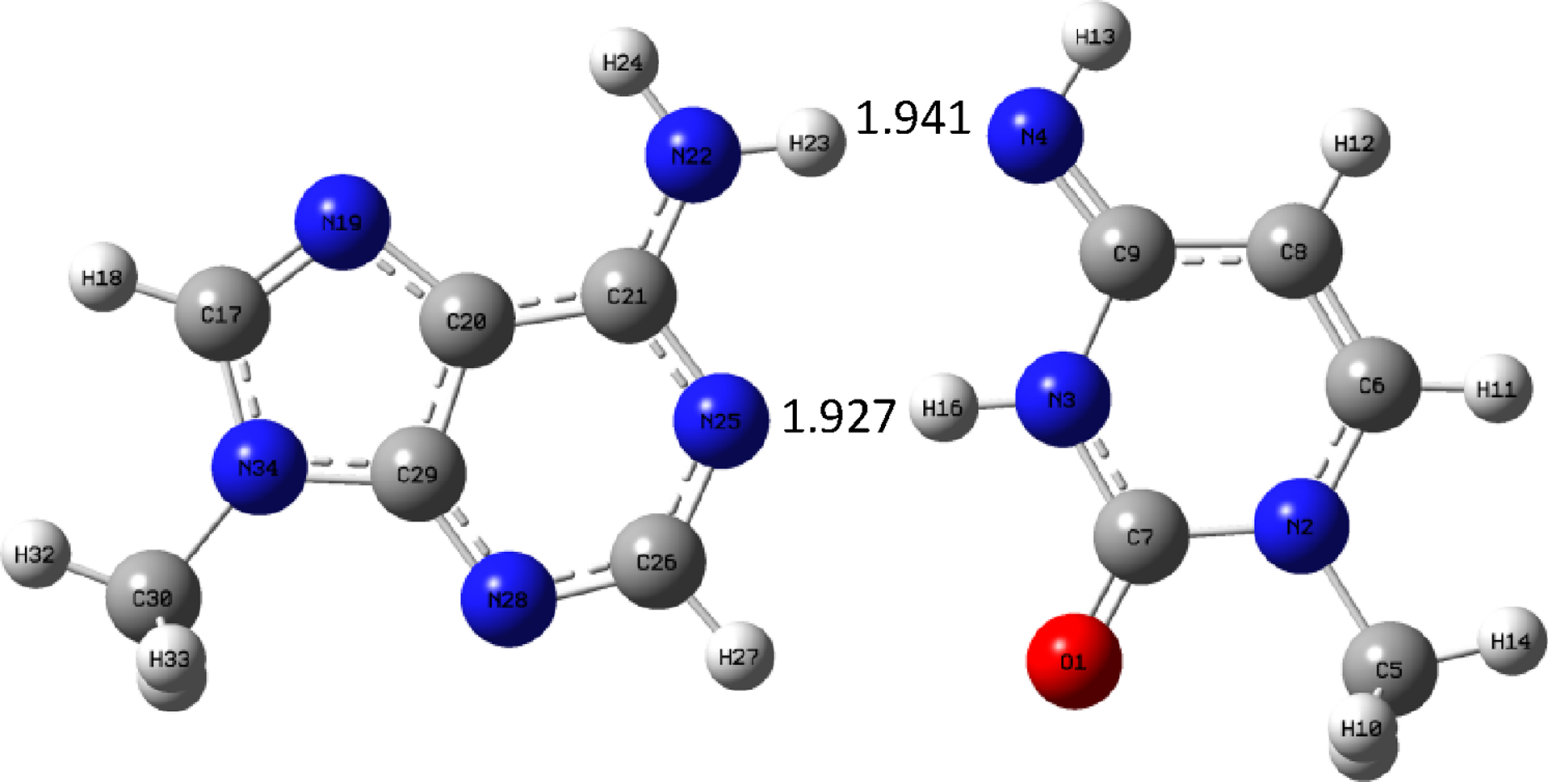
Complex AC_u_ optimized at the B3LYP/aug-cc-pVTZ (PCM) level.

To estimate the binding energy of purine-pyrimidine base pairs, adenine and guanine were optimized at the appropriate theoretical levels. For consistency, the pyrrole-like nitrogen atom of the five-membered ring was methylated in all cases. In this section, we give the enthalpy of formation of the complex because it reflects best the binding energy of the complex. Relative Gibbs free energies along with all absolute values of the enthalpy and Gibbs free energy are given in the Supporting Information in Tables S9–S14. The next step was building the complexes of molnupiravir and its tautomers with appropriate RNA bases because it is interesting to see how stable the complexes of molnupiravir with RNA bases are and also to analyze the hydrogen bond lengths in these complexes. Therefore, M_u_ was paired with adenine and M_c_ with guanine. Also, naturally occurring complexes AU and GC were built to compare them to complexes with molnupiravir. To make this picture more complete, the alternative tautomers of naturally occurring bases were paired with appropriate puric bases: GU_c_ and AC_u_. Complexes with adenine are shown in Figures 12–14. First, the complex AM_u_ is given, then the natural complex AU is shown, and finally, AC_u_ is shown.

From the structural point of view, the complex AM_u_ is most similar to AC_u_ as there are similar hydrogen bonding patterns, where the nitrogen atom is both a hydrogen bond donor and an acceptor. On the contrary, in the natural AU complex, the hydrogen bond donor is a nitrogen atom, but the role of acceptor is played by both nitrogen and oxygen atoms. It is interesting to compare the geometry of these complexes, particularly the hydrogen bond lengths. There are two hydrogen bonds. The first is formed by a hydrogen atom from the NH_2_ adenine group and an oxygen or nitrogen atom of the second base. In the case of uracil, the bond is formed with oxygen atom and is the shortest, i.e., −1.915 Å. When we go to C_u_, the bond elongates, and for M_u_, it is the longest and therefore the weakest. The reason is that the OH group connected to imino-nitrogen atom N4 is a sigma-electron-withdrawing group; therefore, it reduces the electron density on the lone pair of this nitrogen atom and weakens the hydrogen bond. The second hydrogen bond is formed similarly by the hydrogen atom from the N–H group of pyrimidine base and the nitrogen atom from purine. A similar trend can be observed: the bond is the shortest for AU and longer for AC_u_, but for AM_u_, it is of very similar length (a little bit shorter). It follows from the bond lengths that the expected binding energy order is AU > AC_u_ > AM_u._ The energetics of adenine complexes are shown in Table 10 for the gas phase and in Table 11 for the water environment.

**Table 10:** Gas Phase Enthalpy of Complex Formation from Base Pairs in the Conformation of Pyrimidine Bases “as in Complex”^a^

	6-31G(d)	6-311++G(d,p)	aug-cc-pVDZ	aug-cc-pVTZ
AM_u_	–13.06	–9.70	–9.95	–8.94 (−6.11)
AU	–15.00	–11.44	–11.83	–10.78
AC_u_	–15.28	–11.72	–11.99	–10.84
GM_c_	–27.24	–23.46	–23.74	–22.66 (−21.37)
GC	–28.14	–23.90	–24.19	–22.99
GU_c_	–29.59	–25.38	–25.91	–24.79

a Relative values are given in kcal/mol. Values in parentheses compare to the lowest energy isomer of molnupiravir. Absolute enthalpies and Gibbs free energies of purine bases are available in the Supporting Information (Tables S5–S8).

**Table 11:** Water Environment (PCM) Enthalpy of Complex Formation from Base Pairs in the Conformation of Pyrimidine Bases “as in Complex”^a^

	6-31G(d)	6-311++G(d,p)	aug-cc-pVDZ	aug-cc-pVTZ
AM_u_	–10.13	–6.20	–6.48	–4.94 (−2.47)
AU	–10.82	–6.53	–6.94	–5.42
AC_u_	–11.49	–7.09	–7.49	–5.84
GM_c_	–17.53	–11.97	–12.40	–11.36 (−10.97)
GC	–17.77	–11.83	–12.33	–11.20
GU_c_	–20.21	–14.65	–15.30	–14.20

a Relative values are given in kcal/mol. Values in parentheses compare to the lowest energy isomer of molnupiravir. Relative Gibbs free energies of the complexes are available in the Supporting Information (Tables S9 and S10). Absolute enthalpies and Gibbs free energies of the complexes are available as in the Supporting Information (Tables S1–S14).

Our convention is that we subtract the enthalpies of free bases from the enthalpy complex, so the negative enthalpy means that the enthalpy of the complex is lower than substrates. Let us first analyze the basis set effect. This is a well-known fact that in the case of calculating the energy of a complex, a basis set superposition error occurs, which is most pronounced for small basis sets. This effect leads to the overestimation of binding energy of the complex, and there are two ways to deal with it: one is the counterpoise correction method, and the second is to use a sufficiently extended basis set. In this paper, the second method was applied, and to see the effect in detail, we present the results for increasing basis sets. It follows from Tables 10 and 11 that, for each complex, the binding energy is less negative as the basis set size increases. The effect is particularly large with 6-31G(d) and 6-311++G(d,p). For the smaller basis set, the binding energy is overestimated by several kcal/mol. Moving from 6 to 311++G(d,p) to Dunning aug-cc-pVDZ shows that these two bases are of similar quality. Next, the aug-cc-pVTZ basis set gives the least negative values, which is expected. In all following analyses, we will concentrate only on the most reliable aug-cc-pVTZ results. It follows from Table 10 that, according to the enthalpy, all complexes are stable. However, according to Gibbs free energy, only complexes with guanine in the gas phase are stable thermodynamically (Supporting Information, Tables S9 and S10). This is quite obvious as the entropy of the complex formation is highly negative and only very strong guanine complexes containing three hydrogen bonds have negative G and can exist as free molecules. The complex of AM_u_ is less stable than AU and AC_u_ complexes, which is in agreement with the concept of Gordon et al.^19^ and Kabinger et al.^20^ that it is “suboptimal”. Especially if we compare the enthalpy of the complex to the minimum conformer of M_u_ (value in parentheses), this is clearly seen. Regarding the complex GM_c_, the binding of the complex is only slightly weaker even considering the minimum conformer of M_c_; nevertheless, the effect is present.

Including the water environment lowers the binding enthalpy of all complexes, but the trends remain. It is interesting that according to Jena,^16^ the complexes of molnupiravir are much stronger, i.e., −11.22 kcal/mol for AM_u_ (EIDD-2810:A) and −17.89 kcal/mol for GM_c_ (EIDD-1931:G), despite a similar theoretical level. This author does not precisely state which kind of energy he used; we assume that it was pure electronic energy without any thermochemical corrections. The geometries, especially hydrogen bond lengths, obtained by this author are also significantly different.

An additional possibility is the formation of the M_u_-m isomer with adenine, which was also explored. It follows that the geometry of the optimized complex is highly nonplanar (the structure is included in the Supporting Information) and the enthalpy of the complex formation is only −4.44 kcal/mol in the gas phase and −1.32 kcal/mol in the water environment at the B3LYP/aug-cc-pVTZ level. Therefore, the probability of the formation of such base pair in the RNA strand is rather low.

**Figure 15:**
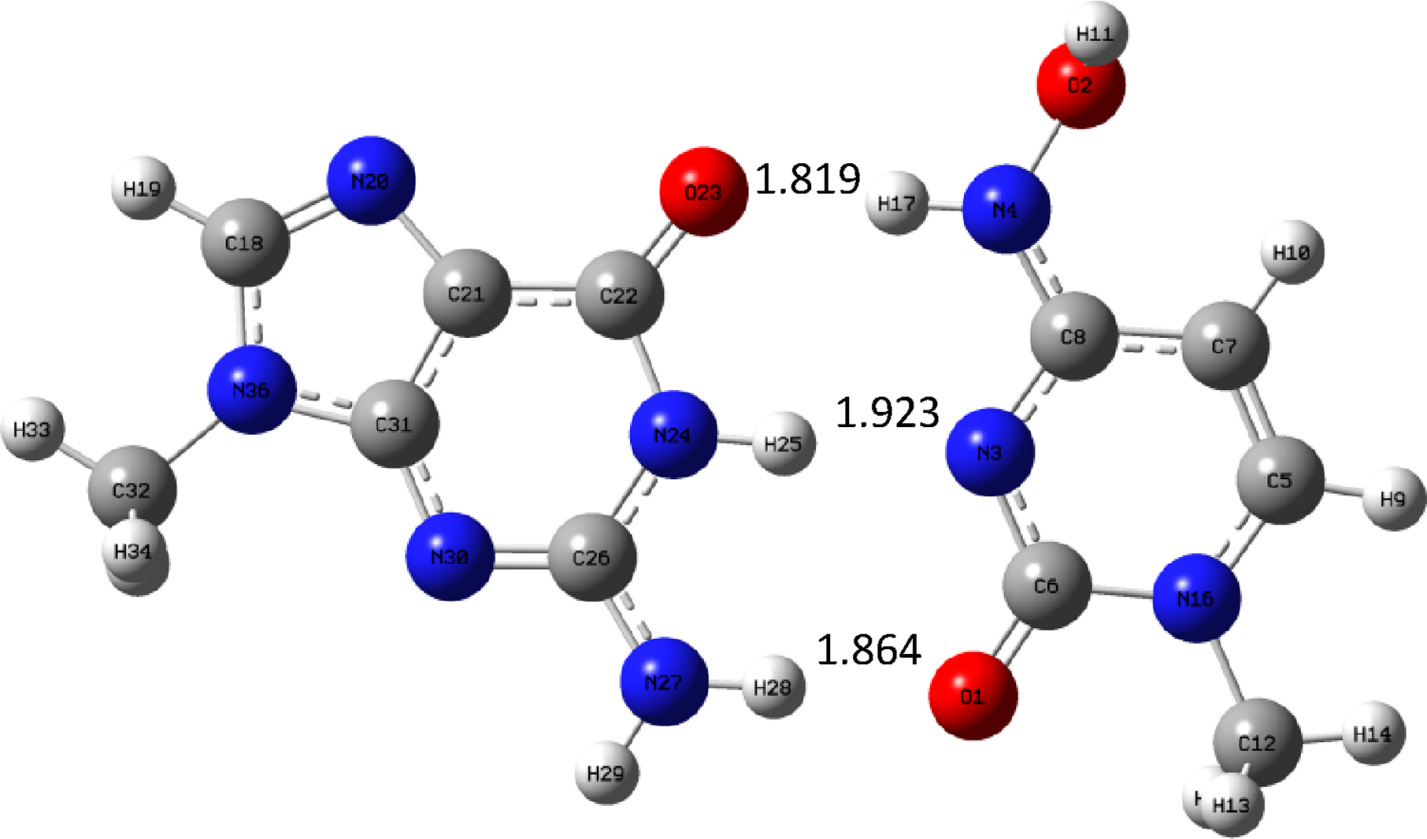
Complex GM_c_ optimized at the B3LYP/aug-cc-pVTZ (PCM) level.

**Figure 16:**
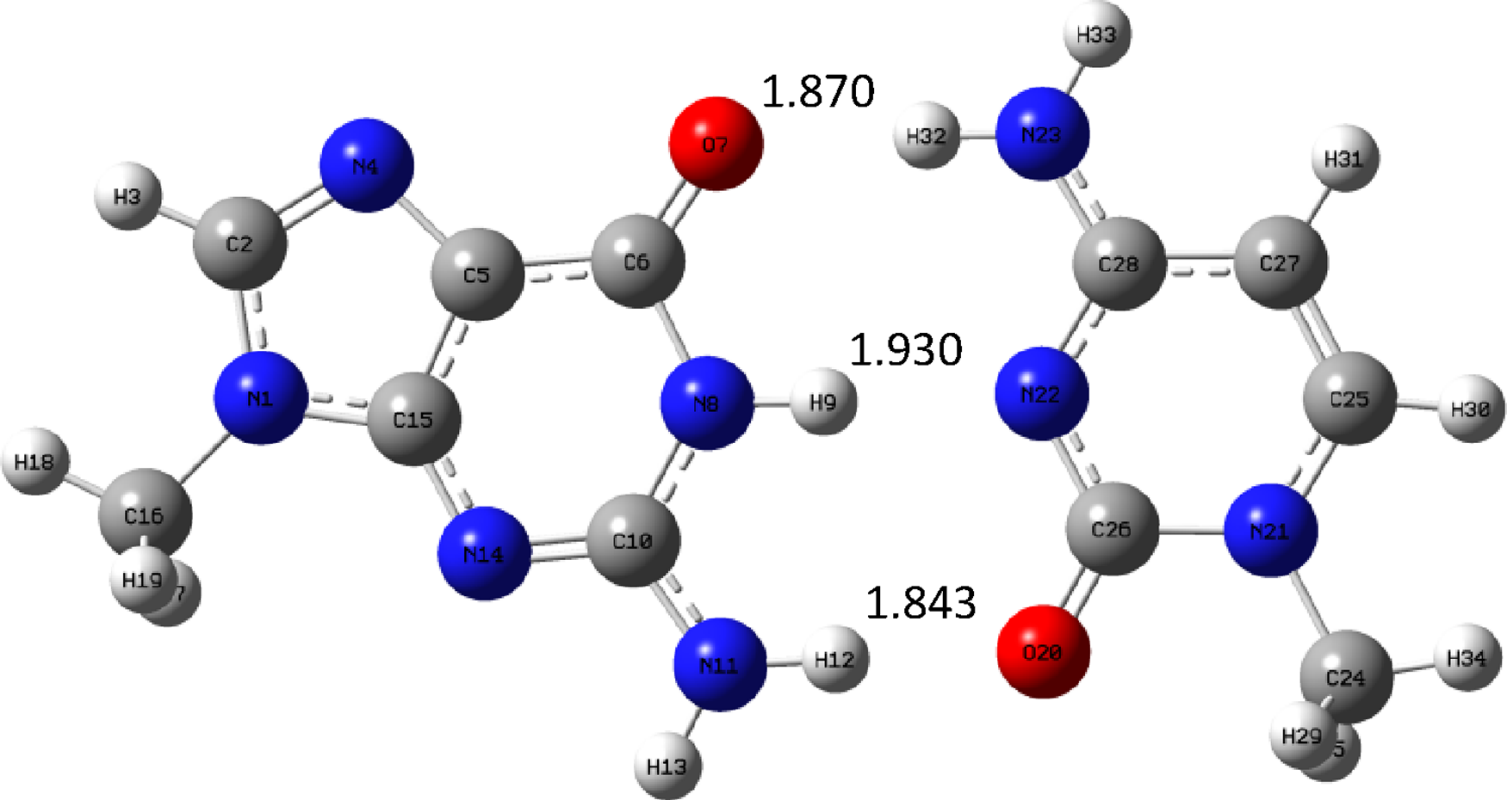
Complex GC optimized at the B3LYP/aug-cc-pVTZ (PCM) level.

**Figure 17:**
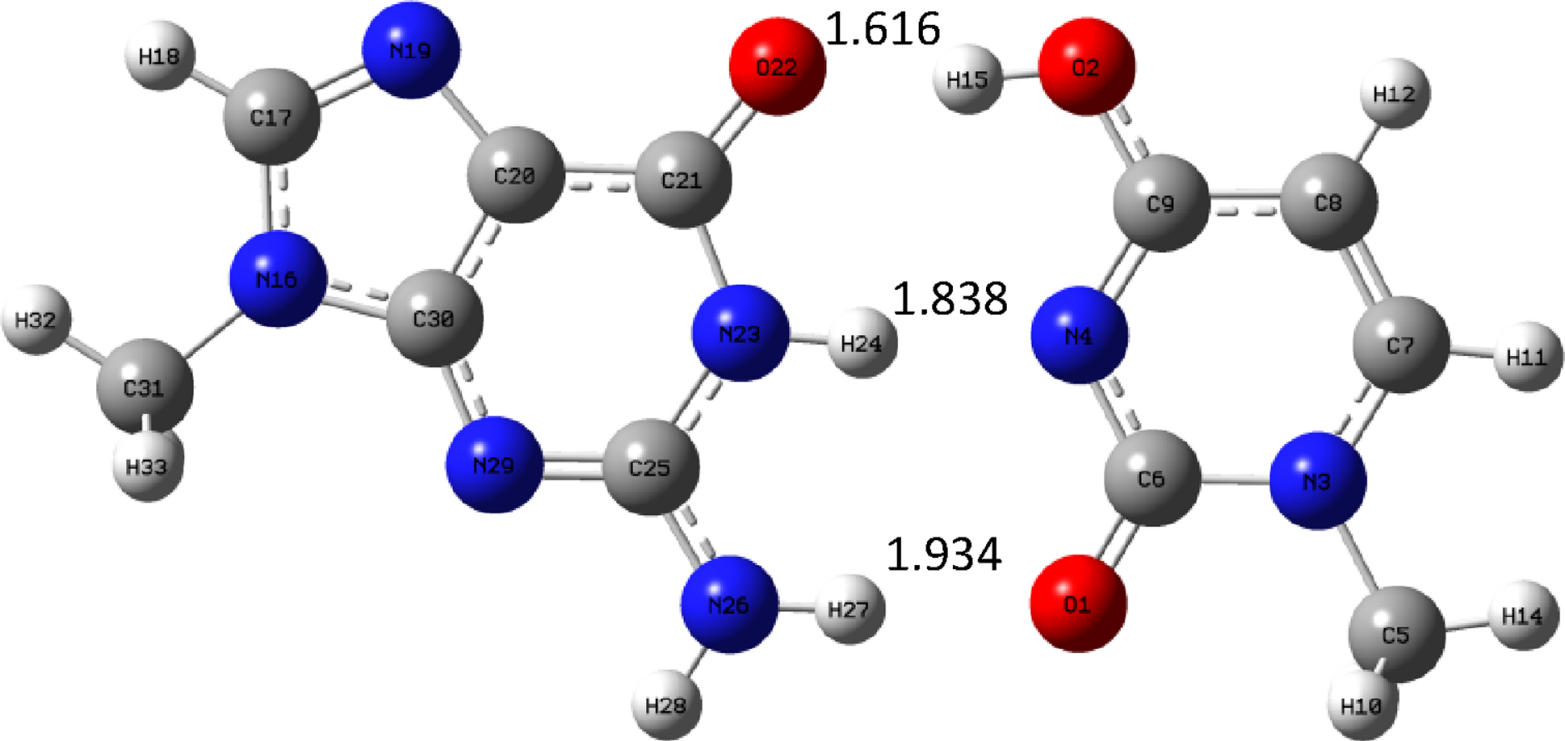
Complex GU_c_ optimized at the B3LYP/aug-cc-pVTZ (PCM) level.

Let us take a look at the trends in hydrogen bond lengths in the complexes with guanine, which are shown in Figures 15–17.

The situation here is different compared to complexes with adenine. The hydrogen bond formed by oxygen atom of guanine is longest in the natural complex GC (Figure 16) and shortest in complex GU_c_ (Figure 17). In the molnupravir complex GM_c_ (Figure 15), it is also quite long but shorter than in GC. Next comes the hydrogen bond between the N–H group of guanine and the nitrogen atom of pyramidic bases. Here the situation is the same: the bond is longest in the GC complex, a little bit shorter in GM_c_, and much shorter in GU_c_ complex. In the case of the last hydrogen bond, the situation is different: it is shortest in the GC complex and longest in the GU_c_ complex. These trends in hydrogen bond lengths are reflected in binding enthalpies, as the GU_c_ complex is strongest and GC and GM_c_ have similar binding enthalpy.

**Figure 18:**
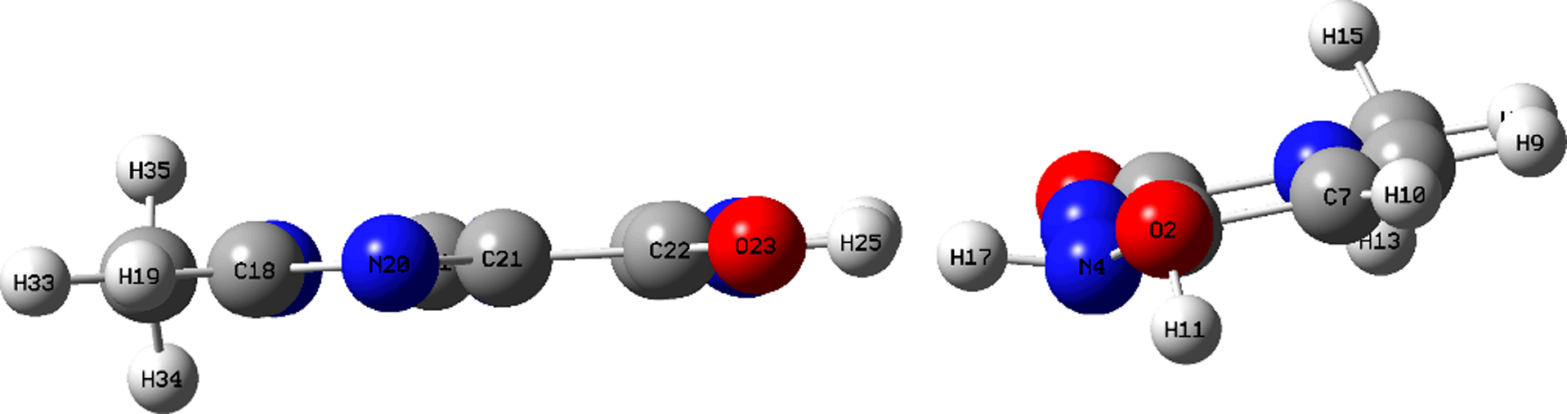
Different view of complex GM_c_ optimized at the B3LYP/aug-cc-pVTZ (PCM) level.

Finally, it is also worth noting that the geometry of the GM_c_ complex is rather unusual (Figure 18).

It follows that the GM_c_ complex is distorted out of plane, and we can describe this geometry as indeed suboptimal for complex formation. We believe that the results of complex energetics and structure confirm the conclusion that molnupiravir can compete with cytosine in complex formation, but its structure is suboptimal, and after building into the RNA strand, it can easily tautomerize during replication to its uracil-like form, causing mutations.

**Figure 19:**
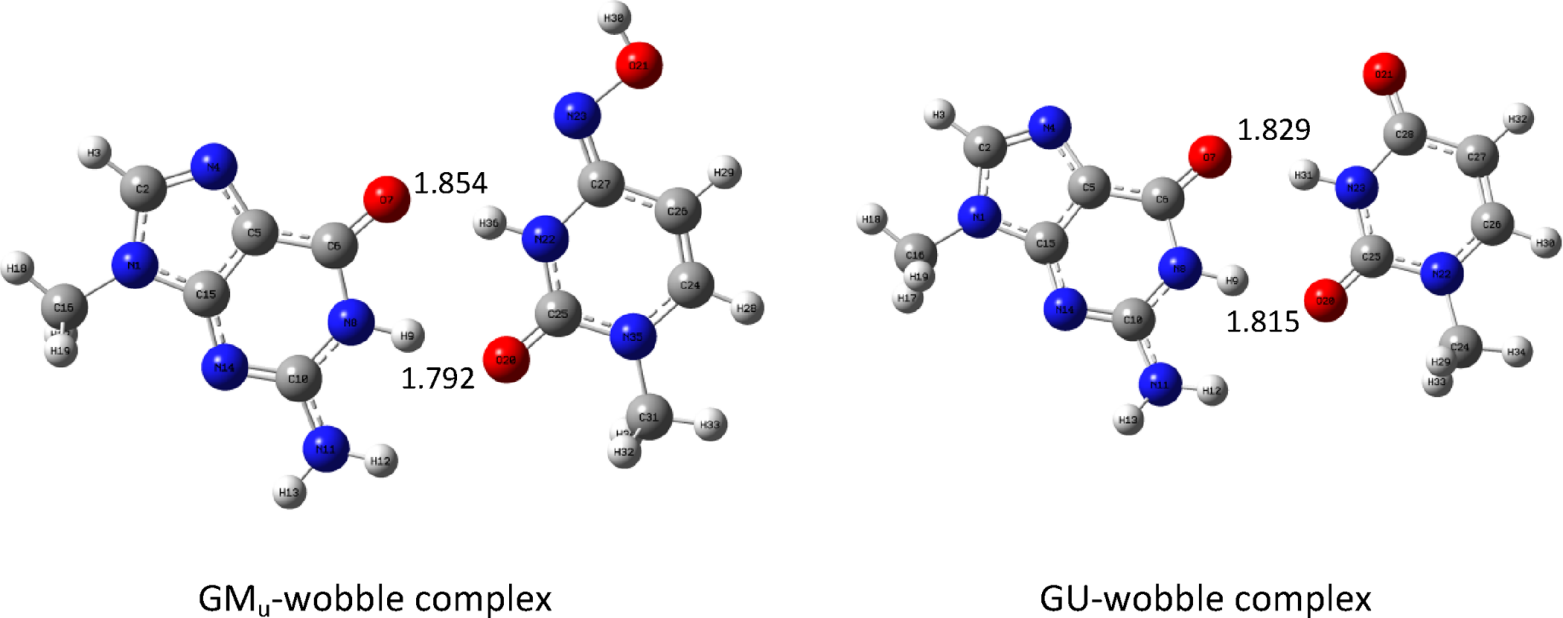
Guanine “wobble” complexes optimized at the B3LYP/aug-cc-pVTZ (PCM) level.

In the case of guanine, there is the possibility of forming the so-called “wobble” complexes with only two hydrogen bonds. Such complexes for M_u_ and uracil were also explored. The proper complexes were optimized, and the structures are shown in Figure 19 below.

It follows that one of the hydrogen bonds (the lower one in Figure 19) is shorter in the case of the GM_u_ complex but that the second is longer. At the B3LYP/aug-cc-pVTZ level, the GU complex is more stable, and its binding enthalpy is equal to −11.75 kcal/mol for the gas phase and −6.67 kcal/mol for the water environment. For the GM_u_ complex, the appropriate values are −9.77 kcal/mol for the gas phase and −4.3 kcal/mol for the water environment. It follows that these complexes are more than two times weaker compared to normal guanine complexes with three hydrogen bonds (Tables 10 and 11).

Another question pointed out by Gordon et al. is the influence of the molnupiravir built into the RNA strand on its further extension. If M_c_ is paired with guanine, it inhibits the incorporation of the next incoming nucleotide. However, if, after tautomerization of M_c_ → M_u_ within the RNA strand, molnupiravir (M_u_) pairs with adenine, no inhibition is observed. This observation also confirms the fact that molnupiravir builds into RNA as a cytosine analogue but then effectively pairs with adenine causing G → A and C → U mutations.

## 3 Conclusions

Following are the conclusion:

1. From all possible tautomeric forms of molnupiravir, only two forms—M_c_ mimicking cytosine and M_u_ mimicking uracil—can be built into the RNA strand.
2. The uracil-like M_u_ form is more stable in the gas phase by 7.26 kcal/mol and in the water environment by 3.06 kcal/mol at the CCSD(T)/aug-cc-pVTZ//B3LYP/aug-cc-pVTZ level of theory. These values reflect tautomer stability in the RNA strand.
3. There exists also a lower energy (about 3 kcal/mol) geometric isomer M_u_-m that is unable to form a basic pair with adenine, which means that part of M_u_ is unable to compete with uracil. In the case of M_c_, the second isomer is a rotamer that is only a little more stable (less than 1 kcal/mol), so the cytosine-like molnupiravir is available for building into RNA.
4. The energy difference between M_c_ and M_u_ tautomers of molnupiravir is greatly reduced when it is in its monophosphate form (MMP), i.e., to 3.99 kcal/mol in the gas phase and to 0.96 kcal/mol in the water environment at the B3LYP/6-311++G(d,p) level of theory. These values reflect the relative stability of tautomers as free, active substrates to RNA replication.
5. The above points enable us to conclude that, after its administration *in vivo*, molnupiravir exists in both cytosine-like and uracil-like forms, and both forms can be incorporated into the RNA strand. However, most of the uracil-like form is not available for pairing with adenine (M_u_-m). After building M_c_ into the RNA stand, the energy gap between the tautomers increases in favor of M_u_, and the probability of M_c_ → M_u_ interconversion with accompanying G → A and C → U mutations is high.
6. The data presented explain the experimentally observed tendency that molnupiravir seems to compete mainly with cytosine.
7. Kinetic calculations confirm that the molnupiravir tautomers' interconversion should be easy with the Gibbs free energy of activation equal to 18.3 kcal/mol for the gas phase and 16.6 kcal/mol in the water environment; however, proton transfer in M_u_-m has a higher activation barrier of 23.6 kcal/mol in the gas phase and 21.6 kcal/mol in the water environment, which further limits the availability of the uracil-like tautomer of molnupiravir.
8. Calculation of binding energies of complexes of M_u_ with adenine shows that this complex is weaker than the natural complex with uracil. On the other hand, the complex of M_c_ with guanine is of similar strength to the natural complex with cytosine. These calculations are in line with the concept that the complexes are forming mainly between the cytosine-like form of molnupiravir and then the mutations are done after M_c_ is built into the RNA strand.
9. Of all analyzed purine-pyrimidine complexes, only the GM_c_ complex has a distorted geometry. This is another factor that, after incorporation of M_c_ into the RNA strand, causes its destabilization because of this distorted geometry that facilitates the mutation M_c_ → M_u_
10. The “wobble” complex of GM_u_ is bound more than two times weaker than the GM_c_ complex and is also less stable than the GU “wobble” complex.
11. The aromaticity of molnupiravir tautomers was analyzed via HOMA, pEDA, and NICS indices. It follows that the largest pi excess and lowest aromaticity are connected with the M_u_ tautomer and the smallest pi excess and largest aromaticity are connected with the M_c_ tautomer, which were also confirmed by ACID maps.

## 4 Computational Details

Geometries of all molecules were optimized by using the Gaussian 16 suite of programs^22^ with the B3LYP functional^23,24^ and a set of orbital basis sets of increasing quality: 6-31G(d),^25^ 6-311++G(d,p),^26,27^ aug-cc-pVDZ, and aug-cc-pVTZ.^28,29^ All optimizations were performed in both the gas phase and the water environment modeled via the PCM model^30^ via Gaussian SCRF option. To check the role of the dispersion, additional calculations with B3LYP-D3 with Grimme empirical dispersion correction^31^ were employed. To obtain more accurate energies, the coupled cluster method with single and double excitations with perturbatively included triples CCSD(T)^32^ was used as single point energy calculations, which is denoted as CCSD(T)/aug-cc-pVTZ//B3LYP/aug-cc-pVTZ. In case there was a possibility of conformational isometry, the Avogadro software was used to search for the five lowest energy conformers, and then these conformers were reoptimized at the B3LYP/6-31G(d) level of theory and finally optimized at the B3LYP/6-311++G(d,p) level to obtain the true lowest energy conformer. All geometry optimizations were followed by frequency calculations to establish the nature of the stationary points obtained. Aromaticity indices were calculated according to geometric HOMA,^33,34^ electronic pEDA,^35,36^ and magnetic NICS(1)_ZZ_.^37,38^ HOMA and pEDA indices were calculated using the free AromaTcl software.^39^ The NICS(1)_ZZ_ index was calculated as the *z*-component(perpendicular) of shielding constant of a ghost atom lying 1 Å above the geometric center of the ring. Total atomic charges were calculated according to the NPA scheme by the Natural Bond Orbital (NBO) version 3.1 program interfaced to Gaussian. Localized orbitals were obtained as intrinsic bond orbitals (IBOs) by using the IBO View software.^40^ ACID maps were calculated by using the software package developed by Herges and Geuenich.^41^

## Data Availability

The data underlying this study are available in the published article and its Supporting Information.

## Supporting Information Available

Atom coordinates and absolute energies of calculated structures (PDF)
