# Supplementary material for: Thermodynamic and Kinetic Characteristics of Molnupiravir Tautomers and Its Complexes with RNA Purine Bases as an Explanation of the Possible Mechanism of Action of This Novel Antiviral Medicine: A Quantum-Chemical Study
Source: J Org Chem. 2023 Sep 27;88(19):14048–64. doi: 10.1021/acs.joc.3c01580 (PMC10563131; doi:10.1021/acs.joc.3c01580)
Supplement: Supplementary file 1 — jo3c01580_si_001.pdf [file jo3c01580_si_001.pdf]

Supporting Information

Thermodynamic and kinetic characteristics of molnupiravir tautomers and its complexes with RNA purine bases as an explanation of possible mechanism of action of this novel antiviral medicine. A quantum-chemical study.

Wojciech Piotr Oziminski*, Agata Bycul

Department of Organic and Physical Chemistry, Faculty of Pharmacy, Medical University of Warsaw, 1 Banacha Street, 02-097, Warsaw, Poland

Corresponding Author email: wojciech.oziminski@wum.edu.pl

Table of Contents

Kinetics of M_u and M_u-m in water environment: scheme of reactants and transition states (PCM) 2

Tables with Enthalpy and Gibbs free energy for molecules optimized with all orbital atomic bases, for puric bases and purine-pyrimidine complexes for both the gas phase and the water environment 4

Tables with relative Gibbs free energies of purine-pyrimidine complexes 6

Structures of molnupiravir isomers optimized at the B3LYP/aug-cc-pVTZ from Table 1 8

Structures of molnupiravir isomers optimized at the B3LYP/aug-cc-pVTZ/PCM from Table 2 12

Structures of cytosine and uracil isomers optimized at the B3LYP/aug-cc-pVTZ from Table 4............16

Structures of cytosine and uracil isomers optimized at the B3LYP/aug-cc-pVTZ/PCM from Table 5....22

Structures of M_c and M_u substituted by: methyl, deoxyribose, ribose, ribose monophosphate optimized at B3LYP/6-311++G(d,p) from Table 8...28

Structures of M_c and M_u substituted by: methyl, deoxyribose, ribose, ribose monophosphate optimized at B3LYP/6-311++G(d,p)/PCM from Table 8..39

Structures of reactants, products, Transition States and Transition Product M_{TP} optimized at B3LYP/6-311++G(d,p) from Figures 10-11 and Table 9..49

Structures of reactants, products Transition States and Transition Product M_{TP} optimized at B3LYP/6-311++G(d,p)/PCM from Figures 10-11 and Table 9..61

Structures of purine bases optimized at the B3LYP/aug-cc-pVTZ 73

Structures of purine bases optimized at the B3LYP/aug-cc-pVTZ/PCM 75

Structures of pyrimidine-purine complexes optimized at the B3LYP/aug-cc-pVTZ..............................77

Structures of pyrimidine-purine complexes optimized at the B3LYP/aug-cc-pVTZ/PCM.....................90

Kinetics of M_u and M_u-m in water environment: scheme of reactants and transition states (PCM)

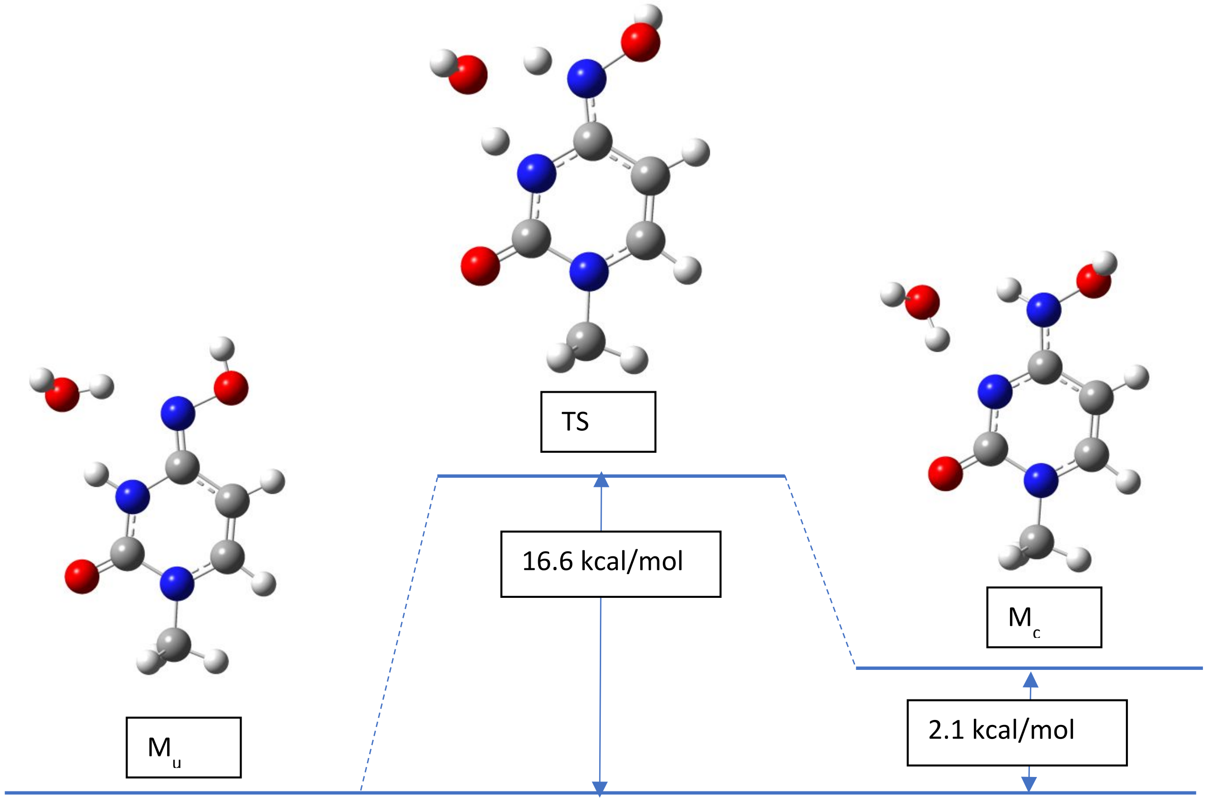

Figure S1. reactant, transition state and product for $M_u \rightarrow M_c$ tautomerization process with single water helper molecule optimized at B3LYP/6-311++G(d,p)/PCM level of theory

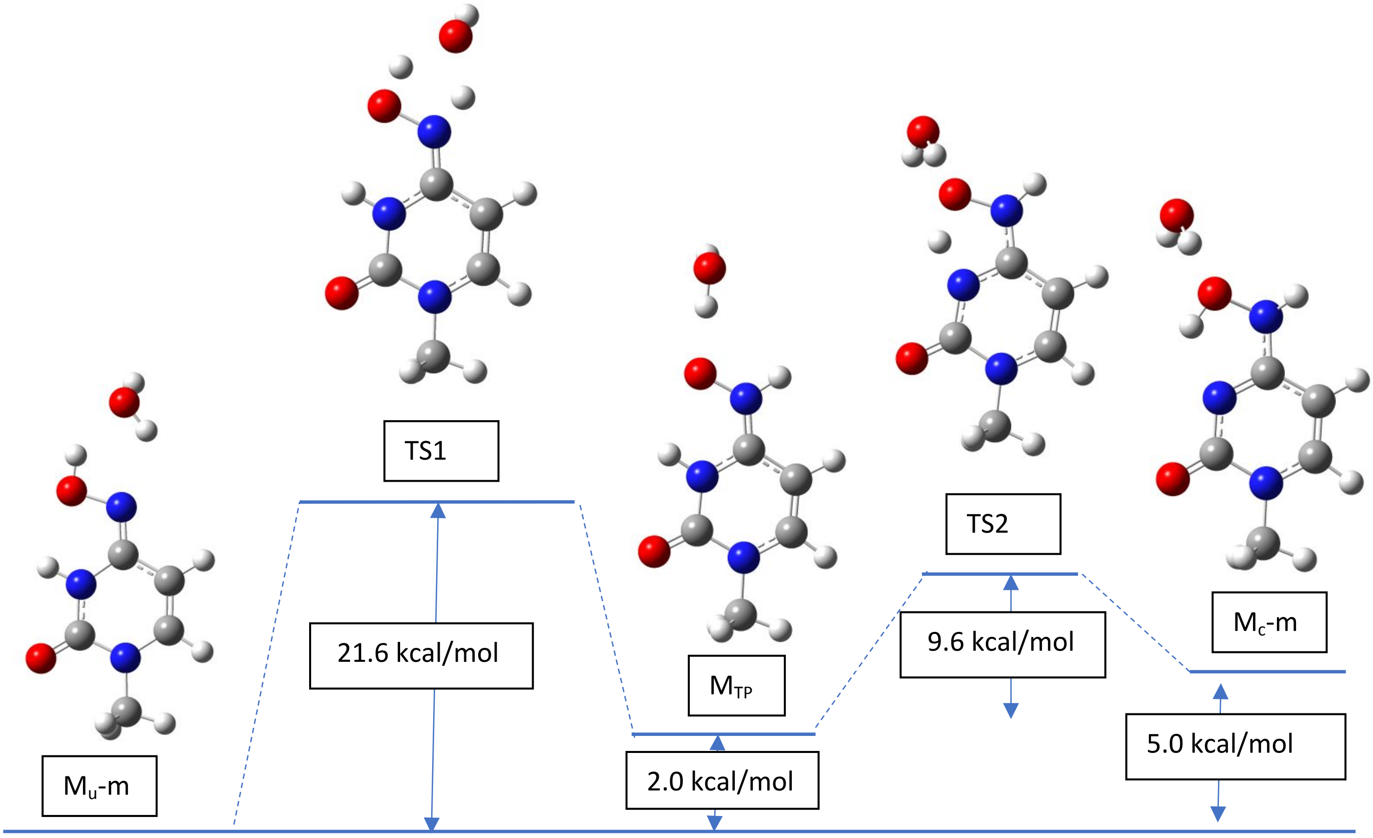

Figure S2. reactant, transition state and product for M$_u$-m→ M$_c$-m tautomerization process with single water helper molecule optimized at B3LYP/6-311++G(d,p)/PCM level of theory

Tables with Enthalpy and Gibbs free energy for molecules optimized with all orbital atomic bases, for puric bases and purine-pyrimidine complexes for both the gas phase and the water environment

Table S1. Gas-phase Gibbs free energies of molnupiravir and its isomers

	6-31G(d)	6-311++G(d,p)	aug-cc-pVDZ	aug-cc-pVTZ
M_c	-509.29971	-509.458852	-509.383968	-509.507138
M_c-m	-509.300706	-509.460057	-509.385384	-509.508902
M_u	-509.310019	-509.47033	-509.39412	-509.518032
M_u-m	-509.315883	-509.474437	-509.398133	-509.521923

Table S2. Water environment (PCM) Gibbs free energies of molnupiravir and its isomers

	6-31G(d)	6-311++G(d,p)	aug-cc-pVDZ	aug-cc-pVTZ
M_c	-509.317739	-509.479626	-509.403796	-509.526811
M_c-m	-509.317933	-509.479638	-509.404006	-509.52726
M_u	-509.321832	-509.483853	-509.407233	-509.530729
M_u-m	-509.325765	-509.487725	-509.411047	-509.534443

Table S3 Gas-phase Gibbs free energies of cytosine and uracil and their isomers

	6-31G(d)	6-311++G(d,p)	aug-cc-pVDZ	aug-cc-pVTZ
C	-434.146337	-434.279186	-434.214771	-434.321451
Cu	-434.143007	-434.276258	-434.209776	-434.316758
Cu-h	-434.140528	-434.273144	-434.207433	-434.314274
U	-454.045236	-454.183353	-454.113079	-454.22517
Uc	-454.02463	-454.164117	-454.095942	-454.207476
Uc-h	-454.013797	-454.153658	-454.086538	-454.198113

Table S4. Water environment (PCM) Gibbs free energies of cytosine and uracil and their isomers

	6-31G(d)	6-311++G(d,p)	aug-cc-pVDZ	aug-cc-pVTZ
C	-434.165224	-434.301488	-434.235151	-434.341858
Cu	-434.155431	-434.290053	-434.223876	-434.330585
Cu-h	-434.153613	-434.288327	-434.2222	-434.328809

U	-454.058233	-454.19884	-454.12857	-454.240364
Uc	-454.039363	-454.181231	-454.112405	-454.223705
Uc-h	-454.034913	-454.177486	-454.108984	-454.220382

Table S5. Gas-phase Enthapy of puric bases

Nazwa/baza	6-31G(d)	6-311++G(d,p)	aug-cc-pVDZ	aug-cc-pVTZ
guanine	-581.707758	-581.878345	-581.796581	-581.933444
adenine	-506.481907	-506.622957	-506.55306	-506.671371

Table S6. Gas-phase Gibbs free energy of puric bases

Nazwa/baza	6-31G(d)	6-311++G(d,p)	aug-cc-pVDZ	aug-cc-pVTZ
guanine	-581.754985	-581.925885	-581.843448	-581.980221
adenine	-506.526445	-506.669557	-506.598068	-506.7171

Table S7. Water environment (PCM) Enthapy of puric bases

Nazwa/baza	6-31G(d)	6-311++G(d,p)	aug-cc-pVDZ	aug-cc-pVTZ
guanine	-581.730996	-581.904444	-581.821956	-581.95862
adenine	-506.494101	-506.636525	-506.566151	-506.685203

Table S8. Water environment (PCM) Gibbs free energy of puric bases

Nazwa/baza	6-31G(d)	6-311++G(d,p)	aug-cc-pVDZ	aug-cc-pVTZ
guanine	-581.777628	-581.951136	-581.868529	-582.005158
adenine	-506.53852	-506.681308	-506.611419	-506.728714

Tables with relative Gibbs free energies of purine-pyrimidine complexes

Table S9. Gas phase Gibbs Free Energy of complex formation from base pairs in conformation of pyrimidine bases “as in complex”. Relative values are given in kcal/mol.

	6-31G(d)	6-311++G(d,p)	aug-cc-pVDZ	**aug-cc-pVTZ**
AM_u	-2.44	1.09	1.82	**2.86 (5.3)**
AU	-3.98	-0.01	-0.13	**1.03**
AC_u	-4.02	0.43	-0.08	**1.14**
GM_c	-15.11	-11.95	-11.65	**-11.02 (-9.91)**
GC	-16.28	-13.47	-11.35	**-10.79**
GU_c	-17.59	-14.93	-13.44	**-12.86**

Values in parentheses compare to lowest energy conformer of molnupiravir

Table S10. Water environment (PCM) Gibbs Free Energy of complex formation from base pairs in conformation of pyrimidine bases “as in complex”. Relative values are given in kcal/mol.

	Gibbs free energy			
	6-31G(d)	6-311++G(d,p)	aug-cc-pVDZ	**aug-cc-pVTZ**
AM_u	-0.14	3.76	5.08	**5.02 (7.35)**
AU	-1.04	3.75	4.69	**4.54**
AC_u	-1.14	3.54	4.40	**4.48**
GM_c	-5.79	-0.55	-0.50	**0.17 (0.45)**
GC	-6.08	-0.15	0.22	**0.86**
GU_c	-8.32	-3.72	-2.90	**-2.24**

Values in parentheses compare to lowest energy conformer of molnupiravir

Table S11. Gas-phase Enthalpy of RNA bases complexes

	6-31G(d)	6-311++G(d,p)	aug-cc-pVDZ	aug-cc-pVTZ
AMu	-1015.766881	-1016.06238	-1015.91749	-1016.15772
AU	-960.509276	-960.782386	-960.643487	-960.872097
ACu	-940.60681	-940.874454	-940.739921	-940.963202
GMc	-1091.006554	-1091.330059	-1091.17399	-1091.43236
GC	-1015.857153	-1016.153382	-1016.00736	-1016.24905
GUc	-1015.857153	-1016.153382	-1016.00736	-1036.13948

Table S12. Gas-phase Gibbs free energies of RNA bases complexes

	6-31G(d)	6-311++G(d,p)	aug-cc-pVDZ	aug-cc-pVTZ
AMu	-1015.840353	-1016.13815	-1015.98929	-1016.23058

AU	-960.578021	-960.852922	-960.711355	-960.940626
ACu	-940.675852	-940.945125	-940.807977	-941.032039
GMc	-1091.078774	-1091.403778	-1091.24598	-1091.50492
GC	-1015.927268	-1016.22654	-1016.07631	-1016.31887
GUc	-1035.80765	-1036.113788	-1035.96081	-1036.20819

Table S13. Water environment (PCM) Enthalpy of RNA bases complexes

	6-31G(d)	6-311++G(d,p)	aug-cc-pVDZ	aug-cc-pVTZ
AMu	-1015.786957	-1016.084923	-1015.93902	-1016.17899
AU	-960.52827	-960.804387	-960.664698	-960.893094
ACu	-940.62601	-940.895953	-940.760453	-940.983497
GMc	-1091.032499	-1091.35884	-1091.20127	-1091.45937
GC	-1015.882687	-1016.181836	-1016.03454	-1016.27591
GUc	-1035.761658	-1036.06802	-1035.91794	-1036.16416

Table S14. Water environment (PCM) Gibbs free energies of RNA bases complexes

	6-31G(d)	6-311++G(d,p)	aug-cc-pVDZ	aug-cc-pVTZ
AMu	-1015.860574	-1016.159172	-1016.01055	-1016.25144
AU	-960.598406	-960.874171	-960.73251	-960.961837
ACu	-940.695766	-940.965727	-940.828277	-941.052157
GMc	-1091.1046	-1091.431632	-1091.27312	-1091.5317
GC	-1015.952543	-1016.252867	-1016.10334	-1016.34565
GUc	-1035.830252	-1036.138288	-1035.98555	-1036.23244

Structures of molnupiravir isomers optimized at the B3LYP/aug-cc-pVTZ from Table 1

M_c

	angstroms		
	X	Y	Z
O	-2.001817000	-1.720096000	0.036583000
O	3.389413000	0.295966000	0.279092000
N	0.219800000	-1.159149000	-0.018156000
N	2.413697000	-0.607185000	-0.197088000
C	-0.559855000	1.472262000	-0.031858000
C	-1.118048000	-0.884669000	0.003842000
C	0.768169000	1.190206000	-0.074862000
C	1.100195000	-0.190835000	-0.073294000
H	-0.929464000	2.488379000	-0.022186000
H	1.513675000	1.964856000	-0.091598000
H	3.952511000	0.460879000	-0.485896000
C	-2.918540000	0.795433000	0.045667000
H	-3.418019000	0.356718000	-0.815808000
H	-3.055161000	1.874125000	0.045082000
H	-3.360906000	0.364908000	0.941800000
N	-1.491110000	0.497712000	0.000669000
H	2.568372000	-1.530867000	0.188261000

Gibbs free energy = -509.507138 Hartree

Number of imaginary frequencies: 0

M_c-m

	X	Y	Z
		angstroms	
O	-1.377780000	-1.966869000	-0.007374000
O	3.143440000	-0.785177000	0.067569000
N	0.572952000	-0.767530000	-0.028500000
N	2.493418000	0.442790000	-0.117128000
C	-0.950015000	1.515147000	0.016083000
C	-0.788577000	-0.904047000	-0.013512000
C	0.398666000	1.643457000	-0.001200000
C	1.132373000	0.421925000	-0.031986000
H	-1.606497000	2.373609000	0.038198000
H	0.875185000	2.610279000	-0.007311000
H	2.384243000	-1.408945000	0.103292000
C	-3.004135000	0.164344000	0.020427000
H	-3.318380000	-0.388214000	0.903779000
H	-3.336348000	-0.388927000	-0.855674000
H	-3.452788000	1.154676000	0.024346000
N	-1.551440000	0.302638000	0.004114000
H	2.994925000	1.173646000	0.363537000

Gibbs free energy = -509.508902 Hartree

Number of imaginary frequencies: 0

M_u

	X	Y	Z
	angstroms		
O	1.992965000	1.737841000	0.001991000
O	-3.392827000	-0.358431000	0.003596000
N	-0.192133000	1.122322000	-0.002833000
N	-2.427239000	0.679438000	0.000873000
C	0.492343000	-1.465736000	-0.003233000
C	1.155052000	0.856076000	-0.002357000
C	-0.821085000	-1.186177000	-0.000696000
C	-1.237972000	0.194703000	-0.000674000
H	0.855307000	-2.483715000	-0.003607000
H	-1.562233000	-1.964983000	0.000025000
H	-4.217775000	0.134855000	0.003832000
C	2.883876000	-0.893527000	0.006452000
H	3.484297000	0.004364000	-0.086754000
H	3.095762000	-1.560632000	-0.828982000
H	3.133592000	-1.396683000	0.941533000
N	1.478048000	-0.502026000	-0.007710000
H	-0.434075000	2.101347000	-0.000009000

Gibbs free energy = -509.518032 Hartree

Number of imaginary frequencies: 0

M_u-m

	angstroms		
	X	Y	Z
O	1.332365000	-2.007924000	0.000007000
O	-3.088798000	-0.732388000	0.000005000
N	-0.545496000	-0.729532000	-0.000017000
N	-2.515359000	0.569333000	0.000012000
C	0.930433000	1.511682000	-0.000005000
C	0.824361000	-0.903418000	-0.000026000
C	-0.402177000	1.657178000	0.000003000
C	-1.230957000	0.478260000	-0.000001000
H	1.598407000	2.360343000	0.000002000
H	-0.868491000	2.627636000	0.000013000
H	-4.031320000	-0.545114000	0.000015000
C	3.015256000	0.205609000	0.000028000
H	3.419251000	0.690569000	0.889238000
H	3.419312000	0.691091000	-0.888867000
H	3.301566000	-0.840008000	-0.000261000
N	1.558040000	0.277446000	-0.000027000
H	-1.089056000	-1.578613000	-0.000006000

Gibbs free energy = -509.521923 Hartree

Number of imaginary frequencies: 0

Structures of molnupiravir isomers optimized at the B3LYP/aug-cc-pVTZ/PCM from Table 2

M_c

	angstroms		
	X	Y	Z
O	-2.001465000	-1.729353000	0.028486000
O	3.393512000	0.304338000	0.248582000
N	0.210174000	-1.163181000	-0.017397000
N	2.410735000	-0.608724000	-0.166672000
C	-0.557965000	1.465469000	-0.025647000
C	-1.116913000	-0.872327000	0.002190000
C	0.769925000	1.181281000	-0.060736000
C	1.106783000	-0.194257000	-0.059906000
H	-0.926666000	2.480494000	-0.019879000
H	1.512554000	1.958398000	-0.084106000
H	3.918987000	0.478979000	-0.542751000
C	-2.919905000	0.807614000	0.038259000
H	-3.416552000	0.388154000	-0.833624000
H	-3.038194000	1.886669000	0.043260000
H	-3.372319000	0.385004000	0.932310000
N	-1.492560000	0.490595000	0.002209000
H	2.595820000	-1.535084000	0.196294000

Gibbs free energy = -509.526811 Hartree

Number of imaginary frequencies: 0

M_c-m

	angstroms		
	X	Y	Z
O	-1.367341000	-1.977957000	-0.009511000
O	3.142122000	-0.784078000	0.072920000
N	0.568470000	-0.768094000	-0.032732000
N	2.491630000	0.446192000	-0.120912000
C	-0.950123000	1.509584000	0.017599000
C	-0.789161000	-0.892758000	-0.014431000
C	0.397966000	1.640781000	-0.001579000
C	1.133359000	0.426549000	-0.035472000
H	-1.609148000	2.364144000	0.044651000
H	0.872670000	2.608057000	0.005199000
H	2.384133000	-1.405339000	0.135532000
C	-3.007945000	0.170001000	0.022295000
H	-3.327270000	-0.371402000	0.910014000
H	-3.347267000	-0.372403000	-0.857161000
H	-3.440766000	1.165509000	0.026340000
N	-1.550438000	0.292677000	0.004507000
H	2.997185000	1.187353000	0.341627000

Gibbs free energy = -509.527260 Hartree

Number of imaginary frequencies: 0

M_u

	angstroms		
	X	Y	Z
O	2.002326000	1.717330000	0.000708000
O	-3.399785000	-0.352882000	-0.000021000
N	-0.190769000	1.119984000	-0.000108000
N	-2.428454000	0.687839000	0.000010000
C	0.490042000	-1.469767000	-0.000259000
C	1.150191000	0.838489000	-0.000204000
C	-0.824347000	-1.182724000	-0.000160000
C	-1.237584000	0.196143000	-0.000068000
H	0.846735000	-2.488511000	-0.000235000
H	-1.560729000	-1.965727000	-0.000120000
H	-4.227971000	0.137600000	-0.000048000
C	2.892564000	-0.867062000	0.000289000
H	3.385214000	-0.464689000	-0.882091000
H	2.972397000	-1.949203000	-0.004596000
H	3.382634000	-0.472834000	0.887860000
N	1.473915000	-0.508387000	-0.000501000
H	-0.426648000	2.101255000	0.000342000

Gibbs free energy = -509.530729 Hartree

Number of imaginary frequencies: 0

M_u-m

	angstroms		
	X	Y	Z
O	-1.346082000	-1.989700000	0.000055000
O	3.087287000	-0.748939000	0.000038000
N	0.544343000	-0.724642000	-0.000052000
N	2.521405000	0.560246000	0.000035000
C	-0.927847000	1.518954000	-0.000014000
C	-0.824376000	-0.882685000	-0.000114000
C	0.407097000	1.658778000	-0.000025000
C	1.232724000	0.480013000	-0.000013000
H	-1.591065000	2.369644000	0.000022000
H	0.868026000	2.631985000	-0.000002000
H	4.033847000	-0.575296000	0.000074000
C	-3.014061000	0.179584000	0.000050000
H	-3.355782000	-0.352835000	-0.884955000
H	-3.430334000	1.181629000	0.000050000
H	-3.355694000	-0.352797000	0.885115000
N	-1.554871000	0.286605000	-0.000035000
H	1.084001000	-1.576550000	0.000009000

Gibbs free energy = -509.534443 Hartree

Number of imaginary frequencies: 0

Structures of cytosine and uracil isomers optimized at the B3LYP/aug-cc-pVTZ from Table 4

C

	angstroms		
	X	Y	Z
O	-1.431645000	-1.809717000	0.000055000
N	-1.114012000	0.442019000	0.000236000
N	0.734627000	-1.066672000	0.001217000
N	2.875845000	-0.281371000	-0.024483000
C	-2.561779000	0.616256000	0.001592000
C	-0.268142000	1.493211000	0.000413000
C	-0.619393000	-0.902409000	0.000282000
C	1.076543000	1.321963000	-0.001177000
C	1.541606000	-0.029682000	-0.002304000
H	-2.996419000	0.142443000	-0.876339000
H	-0.721542000	2.474868000	0.000947000
H	1.746796000	2.166585000	-0.006466000
H	3.174477000	-1.236951000	0.062646000
H	3.546400000	0.451859000	0.105126000
H	-2.790050000	1.679567000	-0.000040000
H	-2.994734000	0.145506000	0.882053000

Gibbs free energy = -434.321451 Hartree

Number of imaginary frequencies: 0

C_u

	X	Y	Z
	angstroms		
O	-1.425157000	-1.804509000	0.000099000
N	-1.094243000	0.456560000	-0.000118000
N	0.713290000	-1.015226000	0.000208000
N	2.915833000	-0.383429000	-0.000057000
C	-2.537530000	0.675350000	-0.000105000
C	-0.198610000	1.499669000	-0.000136000
C	-0.652283000	-0.865895000	0.000224000
C	1.133784000	1.325418000	-0.000054000
C	1.692241000	-0.013855000	0.000024000
H	-2.991141000	0.224877000	-0.880993000
H	-0.642917000	2.484528000	-0.000273000
H	1.797544000	2.174752000	-0.000139000
H	3.538517000	0.417770000	-0.000158000
H	-2.724665000	1.745632000	-0.001716000
H	-2.990852000	0.227592000	0.882342000
H	1.044996000	-1.968536000	0.000206000

Gibbs free energy = -434.316758 Hartree

Number of imaginary frequencies: 0

C_u-h

	angstroms		
	X	Y	Z
O	1.390066000	-1.825034000	0.000058000
N	1.103310000	0.442186000	0.000248000
N	-0.733025000	-0.995457000	-0.000255000
N	-2.962164000	-0.160010000	0.000219000
C	2.549831000	0.638496000	-0.000016000
C	0.221896000	1.503994000	-0.000095000
C	0.641634000	-0.865953000	-0.000037000
C	-1.110899000	1.352546000	0.000033000
C	-1.698304000	0.027636000	0.000153000
H	2.997055000	0.181330000	0.880766000
H	0.684172000	2.480047000	-0.000612000
H	-1.773289000	2.201437000	-0.000380000
H	-3.213530000	-1.146312000	-0.000916000
H	2.752997000	1.705752000	0.002068000
H	2.996462000	0.184859000	-0.882969000
H	-1.046194000	-1.954184000	-0.000136000

Gibbs free energy = -434.314274 Hartree

Number of imaginary frequencies: 0

U

	angstroms		
	X	Y	Z
O	-1.408408000	-1.808621000	0.000047000
O	2.891438000	-0.288536000	0.000022000
N	-1.079991000	0.454102000	-0.000043000
N	0.731952000	-1.009371000	0.000011000
C	-2.525593000	0.668968000	-0.000005000
C	-0.639921000	-0.868371000	-0.000048000
C	-0.190735000	1.498072000	-0.000033000
C	1.145153000	1.334756000	-0.000016000
C	1.710411000	-0.000664000	0.000052000
H	-2.975213000	0.216652000	-0.881637000
H	-0.641820000	2.479975000	-0.000047000
H	1.068430000	-1.961740000	0.000049000
H	1.815987000	2.177235000	-0.000030000
H	-2.975131000	0.217093000	0.881900000
H	-2.716120000	1.738365000	-0.000261000

Gibbs free energy = -454.225170 Hartree

Number of imaginary frequencies: 0

U_c

	angstroms		
	X	Y	Z
O	1.402825000	-1.817029000	0.000005000
O	-2.866634000	-0.212580000	-0.000039000
N	1.096953000	0.439075000	-0.000043000
N	-0.759740000	-1.052798000	-0.000009000
C	2.547237000	0.605716000	0.000006000
C	0.603662000	-0.902208000	0.000004000
C	0.257605000	1.496373000	-0.000011000
C	-1.090348000	1.334035000	0.000032000
C	-1.539020000	-0.011068000	0.000094000
H	2.976370000	0.130460000	0.879668000
H	0.718747000	2.473936000	-0.000070000
H	-1.773744000	2.165704000	-0.000006000
H	2.976464000	0.130093000	-0.879413000
H	2.781201000	1.667310000	-0.000213000
H	-3.003878000	-1.171658000	-0.000090000

Gibbs free energy = -454.207476 Hartree

Number of imaginary frequencies: 0

U_c-h

	X	Y	Z
O	-1.456172000	-1.789283000	0.000082000
O	2.851450000	-0.382769000	0.000025000
N	-1.091911000	0.456001000	-0.000032000
N	0.727482000	-1.091409000	0.000057000
C	-2.537249000	0.659223000	-0.000049000
C	-0.628838000	-0.900560000	0.000037000
C	-0.226566000	1.487110000	-0.000070000
C	1.116636000	1.282362000	-0.000050000
C	1.535921000	-0.078574000	0.000023000
H	-2.977548000	0.193754000	-0.879378000
H	-0.656781000	2.478875000	-0.000121000
H	1.807276000	2.111011000	-0.000087000
H	-2.977550000	0.193919000	0.879368000
H	-2.745029000	1.726426000	-0.000148000
H	3.378988000	0.422907000	-0.000019000

(Coordinates in angstroms)

Gibbs free energy = -454.198113 Hartree

Number of imaginary frequencies: 0

Structures of cytosine and uracil isomers optimized at the B3LYP/aug-cc-pVTZ/PCM from Table 5

C

	angstroms		
	X	Y	Z
O	1.427270000	-1.822363000	0.000219000
N	1.116084000	0.431866000	-0.000058000
N	-0.726761000	-1.069141000	0.000000000
N	-2.872728000	-0.275879000	0.000220000
C	2.564812000	0.625915000	-0.000015000
C	0.267128000	1.487164000	-0.000064000
C	0.618591000	-0.889588000	-0.000031000
C	-1.075983000	1.316962000	-0.000043000
C	-1.549340000	-0.028227000	0.000001000
H	3.004788000	0.168423000	0.883355000
H	0.722897000	2.466126000	-0.000091000
H	-1.746009000	2.161104000	-0.000034000
H	-3.200363000	-1.225916000	-0.000785000
H	-3.545510000	0.468659000	-0.001045000
H	2.773741000	1.691447000	-0.000382000
H	3.004889000	0.167789000	-0.882998000

Gibbs free energy = -434.341858 Hartree

Number of imaginary frequencies: 0

C_u

	angstroms		
	X	Y	Z
O	1.417964000	-1.814075000	-0.000076000
N	1.094820000	0.450097000	0.000167000
N	-0.713135000	-1.015087000	-0.000127000
N	-2.919609000	-0.375461000	-0.000307000
C	2.539773000	0.681461000	0.000205000
C	0.200866000	1.494907000	0.000116000
C	0.650719000	-0.860022000	0.000192000
C	-1.132652000	1.321269000	-0.000002000
C	-1.686878000	-0.013882000	-0.000119000
H	2.993559000	0.240867000	0.885186000
H	0.646688000	2.477936000	0.000145000
H	-1.795595000	2.171045000	-0.000065000
H	-3.523289000	0.440849000	-0.000349000
H	2.714954000	1.752285000	0.000297000
H	2.993605000	0.241013000	-0.884827000
H	-1.039140000	-1.970642000	-0.000270000

Gibbs free energy = -434.330585 Hartree

Number of imaginary frequencies: 0

C_u-h

	angstroms		
	X	Y	Z
O	1.371201000	-1.837767000	0.000114000
N	1.105210000	0.433340000	0.000101000
N	-0.740669000	-0.987057000	-0.000246000
N	-2.962092000	-0.155010000	0.000221000
C	2.555210000	0.630354000	0.000001000
C	0.235146000	1.500567000	-0.000077000
C	0.630792000	-0.862784000	-0.000066000
C	-1.100884000	1.358631000	0.000090000
C	-1.692481000	0.040624000	0.000103000
H	2.998712000	0.177921000	0.884242000
H	0.704536000	2.472309000	-0.000510000
H	-1.748474000	2.219143000	-0.000224000
H	-3.195339000	-1.145670000	-0.001020000
H	2.755579000	1.696784000	0.001770000
H	2.998316000	0.180893000	-0.885992000
H	-1.066794000	-1.942507000	-0.000011000

Gibbs free energy = -434.328809 Hartree

Number of imaginary frequencies: 0

U

	angstroms		
	X	Y	Z
O	-1.391820000	-1.821018000	0.000120000
O	2.893670000	-0.279773000	0.000043000
N	-1.081867000	0.446496000	-0.000068000
N	0.737436000	-1.003122000	0.000025000
C	-2.530743000	0.665956000	0.000000000
C	-0.633194000	-0.863616000	-0.000147000
C	-0.201018000	1.493066000	-0.000070000
C	1.138678000	1.334389000	-0.000057000
C	1.700437000	0.008168000	0.000111000
H	-2.976891000	0.219057000	-0.885128000
H	-0.655354000	2.472239000	-0.000088000
H	1.076136000	-1.955205000	0.000116000
H	1.799378000	2.184777000	-0.000095000
H	-2.976806000	0.219203000	0.885247000
H	-2.715200000	1.734870000	-0.000079000

Gibbs free energy = -454.240364 Hartree

Number of imaginary frequencies: 0

U_c

	angstroms		
	X	Y	Z
O	1.392482000	-1.830536000	0.000530000
O	-2.867190000	-0.205083000	0.000075000
N	1.099409000	0.429371000	-0.000164000
N	-0.756283000	-1.051396000	-0.000176000
C	2.551971000	0.609042000	-0.000386000
C	0.600381000	-0.891314000	0.000040000
C	0.263024000	1.490339000	0.000236000
C	-1.086537000	1.331220000	0.000295000
C	-1.542022000	-0.005093000	-0.000188000
H	2.983173000	0.144351000	0.883061000
H	0.727370000	2.464895000	0.000345000
H	-1.762253000	2.169152000	0.000796000
H	2.982823000	0.144581000	-0.884136000
H	2.772089000	1.671823000	-0.000320000
H	-3.028332000	-1.160835000	-0.002198000

Gibbs free energy = -454.223705 Hartree

Number of imaginary frequencies: 0

U_c-h

	angstroms		
	X	Y	Z
O	-1.444148000	-1.805330000	0.000533000
O	2.846398000	-0.382675000	0.000194000
N	-1.093563000	0.445002000	-0.000174000
N	0.723812000	-1.086118000	0.000026000
C	-2.540925000	0.662457000	0.000001000
C	-0.626437000	-0.888157000	-0.000331000
C	-0.230167000	1.481714000	-0.000209000
C	1.114680000	1.284145000	-0.000204000
C	1.539863000	-0.065677000	-0.000041000
H	-2.983538000	0.208741000	-0.883428000
H	-0.666112000	2.469358000	-0.000191000
H	1.802232000	2.114255000	-0.000197000
H	-2.983349000	0.208699000	0.883506000
H	-2.733507000	1.730540000	0.000050000
H	3.392444000	0.413372000	0.000171000

Gibbs free energy = -454.220382 Hartree

Number of imaginary frequencies: 0

Structures of M_c and M_u substituted by: methyl, deoxyribose, ribose, ribose monophosphate optimized at B3LYP/6-311++G(d,p) from Table 8

M_c

	angstroms		
	X	Y	Z
O	-2.004211000	-1.723333000	0.037190000
O	3.389833000	0.298311000	0.282249000
N	0.221313000	-1.163270000	-0.017138000
N	2.419208000	-0.608496000	-0.200459000
C	-0.559784000	1.477479000	-0.032747000
C	-1.119695000	-0.888137000	0.003019000
C	0.771337000	1.193383000	-0.077852000
C	1.101119000	-0.191827000	-0.075074000
H	-0.930325000	2.495787000	-0.021236000
H	1.521219000	1.967291000	-0.091129000
H	3.965471000	0.452415000	-0.476671000
C	-2.924692000	0.796771000	0.046981000
H	-3.424548000	0.358546000	-0.817549000
H	-3.061794000	1.877934000	0.048142000
H	-3.365779000	0.363907000	0.945507000
N	-1.493262000	0.499012000	0.000239000
H	2.570253000	-1.532438000	0.192956000

Gibbs free energy = -509.458852 Hartree

Number of imaginary frequencies: 0

M_u

	angstroms		
	X	Y	Z
O	1.998683000	1.739479000	0.002936000
O	-3.396255000	-0.358836000	0.005559000
N	-0.192078000	1.125874000	-0.005040000
N	-2.433376000	0.681801000	0.001379000
C	0.491497000	-1.469886000	-0.004694000
C	1.158793000	0.858733000	-0.002702000
C	-0.825098000	-1.188145000	-0.001606000
C	-1.240978000	0.196192000	-0.001404000
H	0.855246000	-2.490014000	-0.005179000
H	-1.571048000	-1.965692000	-0.000602000
H	-4.224571000	0.130283000	0.005764000
C	2.889270000	-0.896440000	0.008905000
H	3.490748000	0.002972000	-0.091704000
H	3.101913000	-1.570458000	-0.823929000
H	3.138866000	-1.391410000	0.951204000
N	1.480110000	-0.503553000	-0.009795000
H	-0.434076000	2.107595000	-0.000308000

Gibbs free energy = -509.470330 Hartree

Number of imaginary frequencies: 0

M_cdRib

angstroms

	X	Y	Z
O	-1.601602000	0.580098000	-0.642512000
O	-3.725031000	-1.846248000	-0.031801000
O	-3.329824000	2.817385000	-0.437792000
O	0.949394000	-2.548331000	-0.409901000
N	0.540024000	-0.308369000	-0.247720000
N	2.766042000	-1.164460000	-0.195478000
N	4.565164000	0.217704000	-0.056536000
C	-3.020248000	-0.833090000	0.686054000
C	-1.539650000	-1.168894000	0.902326000
C	-2.980077000	0.411805000	-0.225760000
C	-0.891570000	-0.618584000	-0.371055000
C	-3.425609000	1.704593000	0.437802000
C	0.999279000	0.951621000	-0.058379000
C	1.431869000	-1.433778000	-0.297468000
C	2.332676000	1.204569000	0.052676000
C	3.187334000	0.071245000	-0.046003000
H	-3.531117000	-0.619202000	1.632341000
H	-1.147497000	-0.628789000	1.769571000
H	-3.588135000	0.217263000	-1.113756000
H	-0.987845000	-1.329157000	-1.195085000
H	-2.836877000	1.872691000	1.351864000
H	-4.477976000	1.622284000	0.719798000

H	-3.729269000	-2.655559000	0.490359000
H	0.246722000	1.725556000	-0.016012000
H	2.711801000	2.201479000	0.207569000
H	5.050216000	-0.620551000	0.247976000
O	5.063605000	1.353795000	0.617071000
H	5.570241000	1.824501000	-0.056064000
H	-2.444410000	2.818257000	-0.818528000
H	-1.350800000	-2.233417000	1.036418000

Gibbs free energy = -891.168647 Hartree

Number of imaginary frequencies: 0

M_udRib

	angstroms		
	X	Y	Z
O	-1.538583000	0.433352000	-0.626730000
O	-3.817344000	-1.724268000	0.556166000
O	-3.301896000	2.571088000	-1.181280000
O	1.065941000	-2.361676000	-1.230151000
N	0.556151000	-0.441552000	-0.058466000
N	2.762240000	-0.987053000	-0.597601000
N	4.580602000	0.266797000	-0.019548000
C	-3.022030000	-0.606316000	0.948980000
C	-1.555698000	-0.970122000	1.236494000
C	-2.932850000	0.336084000	-0.276348000
C	-0.869875000	-0.719325000	-0.112716000
C	-3.448855000	1.745780000	-0.038336000
C	1.022309000	0.714272000	0.549458000
C	1.437334000	-1.343480000	-0.678748000
C	2.325554000	1.038065000	0.616071000
C	3.299702000	0.150965000	0.020752000

H	-3.484799000	-0.102211000	1.805404000
H	-1.138442000	-0.304032000	1.997017000
H	-3.477881000	-0.128659000	-1.104638000
H	-0.988836000	-1.572079000	-0.782208000
H	-2.932609000	2.182136000	0.830288000
H	-4.517991000	1.707608000	0.184155000
H	-3.973022000	-2.285777000	1.322655000
H	0.250364000	1.356593000	0.949205000
H	2.658890000	1.946618000	1.090144000
O	4.997140000	1.450423000	0.636890000
H	5.952166000	1.421399000	0.524418000
H	3.406693000	-1.633766000	-1.032606000
H	-2.382067000	2.517818000	-1.466045000
H	-1.441028000	-1.999886000	1.578712000

Gibbs free energy = -891.179061 Hartree

Number of imaginary frequencies: 0

M_cRib

	angstroms		
	X	Y	Z
O	-1.474565000	0.828893000	-0.718871000
O	-3.733801000	-1.446736000	-0.441060000
O	-1.596653000	-2.462660000	0.713362000
O	-2.894171000	3.218199000	-0.081611000
O	0.899927000	-2.450695000	-0.282483000
N	0.595228000	-0.187035000	-0.286269000
N	2.768922000	-1.146656000	-0.135874000
N	4.637077000	0.142926000	-0.037081000
C	-3.048847000	-0.568982000	0.432293000
C	-1.605939000	-1.059849000	0.673281000
C	-2.862718000	0.783774000	-0.263244000
C	-0.851827000	-0.419186000	-0.515681000
C	-3.106817000	1.999673000	0.615937000
C	1.119450000	1.057954000	-0.167410000
C	1.431889000	-1.341122000	-0.239156000
C	2.460960000	1.249163000	-0.031577000
C	3.258555000	0.073751000	-0.048362000
H	-3.593923000	-0.468903000	1.379167000
H	-1.215206000	-0.626347000	1.604859000
H	-3.501825000	0.828003000	-1.147877000
H	-0.930989000	-1.058982000	-1.400636000

H	-2.474560000	1.946408000	1.514394000
H	-4.151372000	2.006549000	0.935741000
H	-3.406388000	-2.337339000	-0.240955000
H	-0.714205000	-2.752936000	0.403290000
H	0.408790000	1.869502000	-0.197701000
H	2.885955000	2.233692000	0.076892000
H	5.079766000	-0.697131000	0.321728000
O	5.191004000	1.298751000	0.546620000
H	5.733560000	1.683810000	-0.152988000
H	-2.030349000	3.171955000	-0.505485000

Gibbs free energy = -966.413820 Hartree

Number of imaginary frequencies: 0

M_uRib

	angstroms		
	X	Y	Z
O	-1.443844000	0.843945000	-0.694151000
O	-3.740129000	-1.403722000	-0.511553000
O	-1.641441000	-2.474097000	0.670876000
O	-2.858192000	3.239890000	-0.039390000
O	0.926304000	-2.474480000	-0.254815000
N	0.602119000	-0.203091000	-0.245185000
N	2.740087000	-1.121614000	-0.075304000
N	4.659124000	0.093019000	0.154467000
C	-3.060601000	-0.554976000	0.394767000
C	-1.629306000	-1.067965000	0.654109000
C	-2.841824000	0.808897000	-0.269258000
C	-0.844597000	-0.416165000	-0.507642000
C	-3.090482000	2.009896000	0.629466000

C	1.168170000	1.062659000	-0.168057000
C	1.398093000	-1.340790000	-0.196721000
C	2.491815000	1.262732000	-0.029342000
C	3.380087000	0.125098000	0.026328000
H	-3.623678000	-0.466678000	1.332074000
H	-1.250767000	-0.657954000	1.600592000
H	-3.461429000	0.879137000	-1.165959000
H	-0.924744000	-1.034618000	-1.408521000
H	-2.474794000	1.933373000	1.537589000
H	-4.140585000	2.018324000	0.930661000
H	-3.445613000	-2.305804000	-0.314820000
H	-0.759348000	-2.781283000	0.390377000
H	0.459591000	1.872190000	-0.231477000
H	2.904186000	2.256495000	0.033338000
O	5.183824000	1.402652000	0.249664000
H	6.128428000	1.241064000	0.336359000
H	3.313495000	-1.953639000	-0.023609000
H	-1.974361000	3.211384000	-0.421397000

Gibbs free energy = -966.420627 Hartree

Number of imaginary frequencies: 0

M_cMP

	angstroms		
	X	Y	Z
P	-4.437786000	-0.734914000	-0.403943000
O	0.052702000	-0.449963000	1.096397000
O	-2.288050000	1.865661000	-0.320557000
O	0.092745000	2.788355000	-0.196384000
O	-3.573606000	-0.863444000	0.968654000

O	2.798910000	2.347748000	0.121558000
O	-5.851870000	-1.388404000	-0.039056000
O	-4.812934000	0.817849000	-0.468394000
O	-3.766928000	-1.352360000	-1.562484000
N	2.208985000	0.164261000	0.408028000
N	4.438048000	0.772597000	-0.153192000
N	6.077532000	-0.787673000	-0.345840000
C	-1.309743000	0.835509000	-0.332639000
C	0.139092000	1.395613000	-0.402303000
C	-1.309647000	-0.012556000	0.960654000
C	0.865913000	0.649385000	0.751269000
C	-2.191667000	-1.254742000	0.958399000
C	2.532958000	-1.152997000	0.387218000
C	3.173432000	1.175339000	0.119130000
C	3.804447000	-1.550991000	0.107102000
C	4.740675000	-0.510913000	-0.145822000
H	-1.511091000	0.195090000	-1.193991000
H	0.606866000	1.156600000	-1.361915000
H	-1.583488000	0.632256000	1.806057000
H	0.970968000	1.339058000	1.597283000
H	-2.024493000	-1.831029000	1.868970000
H	-1.979921000	-1.877479000	0.086312000
H	-1.825198000	2.711309000	-0.201749000
H	1.018762000	3.078474000	-0.107665000
H	1.728405000	-1.839826000	0.607167000
H	4.078021000	-2.592837000	0.071329000
H	-4.013444000	1.390691000	-0.467787000
H	6.564841000	-0.068344000	-0.870795000
O	6.375029000	-2.071078000	-0.842161000
H	6.968505000	-2.451824000	-0.182622000

H	-6.384655000	-0.859518000	0.566958000

Gibbs free energy = -1534.224309 Hartree

Number of imaginary frequencies: 0

M_uMP

	angstroms		
	X	Y	Z
P	-4.437689000	-0.733026000	-0.402074000
O	0.072015000	-0.476625000	1.040604000
O	-2.307793000	1.876323000	-0.245394000
O	0.069153000	2.813033000	-0.115153000
O	-3.551852000	-0.913059000	0.950783000
O	2.835810000	2.359832000	0.099218000
O	-5.836813000	-1.424121000	-0.050884000
O	-4.835494000	0.815831000	-0.385789000
O	-3.773722000	-1.282207000	-1.598231000
N	2.212283000	0.170212000	0.348977000
N	4.407125000	0.737421000	-0.187136000
N	6.083392000	-0.775761000	-0.515728000
C	-1.320957000	0.856383000	-0.312377000
C	0.123231000	1.428267000	-0.379916000
C	-1.294994000	-0.044554000	0.944539000
C	0.872199000	0.640430000	0.731065000
C	-2.167537000	-1.292934000	0.903238000
C	2.570509000	-1.172188000	0.343143000
C	3.142059000	1.170030000	0.086952000
C	3.822300000	-1.580100000	0.065676000
C	4.843025000	-0.599085000	-0.226980000
H	-1.529649000	0.249843000	-1.196046000

H	0.577232000	1.233776000	-1.355728000
H	-1.560741000	0.562013000	1.820409000
H	0.976674000	1.295214000	1.605797000
H	-1.981890000	-1.905382000	1.786124000
H	-1.964233000	-1.876647000	0.002730000
H	-1.853789000	2.720737000	-0.093308000
H	0.987959000	3.122282000	-0.051897000
H	1.766034000	-1.852896000	0.575265000
H	4.081729000	-2.626129000	0.058753000
H	-4.043068000	1.397128000	-0.376765000
O	6.403255000	-2.152168000	-0.525734000
H	7.338251000	-2.146067000	-0.753217000
H	-6.368414000	-0.935205000	0.588820000
H	5.084136000	1.461412000	-0.390010000

Gibbs free energy = -1534.230662 Hartree

Number of imaginary frequencies: 0

Structures of M_c and M_u substituted by: methyl, deoxyribose, ribose, ribose monophosphate optimized at B3LYP/6-311++G(d,p)/PCM from Table 8

M_c

	angstroms		
	X	Y	Z
O	-2.003901000	-1.732368000	0.029346000
O	3.394817000	0.305728000	0.253505000
N	0.211730000	-1.167125000	-0.017270000
N	2.416815000	-0.609930000	-0.171997000
C	-0.558533000	1.470379000	-0.026738000
C	-1.118421000	-0.874904000	0.002312000
C	0.772854000	1.184361000	-0.063625000
C	1.108062000	-0.194675000	-0.061859000
H	-0.928213000	2.487522000	-0.020282000
H	1.518886000	1.961643000	-0.086721000
H	3.931801000	0.476056000	-0.532224000
C	-2.926721000	0.808146000	0.039605000
H	-3.423361000	0.387915000	-0.834860000
H	-3.045700000	1.889581000	0.045064000
H	-3.377696000	0.384126000	0.936581000
N	-1.494986000	0.491740000	0.002278000

H	2.598608000	-1.536367000	0.200386000

Gibbs free energy = -509.479626 Hartree

Number of imaginary frequencies: 0

M_u

	angstroms		
	X	Y	Z
O	2.007332000	1.719608000	0.000437000
O	-3.404028000	-0.353414000	0.000383000
N	-0.191074000	1.123343000	-0.000482000
N	-2.434725000	0.689872000	-0.000050000
C	0.489953000	-1.473803000	-0.000173000
C	1.153665000	0.841309000	-0.000125000
C	-0.827854000	-1.185007000	0.000076000
C	-1.240723000	0.197022000	-0.000129000
H	0.847264000	-2.494743000	-0.000083000
H	-1.567936000	-1.967779000	0.000307000
H	-4.235291000	0.133569000	0.000315000
C	2.898848000	-0.869144000	0.000438000
H	3.392463000	-0.463484000	-0.882807000
H	2.978477000	-1.953717000	-0.008047000
H	3.388124000	-0.477605000	0.892580000
N	1.476442000	-0.509699000	-0.000807000
H	-0.427369000	2.107333000	0.000034000

Gibbs free energy = -509.483853 Hartree

Number of imaginary frequencies: 0

M_cdRib

	angstroms		
	X	Y	Z
O	-1.594835000	0.359411000	-0.756477000
O	-3.776063000	-1.774816000	0.357634000
O	-3.094126000	2.733071000	-0.954737000
O	1.044755000	-2.517361000	-0.941508000
N	0.514891000	-0.442529000	-0.111589000
N	2.771620000	-1.098678000	-0.487258000
N	4.503451000	0.329611000	-0.110688000
C	-3.067710000	-0.636482000	0.860273000
C	-1.602526000	-0.949973000	1.186199000
C	-2.966844000	0.385912000	-0.289782000
C	-0.917975000	-0.750791000	-0.167298000
C	-3.303999000	1.813647000	0.115277000
C	0.934240000	0.761844000	0.361129000
C	1.451272000	-1.424040000	-0.547093000
C	2.252074000	1.084048000	0.420669000
C	3.154681000	0.086372000	-0.041648000
H	-3.596506000	-0.221083000	1.722942000
H	-1.215711000	-0.232095000	1.914405000
H	-3.614514000	0.068362000	-1.111962000
H	-1.006474000	-1.635935000	-0.795115000

H	-2.715641000	2.104968000	0.994446000
H	-4.362540000	1.874095000	0.375814000
H	-3.898542000	-2.401770000	1.080191000
H	0.157393000	1.449214000	0.665341000
H	2.586945000	2.039806000	0.789235000
H	5.093677000	-0.495649000	-0.109684000
O	5.007538000	1.326052000	0.738377000
H	5.382262000	1.992012000	0.145672000
H	-2.169917000	2.660458000	-1.223251000
H	-1.467589000	-1.955277000	1.586037000

Gibbs free energy = -891.196924 Hartree

Number of imaginary frequencies: 0

M_udRib

	angstroms		
	X	Y	Z
O	-1.562988000	0.305225000	-0.747051000
O	-3.796620000	-1.718234000	0.483766000
O	-3.041034000	2.662775000	-1.159821000
O	1.050608000	-2.428658000	-1.158447000
N	0.521048000	-0.489258000	-0.024483000
N	2.730072000	-1.027554000	-0.551244000
N	4.541125000	0.257611000	-0.018917000
C	-3.073724000	-0.557920000	0.910653000
C	-1.618781000	-0.872407000	1.280214000
C	-2.941913000	0.374655000	-0.311663000
C	-0.908857000	-0.773918000	-0.071257000
C	-3.277168000	1.831375000	-0.024951000
C	0.977236000	0.689807000	0.542629000
C	1.408898000	-1.389964000	-0.621007000

C	2.278061000	1.029439000	0.593606000
C	3.257933000	0.132857000	0.027891000
H	-3.605856000	-0.071389000	1.733044000
H	-1.235824000	-0.120914000	1.974769000
H	-3.577751000	-0.001681000	-1.118469000
H	-1.011844000	-1.697183000	-0.639791000
H	-2.702707000	2.188355000	0.839056000
H	-4.339974000	1.918097000	0.209766000
H	-3.943157000	-2.285742000	1.249643000
H	0.205980000	1.337263000	0.933859000
H	2.594016000	1.956612000	1.042405000
O	4.949580000	1.470687000	0.596431000
H	5.907707000	1.446210000	0.496021000
H	3.377789000	-1.682243000	-0.971202000
H	-2.112380000	2.563508000	-1.403381000
H	-1.508187000	-1.854386000	1.741080000

Gibbs free energy = -891.201608 Hartree

Number of imaginary frequencies: 0

M_cRib

	angstroms		
	X	Y	Z
O	-1.484488000	0.797279000	-0.774934000
O	-3.727830000	-1.447766000	-0.444424000
O	-1.584689000	-2.448943000	0.757795000
O	-2.877080000	3.230352000	-0.094797000
O	0.898643000	-2.467490000	-0.302163000
N	0.587764000	-0.203915000	-0.286273000

N	2.758108000	-1.157089000	-0.142473000
N	4.620141000	0.146922000	-0.014596000
C	-3.051840000	-0.555548000	0.434782000
C	-1.612766000	-1.039449000	0.688165000
C	-2.855107000	0.791628000	-0.268263000
C	-0.859448000	-0.437761000	-0.518826000
C	-3.054218000	2.009966000	0.621500000
C	1.106728000	1.043517000	-0.149997000
C	1.425675000	-1.341657000	-0.248059000
C	2.446490000	1.237319000	-0.009347000
C	3.253459000	0.070597000	-0.036938000
H	-3.609909000	-0.451797000	1.370840000
H	-1.224678000	-0.586932000	1.608582000
H	-3.521878000	0.855663000	-1.131094000
H	-0.928717000	-1.106338000	-1.382413000
H	-2.381790000	1.966576000	1.486765000
H	-4.083321000	2.015714000	0.985876000
H	-3.398639000	-2.335295000	-0.233641000
H	-0.707429000	-2.724099000	0.417833000
H	0.395573000	1.854193000	-0.171191000
H	2.864481000	2.224713000	0.099018000
H	5.103799000	-0.685446000	0.305254000
O	5.179348000	1.314533000	0.520517000
H	5.674321000	1.712133000	-0.209290000
H	-1.968980000	3.264076000	-0.417201000

Gibbs free energy = -966.439081 Hartree

Number of imaginary frequencies: 0

M_uRib

	angstroms		
	X	Y	Z
O	-1.461611000	0.800905000	-0.769132000
O	-3.734703000	-1.419724000	-0.464648000
O	-1.613246000	-2.449584000	0.754239000
O	-2.837551000	3.246146000	-0.098954000
O	0.929426000	-2.485549000	-0.321670000
N	0.593746000	-0.218227000	-0.265207000
N	2.737118000	-1.122559000	-0.122346000
N	4.646124000	0.103569000	0.137962000
C	-3.054860000	-0.538917000	0.422922000
C	-1.622767000	-1.037729000	0.686324000
C	-2.837227000	0.807658000	-0.275824000
C	-0.853340000	-0.441318000	-0.511814000
C	-3.032956000	2.026671000	0.613807000
C	1.139816000	1.045719000	-0.119758000
C	1.397570000	-1.346634000	-0.242837000
C	2.462856000	1.253580000	0.026217000
C	3.361696000	0.124867000	0.022994000
H	-3.619088000	-0.430554000	1.354675000
H	-1.237246000	-0.593727000	1.611532000
H	-3.494922000	0.879309000	-1.144991000
H	-0.926697000	-1.106040000	-1.378438000
H	-2.369369000	1.975537000	1.485423000
H	-4.065398000	2.041532000	0.968375000
H	-3.428284000	-2.313757000	-0.248635000
H	-0.744485000	-2.750284000	0.428226000
H	0.423690000	1.851047000	-0.137697000
H	2.856130000	2.250389000	0.138624000
O	5.155893000	1.420386000	0.285152000

H	6.105241000	1.272732000	0.360889000
H	3.317118000	-1.952009000	-0.090695000
H	-1.925999000	3.272321000	-0.412235000

Gibbs free energy = -966.440546 Hartree

Number of imaginary frequencies: 0

M_cMP

	angstroms		
	X	Y	Z
P	-4.582056000	-0.712985000	-0.308341000
O	0.070293000	-0.484387000	0.994399000
O	-2.229086000	1.812928000	-0.555178000
O	0.127876000	2.791704000	-0.163423000
O	-3.556660000	-0.846662000	0.928876000
O	2.865763000	2.340256000	0.281735000
O	-5.997233000	-1.117939000	0.314156000
O	-4.734405000	0.856828000	-0.550428000
O	-4.235977000	-1.548420000	-1.481597000
N	2.250489000	0.147469000	0.386946000
N	4.499750000	0.772694000	-0.038456000
N	6.129935000	-0.794999000	-0.295525000
C	-1.221174000	0.813951000	-0.468223000
C	0.211824000	1.408705000	-0.444372000
C	-1.280700000	-0.031972000	0.824248000
C	0.898433000	0.621880000	0.707027000
C	-2.178015000	-1.262117000	0.802922000
C	2.564851000	-1.169151000	0.277851000
C	3.228292000	1.155319000	0.210455000
C	3.842427000	-1.558335000	0.017240000

C	4.799837000	-0.518038000	-0.121435000
H	-1.336809000	0.165940000	-1.338234000
H	0.727270000	1.233391000	-1.391501000
H	-1.570539000	0.610380000	1.665976000
H	0.979127000	1.277933000	1.580161000
H	-1.967295000	-1.887972000	1.669138000
H	-2.039731000	-1.846155000	-0.108799000
H	-1.867049000	2.642861000	-0.205387000
H	1.040479000	3.077953000	0.017196000
H	1.749873000	-1.864578000	0.414323000
H	4.107470000	-2.599038000	-0.072172000
H	-3.866217000	1.335413000	-0.572854000
H	6.680153000	-0.057287000	-0.722552000
O	6.450050000	-2.054387000	-0.819554000
H	6.974165000	-2.485759000	-0.130514000
H	-6.320897000	-0.545272000	1.023416000

Gibbs free energy = -1534.257552 Hartree

Number of imaginary frequencies: 0

M_uMP

angstroms

	X	Y	Z
P	-4.565823000	-0.741793000	-0.304308000
O	0.085412000	-0.440775000	1.009510000
O	-2.234112000	1.809747000	-0.586804000
O	0.088344000	2.833411000	-0.108449000
O	-3.536942000	-0.833249000	0.933911000
O	2.930656000	2.355322000	0.255323000
O	-5.976961000	-1.137292000	0.333450000
O	-4.727652000	0.819429000	-0.591586000

O	-4.218513000	-1.608937000	-1.453871000
N	2.255290000	0.172368000	0.365681000
N	4.476606000	0.713025000	-0.077176000
N	6.122243000	-0.822724000	-0.459775000
C	-1.205912000	0.835417000	-0.467994000
C	0.212681000	1.459793000	-0.425297000
C	-1.267881000	0.001239000	0.831215000
C	0.913328000	0.662171000	0.711147000
C	-2.154295000	-1.237161000	0.816222000
C	2.570844000	-1.171178000	0.260927000
C	3.213904000	1.159590000	0.185325000
C	3.820153000	-1.595761000	-0.011258000
C	4.874544000	-0.626940000	-0.197517000
H	-1.289474000	0.174835000	-1.332215000
H	0.734583000	1.324473000	-1.374978000
H	-1.566771000	0.647211000	1.666945000
H	1.009275000	1.314505000	1.585792000
H	-1.940162000	-1.853786000	1.688274000
H	-2.007102000	-1.827733000	-0.089890000
H	-1.904501000	2.648614000	-0.227045000
H	0.986856000	3.172447000	0.026963000
H	1.742786000	-1.845945000	0.414926000
H	4.041838000	-2.647269000	-0.088957000
H	-3.863298000	1.304722000	-0.615289000
O	6.400264000	-2.211337000	-0.560843000
H	7.343586000	-2.224278000	-0.757657000
H	-6.302883000	-0.544721000	1.025094000
H	5.175465000	1.431579000	-0.220202000

Gibbs free energy = -1534.259086 Hartree

Number of imaginary frequencies: 0

Structures of reactants, products, Transition States and Transition Product M_{TP} optimized at B3LYP/6-311++G(d,p) from Figures 10-11 and Table 9

Reactant M_u with a water molecule

	angstroms		
	X	Y	Z
O	-1.706187000	1.968974000	-0.002916000
O	2.721243000	-1.773842000	0.013358000
N	0.193393000	0.703666000	-0.012397000
N	2.166380000	-0.466967000	-0.004114000
C	-1.302085000	-1.539143000	0.004941000
C	-1.171701000	0.876788000	-0.004839000
C	0.037536000	-1.693840000	0.004367000
C	0.872921000	-0.516639000	-0.003920000
H	-1.971503000	-2.390143000	0.008602000
H	0.493349000	-2.669741000	0.006625000
H	3.663785000	-1.603202000	-0.074280000
C	-3.376400000	-0.172772000	0.008149000
H	-3.705600000	0.360890000	0.900913000
H	-3.816804000	-1.168773000	-0.004786000
H	-3.709844000	0.385835000	-0.867241000
N	-1.919444000	-0.308992000	0.001068000
H	2.855999000	1.381596000	-0.004721000

O	2.600428000	2.321507000	-0.073860000
H	3.069514000	2.790854000	0.621805000
H	0.753307000	1.559263000	-0.023661000

Gibbs free energy = -585.921216 Hartree

Number of imaginary frequencies: 0

TS (Transition State of $M_u \rightarrow M_c$)

	angstroms		
	X	Y	Z
O	-1.817087000	1.868360000	-0.022675000
O	2.956481000	-1.415986000	0.131067000
N	0.184381000	0.761932000	-0.076388000
N	2.198373000	-0.256849000	-0.111142000
C	-1.170711000	-1.593324000	0.035159000
C	-1.179948000	0.832431000	-0.035364000
C	0.184668000	-1.653629000	0.016593000
C	0.883973000	-0.409109000	-0.058184000
H	-1.777114000	-2.489964000	0.080200000
H	0.718059000	-2.589083000	0.051800000
H	3.599897000	-1.432666000	-0.588390000
C	-3.320045000	-0.373893000	0.034665000
H	-3.662534000	0.128822000	0.940284000
H	-3.701353000	-1.394147000	0.012697000
H	-3.697800000	0.179873000	-0.824971000
N	-1.857442000	-0.417960000	-0.001499000
H	2.524170000	0.903332000	0.028054000
O	2.179984000	2.156455000	0.005574000
H	2.390035000	2.693869000	0.775524000
H	0.986813000	1.704629000	-0.020933000

Gibbs free energy = -585.892089 Hartree

Number of imaginary frequencies: 1

Product M_c with a water molecule

	angstroms		
	X	Y	Z
O	1.724163000	1.922834000	-0.143382000
O	-2.824356000	-1.630855000	0.183923000
N	-0.224533000	0.718908000	-0.164855000
N	-2.155739000	-0.486985000	-0.292315000
C	1.294040000	-1.554555000	0.099461000
C	1.134571000	0.857148000	-0.101406000
C	-0.057714000	-1.686785000	0.019950000
C	-0.794174000	-0.474766000	-0.126832000
H	1.947455000	-2.410668000	0.218835000
H	-0.542503000	-2.646746000	0.085509000
H	-3.290039000	-1.974398000	-0.588541000
C	3.352387000	-0.199497000	0.119976000
H	3.730898000	0.294418000	-0.775457000
H	3.801069000	-1.187774000	0.217274000
H	3.616132000	0.415358000	0.981224000
N	1.899265000	-0.345012000	0.033935000
H	-2.637876000	0.381264000	-0.044457000
O	-2.476820000	2.329956000	0.310482000
H	-2.681012000	3.160353000	-0.128099000
H	-1.535619000	2.135070000	0.121280000

Gibbs free energy = -585.913366 Hartree

Number of imaginary frequencies: 0

Reactant M_u-m with a water molecule

angstroms

	X	Y	Z
O	2.193254000	1.897394000	-0.003945000
O	-2.353356000	1.142567000	0.018765000
N	0.175176000	0.843156000	0.005897000
N	-1.934222000	-0.205774000	0.014579000
C	1.386050000	-1.558954000	0.000831000
C	1.559201000	0.860086000	-0.000134000
C	0.041278000	-1.549808000	0.006161000
C	-0.644882000	-0.280208000	0.009110000
H	1.953123000	-2.480842000	-0.003030000
H	-0.533276000	-2.463236000	0.005719000
H	-3.322121000	1.057430000	-0.013836000
C	3.612196000	-0.498374000	-0.007678000
H	3.955132000	-1.019846000	-0.904856000
H	3.962250000	-1.031828000	0.879586000
H	4.015595000	0.510609000	-0.002772000
N	2.152516000	-0.402953000	-0.000694000
H	-0.275665000	1.748833000	0.002085000
O	-4.719872000	-0.329494000	-0.104925000
H	-3.908301000	-0.861735000	-0.047385000
H	-5.284292000	-0.620579000	0.617121000

Gibbs free energy = -585.925561 Hartree

Number of imaginary frequencies: 0

TS1 (Transition State of M_u-m→M_{TP})

angstroms

X Y Z

O	2.151685000	1.882731000	-0.013305000
O	-2.502551000	1.127297000	0.028216000
N	0.116938000	0.846660000	0.012781000
N	-1.970792000	-0.133028000	0.034629000
C	1.331927000	-1.559921000	0.004807000
C	1.501622000	0.858856000	-0.000389000
C	-0.016424000	-1.550097000	0.014832000
C	-0.686199000	-0.279399000	0.020374000
H	1.894402000	-2.483899000	-0.002229000
H	-0.588488000	-2.465108000	0.014962000
H	-0.353879000	1.745087000	0.003735000
C	3.557397000	-0.450422000	-0.019184000
H	3.963299000	0.112072000	0.821769000
H	3.939360000	-0.013376000	-0.943149000
H	3.873292000	-1.490053000	0.050443000
N	2.093121000	-0.406512000	0.003427000
H	-2.980395000	-0.748165000	-0.000088000
O	-4.285847000	-0.392231000	-0.139636000
H	-3.596751000	0.686341000	-0.039107000
H	-4.861948000	-0.549223000	0.612973000

Gibbs free energy = -585.887903 Hartree

Number of imaginary frequencies: 1

M_{TP} (Transition Product)

	X	Y	Z
	angstroms		
O	2.191766000	1.902211000	-0.016061000
O	-2.451890000	1.120176000	0.032889000
N	0.171312000	0.851252000	0.010674000
N	-1.914487000	-0.081126000	0.035483000

C	1.384967000	-1.556012000	0.007165000
C	1.554450000	0.870376000	-0.004422000
C	0.035589000	-1.547567000	0.021142000
C	-0.620970000	-0.276927000	0.022800000
H	1.951100000	-2.478047000	0.003566000
H	-0.533444000	-2.465459000	0.028205000
H	-0.323105000	1.739255000	0.007979000
C	3.607406000	-0.493716000	-0.020629000
H	4.009624000	0.515616000	-0.024997000
H	3.943599000	-1.020442000	-0.917005000
H	3.963026000	-1.020751000	0.867998000
N	2.146461000	-0.399573000	-0.004428000
H	-2.567870000	-0.869843000	0.041957000
O	-4.672866000	-0.409448000	-0.123644000
H	-4.162400000	0.428170000	-0.086618000
H	-5.408261000	-0.302811000	0.485007000

Gibbs free energy = -585.913537 Hartree

Number of imaginary frequencies: 0

TS2 (Transition State of $M_{TP} \rightarrow M_{c}$-m)

	angstroms		
	X	Y	Z
O	1.991689000	-1.942858000	0.080972000
O	-2.245503000	-1.058971000	-0.226070000
N	0.045578000	-0.740370000	-0.091322000
N	-1.884314000	0.264299000	-0.270334000
C	1.495682000	1.555779000	0.000130000
C	1.401014000	-0.884938000	0.015955000
C	0.147073000	1.681485000	-0.100928000

C	-0.576243000	0.457582000	-0.151266000
H	2.143541000	2.422033000	0.046491000
H	-0.335793000	2.646104000	-0.141209000
H	-1.012867000	-1.405558000	-0.129885000
C	3.581442000	0.239202000	0.159739000
H	3.980140000	-0.333255000	-0.678289000
H	3.855800000	-0.272736000	1.082959000
H	4.006284000	1.242174000	0.155064000
N	2.124710000	0.343307000	0.050877000
H	-2.635057000	0.935221000	-0.146542000
O	-4.593487000	0.237802000	0.416621000
H	-4.077664000	-0.577647000	0.283381000
H	-5.441607000	0.090569000	-0.010493000

Gibbs free energy = -585.898995 Hartree

Number of imaginary frequencies: 1

Final product M_c-m with a water molecule

	angstroms		
	X	Y	Z
O	-2.146719000	-1.857794000	0.255669000
O	2.415128000	-1.187319000	-0.405102000
N	-0.109878000	-0.882682000	-0.146358000
N	1.890125000	0.103860000	-0.604036000
C	-1.378804000	1.557933000	-0.088529000
C	-1.462772000	-0.868764000	0.071901000
C	-0.037707000	1.536185000	-0.302323000
C	0.559717000	0.238703000	-0.328972000
H	-1.939513000	2.484032000	-0.050441000
H	0.530033000	2.441801000	-0.457466000
H	1.601537000	-1.713343000	-0.225320000

C	-3.538649000	0.439560000	0.316189000
H	-3.772893000	-0.005450000	1.284043000
H	-4.047730000	-0.140888000	-0.453896000
H	-3.883978000	1.472783000	0.289894000
N	-2.093780000	0.416374000	0.087518000
H	2.549316000	0.779519000	-0.224320000
O	4.390421000	0.500355000	0.692045000
H	4.166964000	-0.437326000	0.607068000
H	4.869647000	0.592370000	1.520073000

Gibbs free energy = -585.910837 Hartree

Number of imaginary frequencies: 0

Reactant - cytosine with a water molecule

	angstroms		
	X	Y	Z
O	-0.859790000	-2.076876000	-0.049177000
N	-1.683838000	0.047937000	0.012109000
N	0.665783000	-0.369420000	-0.040921000
N	2.163524000	1.364588000	-0.059878000
C	-3.037432000	-0.505111000	0.032488000
C	-1.452635000	1.382876000	0.028306000
C	-0.598858000	-0.886125000	-0.028635000
C	-0.192560000	1.889043000	0.007839000
C	0.881214000	0.939456000	-0.028070000
H	-3.208402000	-1.110384000	-0.858491000
H	-2.327738000	2.021218000	0.057740000
H	-0.018268000	2.955422000	0.017352000
H	2.382848000	2.341402000	0.024816000
H	-3.753788000	0.315643000	0.066234000
H	-3.166688000	-1.146272000	0.905138000

H	2.329412000	-1.327487000	0.071188000
O	3.308540000	-1.288094000	0.134718000
H	2.909086000	0.672643000	-0.038114000
H	3.646888000	-2.024983000	-0.380933000

Gibbs free energy = -510.734959 Hartree

Number of imaginary frequencies: 0

TS (Transition State of C→C_u)

angstroms

	X	Y	Z
O	0.994136000	-2.046922000	0.011023000
N	-0.644537000	-0.449016000	-0.002056000
N	-2.333106000	1.070752000	0.007601000
C	1.280298000	1.461434000	0.002656000
C	0.654580000	-0.880584000	0.002700000
C	-0.012852000	1.869309000	0.009398000
C	-1.031967000	0.859380000	0.005293000
H	2.099565000	2.170201000	0.000769000
H	-0.266970000	2.918989000	0.012945000
C	3.036407000	-0.278693000	-0.006993000
H	3.251790000	-0.876088000	0.879585000
H	3.671097000	0.606585000	-0.018453000
H	3.239504000	-0.889857000	-0.886895000
N	1.635778000	0.147282000	-0.001526000
H	-2.929294000	0.028608000	-0.001365000
O	-2.942754000	-1.281161000	-0.095313000
H	-3.375922000	-1.785270000	0.599139000
H	-1.608173000	-1.142294000	-0.014941000
H	-2.658385000	2.025586000	-0.002926000

Gibbs free energy = -510.709749 Hartree

Number of imaginary frequencies: 1

Product - C_u with a water molecule

	angstroms		
	X	Y	Z
O	-0.904148000	-2.087039000	0.018081000
N	-1.695437000	0.058456000	-0.002191000
N	0.607739000	-0.373120000	0.012490000
N	2.227526000	1.267003000	0.022607000
C	-3.068628000	-0.447921000	-0.009531000
C	-1.413528000	1.402674000	-0.008936000
C	-0.666749000	-0.895444000	0.009851000
C	-0.155471000	1.889170000	-0.002365000
C	0.970877000	0.975210000	0.011476000
H	-3.241270000	-1.064518000	-0.892527000
H	-2.276958000	2.056159000	-0.019662000
H	0.022628000	2.954684000	-0.007385000
H	2.375111000	2.271814000	0.012719000
H	-3.750864000	0.401001000	-0.017242000
H	-3.253007000	-1.057967000	0.875613000
H	1.372964000	-1.054262000	0.008996000
O	3.342547000	-1.270604000	-0.106628000
H	3.295291000	-0.290484000	-0.044071000
H	3.971114000	-1.563791000	0.558619000

Gibbs free energy = -510.729274 Hartree

Number of imaginary frequencies: 0

Reactant - uracil with a water molecule

angstroms

	X	Y	Z
O	0.902544000	-2.091422000	0.016420000
O	-2.162659000	1.305980000	0.015299000
N	-0.613770000	-0.374980000	0.010974000
N	1.686172000	0.058728000	-0.003392000
C	0.664720000	-0.901094000	0.008323000
C	-0.979787000	0.974957000	0.009583000
C	1.402341000	1.400477000	-0.005934000
C	0.143194000	1.889553000	0.001297000
H	-1.381549000	-1.052788000	0.013117000
C	3.062180000	-0.444121000	-0.009541000
H	2.266637000	2.052997000	-0.014286000
H	3.741965000	0.406326000	-0.024349000
H	3.232230000	-1.066242000	-0.888815000
H	3.247706000	-1.047961000	0.879425000
H	-0.053682000	2.950581000	-0.001493000
O	-3.355818000	-1.193866000	-0.093356000
H	-4.063162000	-1.492417000	0.484636000
H	-3.325382000	-0.220895000	-0.030583000

Gibbs free energy = -530.635459 Hartree

Number of imaginary frequencies: 0

TS (Transition State of U→U_c)

angstroms

	X	Y	Z
O	1.002815000	-2.049119000	0.008370000
O	-2.287099000	1.104613000	0.001193000
N	-0.653933000	-0.466401000	-0.003268000
N	1.625586000	0.149995000	-0.001559000

C	0.652653000	-0.884462000	0.000819000
C	-1.033576000	0.832638000	0.003023000
C	1.262610000	1.461603000	0.005535000
C	-0.035695000	1.857421000	0.011417000
H	-1.774407000	-1.175488000	-0.027562000
C	3.029616000	-0.265735000	-0.005437000
H	2.077907000	2.174900000	0.005870000
H	3.658512000	0.623578000	-0.016155000
H	3.236535000	-0.876058000	-0.884965000
H	3.246855000	-0.862376000	0.881155000
H	-0.321041000	2.897937000	0.015347000
O	-2.957935000	-1.180817000	-0.094310000
H	-3.378695000	-1.594011000	0.666872000
H	-2.863130000	0.020148000	-0.020939000

Gibbs free energy = -530.607482 Hartree

Number of imaginary frequencies: 1

Product - U_c with a water molecule

	angstroms		
	X	Y	Z
O	0.929612000	-2.076073000	0.017435000
O	-2.159105000	1.299537000	0.014349000
N	-0.654035000	-0.418429000	0.007938000
N	1.677527000	0.078976000	-0.004634000
C	0.634266000	-0.895196000	0.007399000
C	-0.901308000	0.873585000	0.009525000
C	1.399337000	1.406554000	-0.002846000
C	0.120281000	1.866477000	0.006583000
H	-2.369989000	-1.385973000	-0.052947000
C	3.051934000	-0.424257000	-0.012203000

H	2.252476000	2.073962000	-0.009624000
H	3.737641000	0.422242000	-0.029581000
H	3.213692000	-1.051943000	-0.888984000
H	3.232391000	-1.031037000	0.875753000
H	-0.118095000	2.918683000	0.006919000
O	-3.319058000	-1.146147000	-0.099777000
H	-3.779748000	-1.672400000	0.560393000
H	-2.771453000	0.521117000	0.008137000

Gibbs free energy = -530.620539 Hartree

Number of imaginary frequencies: 0

Structures of reactants, products Transition States and Transition Product M_{TP} optimized at B3LYP/6-311++G(d,p)/PCM from Figures 10-11 and Table 9

Reactant M_u with a water molecule

	angstroms		
	X	Y	Z
O	-1.683823000	1.992699000	-0.003092000
O	2.716915000	-1.780989000	0.000495000
N	0.187617000	0.694982000	-0.002953000
N	2.151278000	-0.478775000	-0.003572000
C	-1.330746000	-1.523354000	0.005637000
C	-1.172881000	0.879240000	-0.001410000
C	0.008258000	-1.695511000	0.005010000
C	0.856872000	-0.531762000	-0.000227000
H	-2.010736000	-2.364505000	0.008892000

H	0.442444000	-2.681286000	0.007596000
H	3.664167000	-1.603153000	-0.008146000
C	-3.393725000	-0.146251000	0.002606000
H	-3.722204000	0.396748000	0.888984000
H	-3.831605000	-1.141830000	0.005784000
H	-3.723126000	0.391421000	-0.886682000
N	-1.933097000	-0.285209000	0.002254000
H	2.858792000	1.329513000	-0.009570000
O	2.691486000	2.291146000	-0.086992000
H	3.121605000	2.697358000	0.673402000
H	0.756775000	1.541739000	-0.003354000

Gibbs free energy = -585.938395 Hartree

Number of imaginary frequencies: 0

TS (Transition State of $M_u \rightarrow M_c$)

	angstroms		
	X	Y	Z
O	-1.782762000	1.894836000	-0.031225000
O	2.973243000	-1.418747000	0.149123000
N	0.180921000	0.734601000	-0.068033000
N	2.192355000	-0.295035000	-0.118625000
C	-1.196782000	-1.580286000	0.034596000
C	-1.187021000	0.828100000	-0.032383000
C	0.160230000	-1.662074000	0.010195000
C	0.876649000	-0.439108000	-0.055839000
H	-1.814514000	-2.467389000	0.075937000
H	0.671630000	-2.609913000	0.032489000
H	3.483370000	-1.572978000	-0.659192000
C	-3.338056000	-0.347387000	0.037625000

H	-3.675405000	0.172601000	0.933922000
H	-3.716169000	-1.366720000	0.041254000
H	-3.709344000	0.178417000	-0.841261000
N	-1.869700000	-0.395910000	0.006848000
H	2.553319000	0.744601000	0.008985000
O	2.242559000	2.210013000	-0.000449000
H	2.479512000	2.718752000	0.781963000
H	0.848135000	1.612748000	-0.020196000

Gibbs free energy = -585.911979 Hartree

Number of imaginary frequencies: 1

Product M_c with a water molecule

	angstroms		
	X	Y	Z
O	1.691969000	1.927838000	-0.105850000
O	-2.836168000	-1.658088000	0.197940000
N	-0.238113000	0.704227000	-0.132591000
N	-2.165700000	-0.512256000	-0.253631000
C	1.294029000	-1.559656000	0.072043000
C	1.117325000	0.839050000	-0.077746000
C	-0.058921000	-1.700964000	0.007785000
C	-0.805172000	-0.499122000	-0.106413000
H	1.955706000	-2.411260000	0.160969000
H	-0.529448000	-2.669587000	0.044163000
H	-3.248490000	-2.034084000	-0.591930000
C	3.347677000	-0.205805000	0.095789000
H	3.715589000	0.304180000	-0.794158000
H	3.787716000	-1.198851000	0.157945000
H	3.626211000	0.375447000	0.974806000
N	1.888944000	-0.342959000	0.025971000

H	-2.654571000	0.348232000	-0.008164000
O	-2.395472000	2.470421000	0.281094000
H	-2.592312000	3.082996000	-0.435783000
H	-1.508581000	2.097454000	0.079694000

Gibbs free energy = -585.935100 Hartree

Number of imaginary frequencies: 0

Reactant M_u-m with a water molecule

	angstroms		
	X	Y	Z
O	-2.192381000	-1.890783000	-0.002277000
O	2.360907000	-1.116921000	0.013985000
N	-0.175250000	-0.828694000	0.003876000
N	1.928619000	0.236714000	0.010785000
C	-1.407833000	1.562789000	0.000787000
C	-1.556522000	-0.844317000	-0.000007000
C	-0.061712000	1.562553000	0.004541000
C	0.636022000	0.300888000	0.006479000
H	-1.980232000	2.479841000	-0.000931000
H	0.499848000	2.484192000	0.005956000
H	3.329731000	-1.016192000	0.001454000
C	-3.627646000	0.442586000	-0.005247000
H	-4.019333000	-0.050117000	-0.895550000
H	-3.939673000	1.484329000	-0.003229000
H	-4.024328000	-0.055033000	0.880052000
N	-2.161310000	0.399023000	-0.001052000
H	0.278427000	-1.732985000	0.003854000
O	4.763770000	0.288197000	-0.106492000
H	4.008764000	0.889490000	-0.002810000

H	5.350152000	0.476232000	0.634902000

Gibbs free energy = -585.942375 Hartree

Number of imaginary frequencies: 0

TS1 (Transition State of M_u-m→M_{TP})

	angstroms		
	X	Y	Z
O	2.172774000	-1.874682000	-0.002882000
O	-2.507683000	-1.172792000	-0.002978000
N	0.132897000	-0.856822000	-0.001070000
N	-1.965806000	0.099574000	-0.002319000
C	1.320253000	1.554049000	0.001155000
C	1.521503000	-0.845315000	-0.001687000
C	-0.030807000	1.528485000	0.001215000
C	-0.675756000	0.252254000	-0.000414000
H	1.867618000	2.486312000	0.002448000
H	-0.611729000	2.437309000	0.002605000
H	-0.304780000	-1.771109000	-0.001129000
C	3.562106000	0.489412000	-0.001074000
H	3.959709000	0.001411000	-0.890510000
H	3.960379000	-0.001256000	0.886583000
H	3.852628000	1.536820000	0.000405000
N	2.093810000	0.416524000	-0.000613000
H	-2.860674000	0.744067000	-0.003238000
O	-4.353970000	0.454302000	0.096364000
H	-3.497505000	-0.838895000	0.003565000
H	-4.864712000	0.662474000	-0.691913000

Gibbs free energy = -585.907928 Hartree

Number of imaginary frequencies: 1

M_{TP} (Transition Product)

	angstroms		
	X	Y	Z
O	-1.955978000	-2.035225000	0.001643000
O	2.524030000	-0.582683000	-0.022408000
N	-0.112479000	-0.693538000	-0.007180000
N	1.808461000	0.543282000	-0.017281000
C	-1.679153000	1.493670000	0.004220000
C	-1.478283000	-0.912280000	0.000392000
C	-0.342522000	1.690705000	-0.003342000
C	0.498136000	0.536480000	-0.009502000
H	-2.375852000	2.319844000	0.008985000
H	0.078111000	2.684944000	-0.004881000
H	0.502739000	-1.501445000	-0.012444000
C	-3.712084000	0.070107000	0.015051000
H	-4.021942000	-0.479806000	0.903618000
H	-4.033279000	-0.474315000	-0.872893000
H	-4.169550000	1.056462000	0.021046000
N	-2.252971000	0.238122000	0.006397000
H	2.303512000	1.431492000	-0.019944000
O	5.205689000	-0.182974000	-0.071579000
H	4.228464000	-0.332741000	-0.050072000
H	5.480228000	-0.184545000	0.850869000

Gibbs free energy = -585.939163 Hartree

Number of imaginary frequencies: 0

TS2 (Transition State of $M_{TP} \rightarrow M_c$-m)

	angstroms		
	X	Y	Z
O	-1.404623000	-2.104874000	-0.302013000
O	2.382407000	-0.075994000	-0.765653000
N	0.131675000	-0.409326000	-0.327427000
N	1.735643000	1.081123000	-0.368603000
C	-1.822266000	1.351338000	0.312058000
C	-1.124433000	-0.921222000	-0.165368000
C	-0.569320000	1.852911000	0.144261000
C	0.424501000	0.898087000	-0.180484000
H	-2.658553000	1.989360000	0.563724000
H	-0.355199000	2.904459000	0.256801000
H	1.299676000	-0.735710000	-0.650312000
C	-3.480941000	-0.476841000	0.367638000
H	-3.496868000	-1.224280000	1.160467000
H	-3.845911000	-0.929730000	-0.554135000
H	-4.120432000	0.358839000	0.641760000
N	-2.112879000	0.022456000	0.176663000
H	2.174256000	1.962789000	-0.593799000
O	4.546984000	-0.771502000	0.826751000
H	3.760553000	-0.529047000	0.292503000
H	4.198015000	-1.263129000	1.577255000

Gibbs free energy = -585.923804 Hartree

Number of imaginary frequencies: 1

Final product M_c-m with a water molecule

	angstroms		
	X	Y	Z
O	-1.628932000	-2.095680000	-0.215302000
O	2.511051000	-0.080213000	-0.805867000

N	0.052028000	-0.546505000	-0.292282000
N	1.739430000	0.990845000	-0.307381000
C	-1.812582000	1.385859000	0.268410000
C	-1.249864000	-0.930350000	-0.121104000
C	-0.519321000	1.771034000	0.099961000
C	0.398030000	0.723828000	-0.181209000
H	-2.599414000	2.095771000	0.485923000
H	-0.219967000	2.805143000	0.181708000
H	1.847418000	-0.807341000	-0.860798000
C	-3.588208000	-0.318478000	0.361103000
H	-3.662688000	-1.031386000	1.181976000
H	-3.966344000	-0.787042000	-0.547415000
H	-4.177793000	0.566483000	0.590056000
N	-2.189256000	0.082964000	0.173515000
H	2.002148000	1.846381000	-0.783089000
O	4.825455000	-0.658915000	0.801065000
H	4.016406000	-0.474449000	0.293117000
H	4.515904000	-1.017578000	1.639430000

Gibbs free energy = -585.934442 Hartree

Number of imaginary frequencies: 0

Reactant - cytosine with a water molecule

	angstroms		
	X	Y	Z
O	-0.762888000	-2.085186000	0.008472000
N	-1.666625000	0.009135000	-0.001835000
N	0.688266000	-0.320957000	0.005215000
N	2.126654000	1.466668000	0.004155000
C	-3.011076000	-0.575542000	-0.002320000
C	-1.483721000	1.355191000	-0.007345000

C	-0.558865000	-0.868727000	0.004401000
C	-0.243145000	1.907078000	-0.007089000
C	0.862151000	1.003012000	-0.000181000
H	-3.148171000	-1.198999000	-0.885851000
H	-2.381774000	1.958809000	-0.012066000
H	-0.107843000	2.978482000	-0.011421000
H	2.319429000	2.454440000	-0.013415000
H	-3.741015000	0.231451000	-0.007990000
H	-3.152571000	-1.190456000	0.886495000
H	2.301580000	-1.241606000	0.022687000
O	3.272918000	-1.400734000	0.084121000
H	2.897665000	0.810504000	-0.006419000
H	3.502322000	-1.925260000	-0.690296000

Gibbs free energy = -510.756792 Hartree

Number of imaginary frequencies: 0

TS (Transition State of C→C_u)

	angstroms		
	X	Y	Z
O	0.969360000	-2.067815000	-0.005952000
N	-0.619242000	-0.430335000	-0.013793000
N	-2.306572000	1.095493000	-0.010717000
C	1.308191000	1.441396000	0.010947000
C	0.678960000	-0.881337000	-0.005488000
C	0.020146000	1.868081000	0.008892000
C	-1.009720000	0.882534000	-0.004425000
H	2.134468000	2.139496000	0.019729000
H	-0.219463000	2.920341000	0.015559000
C	3.059668000	-0.307286000	0.005323000

H	3.269836000	-0.891859000	0.900698000
H	3.689647000	0.578672000	-0.008955000
H	3.264323000	-0.915848000	-0.874917000
N	1.653617000	0.120216000	0.002394000
H	-2.915426000	0.142820000	-0.000747000
O	-3.096228000	-1.276413000	-0.075764000
H	-3.563155000	-1.674093000	0.666218000
H	-1.438730000	-1.110083000	-0.003947000
H	-2.644647000	2.046437000	0.003405000

Gibbs free energy = -510.731679 Hartree

Number of imaginary frequencies: 1

Product - C_u with a water molecule

	angstroms		
	X	Y	Z
O	-0.917659000	-2.098437000	-0.003485000
N	-1.709418000	0.051369000	0.002496000
N	0.584452000	-0.382957000	-0.002991000
N	2.220462000	1.237219000	-0.005616000
C	-3.093552000	-0.436623000	0.003239000
C	-1.420732000	1.397013000	0.006115000
C	-0.692692000	-0.894631000	-0.001482000
C	-0.160170000	1.879628000	0.004977000
C	0.956996000	0.962971000	-0.000958000
H	-3.280879000	-1.040462000	-0.884752000
H	-2.281229000	2.052502000	0.009626000
H	0.021422000	2.944370000	0.007481000
H	2.380266000	2.240523000	-0.004201000
H	-3.760551000	0.422242000	0.004110000
H	-3.279814000	-1.041519000	0.890773000

H	1.339840000	-1.067773000	-0.003720000
O	3.469147000	-1.225991000	-0.087343000
H	3.265255000	-0.259815000	-0.023122000
H	3.976207000	-1.444209000	0.701865000

Gibbs free energy = -510.746661 Hartree

Number of imaginary frequencies: 0

Reactant - uracil with a water molecule

	angstroms		
	X	Y	Z
O	0.888982000	-2.099260000	0.001152000
O	-2.156398000	1.301796000	0.000986000
N	-0.603430000	-0.369015000	0.000920000
N	1.693103000	0.047678000	0.001112000
C	0.672660000	-0.896774000	0.001037000
C	-0.962162000	0.978732000	0.001836000
C	1.415399000	1.389044000	0.002337000
C	0.155596000	1.885158000	0.002958000
H	-1.370202000	-1.043869000	0.003158000
C	3.075242000	-0.451096000	0.000729000
H	2.280748000	2.038252000	0.002542000
H	3.748695000	0.402371000	-0.000637000
H	3.252838000	-1.057941000	-0.886770000
H	3.254131000	-1.056157000	0.889217000
H	-0.026705000	2.948838000	0.003697000
O	-3.434667000	-1.174724000	-0.091719000
H	-4.001178000	-1.423171000	0.646757000
H	-3.289784000	-0.211842000	-0.008934000

Gibbs free energy = -530.652964 Hartree

Number of imaginary frequencies: 0

TS (Transition State of U→U_c)

angstroms

	X	Y	Z
O	0.982981000	-2.055684000	-0.013425000
O	-2.290662000	1.105568000	-0.013702000
N	-0.652803000	-0.458997000	-0.016095000
N	1.622931000	0.141576000	0.004520000
C	0.650959000	-0.875355000	-0.008780000
C	-1.026749000	0.841865000	-0.007224000
C	1.266765000	1.454225000	0.013361000
C	-0.032482000	1.857363000	0.008587000
H	-1.756083000	-1.157713000	-0.014765000
C	3.032941000	-0.269423000	0.009427000
H	2.083699000	2.163593000	0.024416000
H	3.655110000	0.622516000	0.010608000
H	3.248486000	-0.865962000	-0.876610000
H	3.243418000	-0.865090000	0.897402000
H	-0.303614000	2.901698000	0.015456000
O	-2.957206000	-1.188047000	-0.070947000
H	-3.344037000	-1.559654000	0.731179000
H	-2.847382000	0.035811000	-0.014298000

Gibbs free energy = -530.625855 Hartree

Number of imaginary frequencies: 1

Product - U_c with a water molecule

angstroms

X Y Z

O	0.923064000	-2.083059000	-0.006250000
O	-2.157768000	1.295722000	-0.004271000
N	-0.647785000	-0.419878000	-0.006114000
N	1.678749000	0.070718000	0.002750000
C	0.639400000	-0.886645000	-0.003365000
C	-0.893240000	0.877232000	-0.001196000
C	1.405287000	1.399175000	0.007670000
C	0.125126000	1.862528000	0.006438000
H	-2.398489000	-1.376495000	-0.002760000
C	3.060731000	-0.423453000	0.003693000
H	2.259094000	2.063645000	0.012550000
H	3.736402000	0.428936000	0.008842000
H	3.238678000	-1.028687000	-0.884929000
H	3.234766000	-1.036369000	0.887787000
H	-0.102917000	2.917082000	0.010349000
O	-3.346663000	-1.145995000	-0.083978000
H	-3.792310000	-1.542885000	0.673392000
H	-2.764866000	0.512524000	-0.005122000

Gibbs free energy = -530.637565 Hartree

Number of imaginary frequencies: 0

Structures of purine bases optimized at the B3LYP/aug-cc-pVTZ

adenine

	angstroms		
	X	Y	Z
C	-1.560119000	-1.508555000	-0.001483000
H	-2.381811000	-2.207194000	-0.003368000
N	-0.294235000	-1.835573000	-0.000652000
C	0.363397000	-0.622652000	0.001939000

C	1.723809000	-0.271468000	0.002163000
N	2.710242000	-1.194800000	0.018841000
H	3.663142000	-0.889106000	-0.057082000
H	2.487165000	-2.169997000	-0.055796000
N	2.050417000	1.027841000	-0.002008000
C	1.069965000	1.939234000	-0.001915000
H	1.395188000	2.972784000	-0.004344000
N	-0.245618000	1.737351000	0.000230000
C	-0.542962000	0.435911000	0.001224000
C	-3.061470000	0.539295000	-0.000483000
H	-3.860167000	-0.198189000	-0.004760000
H	-3.150096000	1.171213000	-0.882628000
H	-3.154093000	1.165193000	0.885561000
N	-1.785812000	-0.151289000	-0.000161000

Gibbs free energy = -506.717100 Hartree

Number of imaginary frequencies: 0

guanine

	angstroms		
	X	Y	Z
N	-1.925788000	-0.406844000	-0.000207000
C	-2.366382000	0.902801000	0.003354000
H	-3.419478000	1.134436000	0.002744000
N	-1.397351000	1.773855000	0.007162000
C	-0.249552000	1.014711000	0.006338000
C	1.130610000	1.405829000	0.001160000
O	1.654203000	2.500254000	-0.004025000
N	1.961211000	0.235461000	-0.004656000
H	2.947166000	0.443218000	-0.063878000
C	1.536788000	-1.062460000	-0.002430000

N	2.504834000	-2.034350000	-0.062834000
H	3.404246000	-1.836239000	0.342162000
H	2.160103000	-2.964482000	0.109089000
N	0.277196000	-1.405821000	0.008511000
C	-0.560939000	-0.340957000	0.001284000
C	-2.730929000	-1.613116000	-0.005327000
H	-2.517297000	-2.211422000	-0.889806000
H	-3.780802000	-1.330947000	-0.010025000
H	-2.525844000	-2.213562000	0.879812000

Gibbs free energy = -581.980221 Hartree

Number of imaginary frequencies: 0

Structures of purine bases optimized at the B3LYP/aug-cc-pVTZ/PCM

adenine

	angstroms		
	X	Y	Z
C	-1.566246000	-1.501849000	0.000187000
H	-2.390594000	-2.196693000	0.001329000
N	-0.297560000	-1.833262000	-0.001206000
C	0.364188000	-0.621931000	-0.002197000
C	1.727741000	-0.277192000	-0.001769000
N	2.712434000	-1.197002000	-0.023230000
H	3.666889000	-0.900052000	0.080726000
H	2.496572000	-2.172053000	0.081841000
N	2.054153000	1.027108000	0.001140000
C	1.074405000	1.939450000	0.000963000

H	1.400823000	2.972967000	0.003009000
N	-0.240869000	1.739118000	-0.000211000
C	-0.544541000	0.435554000	-0.001138000
C	-3.069133000	0.536971000	0.002072000
H	-3.861146000	-0.206068000	0.002777000
H	-3.163531000	1.161027000	-0.884316000
H	-3.161300000	1.160342000	0.889185000
N	-1.786186000	-0.148172000	0.000183000

Gibbs free energy = -506.728876 Hartree

Number of imaginary frequencies: 0

guanine

	angstroms		
	X	Y	Z
N	-1.927038000	-0.404447000	0.000568000
C	-2.363505000	0.900638000	0.001608000
H	-3.415603000	1.134078000	0.002054000
N	-1.388970000	1.772871000	0.002269000
C	-0.241417000	1.005909000	0.001063000
C	1.132526000	1.383285000	0.000185000
O	1.638717000	2.503949000	0.000429000
N	1.964073000	0.240837000	-0.001904000
H	2.954048000	0.440662000	-0.019793000
C	1.544940000	-1.064139000	-0.000933000
N	2.503180000	-2.022827000	-0.051465000
H	3.448236000	-1.808227000	0.217210000
H	2.197176000	-2.965366000	0.121865000
N	0.276132000	-1.406006000	0.003800000
C	-0.561772000	-0.348357000	-0.000004000
C	-2.746166000	-1.606740000	-0.000902000

H	-2.542066000	-2.200721000	-0.889279000
H	-3.791668000	-1.313806000	0.001483000
H	-2.539131000	-2.204781000	0.884044000

Gibbs free energy = -582.005158 Hartree

Number of imaginary frequencies: 0

Structures of pyrimidine-purine complexes optimized at the B3LYP/aug-cc-pVTZ

AMu

	angstroms		
	X	Y	Z
O	2.062700000	-2.354789000	0.000021000
O	2.842507000	3.376271000	0.000395000
N	2.102580000	-0.077877000	-0.000619000
N	2.028921000	2.206923000	-0.000224000
C	4.794352000	-0.131823000	0.000091000
C	2.693448000	-1.311662000	-0.000542000
C	4.204256000	1.076524000	0.000275000
C	2.764157000	1.144873000	-0.000188000
H	5.869790000	-0.236934000	0.000421000
H	4.783322000	1.981778000	0.000639000
H	2.192587000	4.083812000	0.000022000
C	4.758871000	-2.608073000	0.000751000
H	4.484524000	-3.177749000	0.886905000
H	5.833161000	-2.444411000	-0.008193000
H	4.470945000	-3.185698000	-0.875703000

N	4.092011000	-1.310643000	-0.000770000
H	1.065371000	-0.086775000	-0.000473000
N	-4.792392000	-0.543910000	0.000406000
C	-5.106646000	0.796220000	0.000263000
H	-6.134012000	1.124775000	0.000393000
N	-4.063576000	1.584117000	-0.000120000
C	-2.990898000	0.717077000	-0.000111000
C	-1.599054000	0.925188000	-0.000358000
N	-1.041609000	2.146347000	-0.000727000
H	-0.029325000	2.249082000	-0.000687000
H	-1.639984000	2.952006000	-0.000598000
N	-0.806018000	-0.162734000	-0.000271000
C	-1.357451000	-1.386970000	0.000039000
H	-0.649642000	-2.207171000	0.000097000
N	-2.647949000	-1.699011000	0.000275000
C	-3.420001000	-0.607710000	0.000193000
C	-5.703804000	-1.672362000	0.000613000
H	-5.544545000	-2.288284000	0.884303000
H	-6.724942000	-1.298962000	0.001657000
H	-5.546067000	-2.287499000	-0.883906000

Gibbs free energy = -1016.230580 Hartree

Number of imaginary frequencies: 0

AMu-m

	angstroms		
	X	Y	Z
O	1.860193000	-2.026878000	0.121600000
O	1.488700000	2.645980000	0.729244000
N	2.301114000	0.211659000	0.207524000
N	2.874229000	2.517182000	0.401963000

C	4.894911000	-0.305896000	-0.377087000
C	2.655986000	-1.110992000	0.024236000
C	4.546791000	0.971466000	-0.172519000
C	3.178411000	1.286626000	0.163384000
H	5.906074000	-0.592893000	-0.623846000
H	5.262396000	1.772702000	-0.241762000
H	1.455621000	3.535182000	1.094508000
C	4.394489000	-2.732285000	-0.482091000
H	4.225599000	-3.315831000	0.421562000
H	5.451414000	-2.751463000	-0.733861000
H	3.820007000	-3.183469000	-1.288940000
N	3.991779000	-1.344588000	-0.281385000
H	1.294750000	0.355912000	0.358928000
N	-4.677506000	-0.693133000	-0.029451000
C	-5.081592000	0.526057000	-0.524591000
H	-6.119964000	0.718341000	-0.744445000
N	-4.106258000	1.380836000	-0.688201000
C	-2.984922000	0.688865000	-0.278022000
C	-1.625699000	1.039249000	-0.197971000
N	-1.165398000	2.246613000	-0.579960000
H	-0.208122000	2.491579000	-0.370742000
H	-1.822795000	2.953767000	-0.853905000
N	-0.760063000	0.119762000	0.261362000
C	-1.220293000	-1.090945000	0.617413000
H	-0.463230000	-1.788034000	0.950442000
N	-2.474834000	-1.529835000	0.590804000
C	-3.316227000	-0.598668000	0.135146000
C	-5.498867000	-1.851799000	0.264182000
H	-5.426514000	-2.111199000	1.319321000
H	-6.533002000	-1.620175000	0.020931000

H	-5.172758000	-2.706773000	-0.326027000

Gibbs free energy = -1016.228204 Hartree

Number of imaginary frequencies: 0

AU

	angstroms		
	X	Y	Z
C	-4.976038000	0.751539000	0.000325000
H	-6.013174000	1.047891000	0.000466000
N	-3.957706000	1.570796000	0.000105000
C	-2.858688000	0.737798000	0.000008000
C	-1.473224000	0.992585000	-0.000149000
N	-0.957878000	2.228978000	-0.000234000
H	0.050047000	2.373339000	-0.000320000
H	-1.583086000	3.014239000	-0.000130000
N	-0.647053000	-0.073588000	-0.000243000
C	-1.161927000	-1.313407000	-0.000195000
H	-0.430835000	-2.113268000	-0.000283000
N	-2.440810000	-1.666731000	-0.000059000
C	-3.246677000	-0.599178000	0.000043000
C	-5.496205000	-1.734422000	0.000255000
H	-5.319173000	-2.344887000	-0.883925000
H	-6.528408000	-1.392651000	0.000317000
H	-5.319058000	-2.344859000	0.884432000
N	-4.620160000	-0.578486000	0.000183000

O	2.461121000	-2.078497000	-0.000160000
O	1.955273000	2.453687000	-0.000068000
N	4.348536000	-0.793728000	-0.000018000
N	2.228690000	0.193955000	-0.000297000
C	5.168756000	-2.002127000	0.000278000
C	2.960998000	-0.969741000	-0.000349000
C	4.900632000	0.457373000	0.000250000
C	4.164249000	1.585236000	0.000202000
C	2.720525000	1.492359000	-0.000056000
H	4.956250000	-2.603303000	0.882166000
H	5.981140000	0.482677000	0.000504000
H	1.192779000	0.077871000	-0.000380000
H	4.622097000	2.559992000	0.000396000
H	4.955098000	-2.604361000	-0.880588000
H	6.215443000	-1.710661000	-0.000571000

Gibbs free energy = -960.940626 Hartree

Number of imaginary frequencies: 0

AC_u

	angstroms		
	X	Y	Z
O	-2.379561000	-2.051801000	-0.000074000
N	-4.320599000	-0.853383000	0.000021000
N	-2.241815000	0.223994000	-0.000027000
N	-1.985647000	2.509480000	0.000049000
C	-5.086902000	-2.095002000	0.000026000
C	-4.926112000	0.375614000	0.000088000
C	-2.925831000	-0.963445000	-0.000059000
C	-4.235307000	1.529344000	0.000094000
C	-2.787072000	1.500695000	0.000041000

H	-4.850329000	-2.688357000	-0.881285000
H	-6.006659000	0.358528000	0.000138000
H	-4.749129000	2.477090000	0.000147000
H	-2.504661000	3.380675000	0.000093000
H	-6.145357000	-1.848600000	0.000084000
H	-4.850245000	-2.688406000	0.881282000
H	-1.205649000	0.138331000	-0.000071000
C	4.988492000	0.730143000	0.000225000
H	6.029488000	1.012632000	0.000348000
N	3.981067000	1.562923000	-0.000039000
C	2.870692000	0.744457000	-0.000034000
C	1.488103000	1.017612000	-0.000127000
N	0.986759000	2.259040000	-0.000234000
H	-0.025653000	2.415660000	-0.000147000
H	1.624644000	3.034221000	-0.000083000
N	0.648425000	-0.038232000	-0.000171000
C	1.146658000	-1.284177000	-0.000129000
H	0.404357000	-2.073553000	-0.000164000
N	2.421694000	-1.654644000	-0.000044000
C	3.240856000	-0.597359000	0.000001000
C	5.474939000	-1.762454000	0.000173000
H	6.511819000	-1.435007000	0.000682000
H	5.289904000	-2.370496000	-0.884093000
H	5.289175000	-2.370930000	0.883983000
N	4.614927000	-0.594884000	0.000144000

Gibbs free energy = -941.032039 Hartree

Number of imaginary frequencies: 0

GM_c

angstroms

	X	Y	Z
O	-2.270239000	-2.263411000	-0.057919000
O	-2.482256000	3.511928000	0.075352000
N	-2.102909000	0.008203000	-0.122959000
N	-1.930177000	2.264946000	-0.239110000
C	-4.819309000	0.133046000	0.141449000
C	-2.795830000	-1.148171000	-0.037199000
C	-4.134263000	1.297573000	0.055705000
C	-2.718757000	1.189573000	-0.092643000
H	-5.893901000	0.109442000	0.254842000
H	-4.628367000	2.251141000	0.105157000
H	-2.342306000	4.049372000	-0.714108000
C	-4.943192000	-2.325798000	0.188269000
H	-4.766858000	-2.934682000	-0.695925000
H	-6.002907000	-2.099621000	0.274399000
H	-4.614798000	-2.891033000	1.058178000
N	-4.201054000	-1.072457000	0.091338000
H	-0.901144000	2.190334000	-0.097800000
C	5.000677000	1.091849000	0.123168000
H	6.004471000	1.483191000	0.171988000
N	3.909790000	1.801389000	0.115921000
C	2.893331000	0.871105000	0.045042000
C	1.477941000	1.021584000	0.006269000
O	0.805478000	2.059333000	0.029297000
N	0.832029000	-0.222489000	-0.067763000
H	-0.195447000	-0.182551000	-0.103879000
C	1.446254000	-1.446385000	-0.098165000
N	0.641283000	-2.523977000	-0.181179000
H	-0.374215000	-2.453634000	-0.145560000
H	1.082488000	-3.424238000	-0.158740000

N	2.756437000	-1.595501000	-0.059021000
C	3.413175000	-0.423070000	0.009547000
C	5.753754000	-1.337277000	0.050254000
H	6.747120000	-0.897467000	0.097400000
H	5.666827000	-1.928978000	-0.860142000
H	5.612831000	-1.996087000	0.906217000
N	4.771695000	-0.271850000	0.060480000

Gibbs free energy = -1091.504924 Hartree

Number of imaginary frequencies: 0

GC

angstroms

	X	Y	Z
N	-4.640717000	-0.332155000	0.000582000
C	-4.949190000	1.017261000	0.000696000
H	-5.974809000	1.350844000	0.001033000
N	-3.900654000	1.788147000	0.000508000
C	-2.830936000	0.916656000	-0.000017000
C	-1.424968000	1.149321000	-0.000563000
O	-0.813724000	2.222273000	-0.000708000
N	-0.707013000	-0.059004000	-0.000857000
H	0.317240000	0.038450000	-0.001253000
C	-1.250103000	-1.316275000	-0.000651000
N	-0.384555000	-2.348987000	-0.000976000
H	0.625702000	-2.221833000	-0.001411000
H	-0.775565000	-3.272257000	-0.000867000
N	-2.550354000	-1.539839000	-0.000154000
C	-3.274586000	-0.405573000	0.000137000
C	-5.559246000	-1.452560000	0.000969000
H	-6.577243000	-1.070136000	0.000422000
H	-5.409364000	-2.070404000	0.885558000

H	-5.408778000	-2.071378000	-0.882828000
O	2.510794000	-1.943923000	-0.000706000
N	4.386093000	-0.662412000	0.000496000
N	2.223547000	0.316473000	-0.000509000
N	1.957027000	2.586723000	-0.000368000
C	5.193690000	-1.878227000	0.000753000
C	4.947118000	0.572299000	0.000936000
C	2.980248000	-0.804235000	-0.000254000
C	4.195892000	1.696173000	0.000678000
C	2.772255000	1.530323000	-0.000081000
H	4.970520000	-2.477418000	0.881171000
H	6.027433000	0.603401000	0.001497000
H	4.655895000	2.671434000	0.001033000
H	0.932121000	2.462579000	-0.000925000
H	2.339395000	3.514167000	-0.000066000
H	6.244784000	-1.600668000	0.001352000
H	4.971459000	-2.477170000	-0.880073000

Gibbs free energy = -1016.318867 Hartree

Number of imaginary frequencies: 0

GU_c

	angstroms		
	X	Y	Z
O	2.531020000	-1.954414000	0.001073000
O	1.858565000	2.553365000	-0.001292000
N	4.355675000	-0.594244000	0.000506000
N	2.161238000	0.294662000	0.000315000
C	5.217496000	-1.773578000	0.001051000
C	2.963339000	-0.801944000	0.000567000
C	4.862557000	0.665642000	-0.000163000
C	4.062166000	1.756806000	-0.000766000

C	2.655426000	1.519942000	-0.000580000
H	5.020244000	-2.380503000	0.882205000
H	5.940410000	0.741431000	-0.000176000
H	4.457021000	2.758607000	-0.001307000
H	5.020159000	-2.381364000	-0.879489000
H	6.254877000	-1.449725000	0.000843000
H	0.864159000	2.307208000	-0.000707000
N	-4.602351000	-0.271972000	0.000241000
C	-4.860203000	1.088363000	0.000848000
H	-5.872743000	1.459675000	0.001239000
N	-3.784826000	1.820544000	0.000973000
C	-2.748252000	0.910508000	0.000379000
C	-1.337708000	1.082811000	0.000252000
O	-0.682695000	2.138425000	0.000567000
N	-0.667576000	-0.142246000	-0.000384000
H	0.361430000	-0.069054000	-0.000240000
C	-1.252042000	-1.378962000	-0.000894000
N	-0.415454000	-2.436365000	-0.001977000
H	0.595230000	-2.328632000	-0.000089000
H	-0.826419000	-3.350836000	-0.000396000
N	-2.558710000	-1.556583000	-0.000784000
C	-3.240930000	-0.396645000	-0.000127000
C	-5.563092000	-1.357231000	-0.000010000
H	-6.565574000	-0.936178000	0.000184000
H	-5.436158000	-1.980506000	0.884170000
H	-5.436265000	-1.979963000	-0.884584000

Gibbs free energy = -1036.208193 Hartree

Number of imaginary frequencies: 0

GM_u-wobble

	angstroms		
	X	Y	Z
N	4.867345000	-0.574248000	0.022410000
C	4.595920000	-1.930361000	0.032810000
H	5.394053000	-2.655384000	0.050003000
N	3.322974000	-2.203826000	0.019071000
C	2.704828000	-0.972615000	-0.002217000
C	1.322638000	-0.610753000	-0.019510000
O	0.322225000	-1.324683000	-0.016030000
N	1.170323000	0.791083000	-0.040933000
H	0.196955000	1.119671000	-0.047228000
C	2.180673000	1.704139000	-0.038846000
N	1.809989000	3.016630000	-0.105402000
H	0.863813000	3.267965000	0.124258000
H	2.529055000	3.687503000	0.099332000
N	3.452826000	1.386252000	-0.012725000
C	3.651548000	0.050079000	-0.000197000
C	6.165526000	0.070696000	0.033144000
H	6.286087000	0.700547000	-0.847090000
H	6.936107000	-0.696336000	0.032022000
H	6.278652000	0.690061000	0.922014000
O	-1.487676000	1.707363000	0.021256000
O	-4.357823000	-3.316885000	-0.022720000
N	-2.376887000	-0.393934000	0.001767000
N	-3.177149000	-2.533490000	-0.018245000
C	-4.859014000	0.670435000	0.018933000
C	-2.471211000	0.957939000	0.015579000
C	-4.769203000	-0.666311000	0.005647000
C	-3.462312000	-1.282690000	-0.004529000
H	-5.809630000	1.182777000	0.026560000

H	-5.646963000	-1.287252000	0.001984000
H	-4.000712000	-4.209374000	-0.033417000
C	-3.878029000	2.947575000	0.037369000
H	-3.418015000	3.367183000	0.930614000
H	-4.935122000	3.198209000	0.026463000
H	-3.395180000	3.384638000	-0.834848000
N	-3.749364000	1.494769000	0.023404000
H	-1.421489000	-0.788016000	-0.005241000

Gibbs free energy = -1091.50012 Hartree

Number of imaginary frequencies: 0

GU-wobble

angstroms

	X	Y	Z
N	4.685093000	-0.366337000	0.016871000
C	4.520563000	-1.739653000	0.026898000
H	5.372965000	-2.400064000	0.040542000
N	3.272997000	-2.111845000	0.017443000
C	2.560542000	-0.932980000	-0.000323000
C	1.154512000	-0.681111000	-0.013093000
O	0.213426000	-1.471136000	-0.008189000
N	0.892472000	0.705568000	-0.032124000
H	-0.102286000	0.953913000	-0.038978000
C	1.828624000	1.695332000	-0.032528000
N	1.357444000	2.976020000	-0.097855000
H	0.399018000	3.155106000	0.147746000
H	2.025654000	3.698245000	0.105458000
N	3.121013000	1.477271000	-0.010931000
C	3.424303000	0.160619000	-0.000710000

C	5.929119000	0.378192000	0.023464000
H	5.996455000	1.016384000	-0.856375000
H	6.757114000	-0.326377000	0.018143000
H	5.997038000	1.003269000	0.912875000
O	-1.860310000	1.373681000	0.027410000
O	-3.232618000	-2.988690000	-0.028471000
N	-4.090933000	0.946999000	0.019968000
N	-2.553615000	-0.808272000	-0.000717000
C	-4.355571000	2.383356000	0.035745000
C	-2.767123000	0.537467000	0.016089000
C	-5.113266000	0.026556000	0.007916000
C	-4.898377000	-1.297813000	-0.008310000
C	-3.539653000	-1.815946000	-0.014062000
H	-3.931714000	2.839596000	0.928278000
H	-6.107646000	0.448800000	0.012295000
H	-1.564194000	-1.113414000	-0.004180000
H	-5.713331000	-2.002189000	-0.017659000
H	-3.915228000	2.861220000	-0.837095000
H	-5.431142000	2.534711000	0.027844000

Gibbs free energy = -1036.20691 Hartree

Number of imaginary frequencies: 0

Structures of pyrimidine-purine complexes optimized at the B3LYP/aug-cc-pVTZ/PCM

AMu

	angstroms		
	X	Y	Z
O	-2.132672000	-2.367315000	0.000388000
O	-2.764288000	3.383527000	-0.000569000
N	-2.132739000	-0.093836000	0.000047000
N	-1.995165000	2.184353000	-0.000406000
C	-4.820415000	-0.087845000	-0.000073000
C	-2.755080000	-1.310782000	0.000284000
C	-4.203271000	1.108413000	-0.000218000
C	-2.765130000	1.145763000	-0.000211000
H	-5.896871000	-0.169830000	-0.000116000
H	-4.769360000	2.021919000	-0.000388000
H	-2.088233000	4.068923000	-0.000792000
C	-4.853627000	-2.560497000	0.000271000
H	-4.594355000	-3.138355000	-0.884254000
H	-5.920329000	-2.360688000	-0.000186000
H	-4.595008000	-3.137812000	0.885348000
N	-4.143957000	-1.281727000	0.000120000

H	-1.099582000	-0.120248000	0.000150000
N	4.815972000	-0.550268000	-0.000022000
C	5.127695000	0.785556000	0.000016000
H	6.154531000	1.114122000	-0.000010000
N	4.079710000	1.573523000	0.000133000
C	3.007799000	0.704559000	0.000065000
C	1.614674000	0.911383000	0.000071000
N	1.049304000	2.126238000	0.000138000
H	0.035347000	2.221734000	0.000106000
H	1.630291000	2.945282000	0.000118000
N	0.822014000	-0.181720000	0.000054000
C	1.380059000	-1.400375000	0.000040000
H	0.677612000	-2.225472000	0.000031000
N	2.671038000	-1.711620000	0.000029000
C	3.446165000	-0.617998000	0.000046000
C	5.743361000	-1.670693000	-0.000099000
H	5.593860000	-2.283232000	0.886745000
H	5.593647000	-2.283277000	-0.886875000
H	6.757509000	-1.282264000	-0.000232000

Gibbs free energy = -1016.251441 Hartree

Number of imaginary frequencies: 0

AMu-m

	angstroms		
	X	Y	Z
O	1.862107000	-2.015280000	-0.066182000
O	1.454926000	2.602794000	0.738714000
N	2.316625000	0.204572000	0.182278000
N	2.858149000	2.500291000	0.493419000
C	4.932347000	-0.293797000	-0.283732000

C	2.680757000	-1.105129000	-0.060164000
C	4.575661000	0.974975000	-0.037840000
C	3.192556000	1.280466000	0.228830000
H	5.954871000	-0.573912000	-0.483768000
H	5.302519000	1.769354000	-0.028423000
H	1.365072000	3.508921000	1.052898000
C	4.446224000	-2.706490000	-0.555361000
H	4.199433000	-3.346628000	0.289000000
H	5.520695000	-2.710221000	-0.707401000
H	3.954671000	-3.094535000	-1.445137000
N	4.022744000	-1.331277000	-0.294597000
H	1.308555000	0.345363000	0.310554000
N	-4.695360000	-0.685547000	0.004107000
C	-5.088600000	0.486059000	-0.591078000
H	-6.123022000	0.660522000	-0.839858000
N	-4.105406000	1.325654000	-0.809395000
C	-2.991057000	0.667593000	-0.329033000
C	-1.631506000	1.021461000	-0.257201000
N	-1.149510000	2.188071000	-0.725985000
H	-0.205245000	2.449308000	-0.477680000
H	-1.791647000	2.905951000	-1.011997000
N	-0.775429000	0.133820000	0.284976000
C	-1.247620000	-1.038122000	0.731652000
H	-0.501273000	-1.707963000	1.138946000
N	-2.502815000	-1.475957000	0.723462000
C	-3.338918000	-0.582264000	0.179973000
C	-5.537020000	-1.811955000	0.375892000
H	-5.481581000	-1.985031000	1.448746000
H	-6.563163000	-1.585274000	0.102221000
H	-5.216083000	-2.710136000	-0.147848000

Gibbs free energy = -1016.250242 Hartree

Number of imaginary frequencies: 0

AU

	angstroms		
	X	Y	Z
C	-4.994267000	0.754524000	0.000302000
H	-6.030416000	1.052589000	0.000488000
N	-3.970026000	1.573038000	0.000197000
C	-2.872896000	0.736346000	-0.000064000
C	-1.486458000	0.984821000	-0.000383000
N	-0.957283000	2.215612000	-0.000561000
H	0.052708000	2.343194000	-0.000778000
H	-1.563344000	3.016272000	-0.000429000
N	-0.662561000	-0.085221000	-0.000510000
C	-1.184137000	-1.319946000	-0.000347000
H	-0.458477000	-2.124849000	-0.000462000
N	-2.464834000	-1.669036000	-0.000048000
C	-3.271945000	-0.598646000	0.000082000
C	-5.536683000	-1.719113000	0.000663000
H	-5.369060000	-2.326957000	-0.886194000
H	-6.561918000	-1.361024000	0.000868000
H	-5.368667000	-2.326850000	0.887520000
N	-4.643029000	-0.571553000	0.000387000
O	2.476850000	-2.075651000	-0.000978000

O	1.964393000	2.449130000	0.000058000
N	4.369740000	-0.792773000	0.000182000
N	2.254616000	0.193409000	-0.000378000
C	5.204898000	-1.995789000	0.000130000
C	2.992163000	-0.966682000	-0.000315000
C	4.919463000	0.457373000	0.000708000
C	4.179938000	1.586488000	0.000725000
C	2.744702000	1.490088000	0.000127000
H	5.000124000	-2.594102000	0.884884000
H	5.998838000	0.484863000	0.001102000
H	1.224103000	0.074067000	-0.000891000
H	4.644427000	2.558202000	0.001142000
H	5.000690000	-2.593638000	-0.885068000
H	6.246018000	-1.690705000	0.000542000

Gibbs free energy = -960.961837 Hartree

Number of imaginary frequencies: 0

AC_u

angstroms

	X	Y	Z
O	-2.438502000	-2.064656000	-0.000301000
N	-4.364439000	-0.835906000	0.000211000
N	-2.274847000	0.205348000	-0.000083000
N	-1.969603000	2.484930000	-0.000101000
C	-5.166862000	-2.058661000	0.000204000
C	-4.949561000	0.405131000	0.000340000
C	-2.983729000	-0.966661000	0.000099000
C	-4.239354000	1.547850000	0.000288000

C	-2.796406000	1.493653000	0.000028000
H	-4.950687000	-2.653585000	-0.884598000
H	-6.029213000	0.404844000	0.000470000
H	-4.738180000	2.503404000	0.000384000
H	-2.471299000	3.366741000	0.000004000
H	-6.215740000	-1.780344000	0.000300000
H	-4.950548000	-2.653676000	0.884911000
H	-1.246482000	0.103551000	-0.000362000
C	5.012541000	0.740827000	0.000119000
H	6.050959000	1.030874000	0.000187000
N	3.994383000	1.567070000	-0.000059000
C	2.890426000	0.738857000	-0.000058000
C	1.505081000	0.999675000	-0.000127000
N	0.985276000	2.232900000	-0.000162000
H	-0.031738000	2.373236000	-0.000328000
H	1.602746000	3.025212000	-0.000216000
N	0.672850000	-0.065545000	-0.000157000
C	1.185652000	-1.303151000	-0.000128000
H	0.453869000	-2.102617000	-0.000163000
N	2.464190000	-1.663429000	-0.000072000
C	3.279217000	-0.598699000	-0.000035000
C	5.535357000	-1.736781000	0.000029000
H	5.362884000	-2.343455000	0.886813000
H	6.563467000	-1.386945000	0.000057000
H	5.362929000	-2.343448000	-0.886768000
N	4.650887000	-0.582335000	0.000017000

Gibbs free energy = -941.052157 Hartree

Number of imaginary frequencies: 0

GM_c

	angstroms		
	X	Y	Z
O	-2.240688000	-2.258244000	-0.048979000
O	-2.514043000	3.517535000	0.038706000
N	-2.111794000	0.013110000	-0.140748000
N	-1.973925000	2.273933000	-0.307099000
C	-4.824802000	0.101351000	0.161245000
C	-2.794302000	-1.148820000	-0.029947000
C	-4.155569000	1.275431000	0.047691000
C	-2.748728000	1.184411000	-0.118090000
H	-5.896365000	0.063441000	0.287698000
H	-4.668272000	2.219826000	0.080162000
H	-2.598345000	3.999461000	-0.794831000
C	-4.924196000	-2.352377000	0.243323000
H	-4.762426000	-2.965495000	-0.639971000
H	-5.981351000	-2.127838000	0.343578000
H	-4.581649000	-2.901204000	1.117400000
N	-4.188370000	-1.093312000	0.120577000
H	-0.959702000	2.206527000	-0.142641000
C	5.018760000	1.075296000	0.157695000
H	6.024616000	1.458152000	0.216222000
N	3.927633000	1.794325000	0.169176000
C	2.905964000	0.868303000	0.074384000
C	1.495379000	1.029932000	0.039079000
O	0.839927000	2.084523000	0.089587000
N	0.834342000	-0.196753000	-0.066153000
H	-0.195634000	-0.149190000	-0.104818000
C	1.440257000	-1.426585000	-0.128101000
N	0.628501000	-2.491902000	-0.243085000
H	-0.387228000	-2.410134000	-0.178253000

H	1.053595000	-3.401598000	-0.225090000
N	2.752333000	-1.585770000	-0.092653000
C	3.423202000	-0.424337000	0.005667000
C	5.770932000	-1.343238000	0.023696000
H	6.760806000	-0.899244000	0.075308000
H	5.680735000	-1.910903000	-0.900387000
H	5.635161000	-2.015159000	0.869003000
N	4.781726000	-0.278397000	0.060307000

Gibbs free energy = -1091.531703 Hartree

Number of imaginary frequencies: 0

GC

	angstroms		
	X	Y	Z
N	4.647134000	-0.348027000	0.000183000
C	4.968886000	0.991746000	-0.000382000
H	5.997539000	1.313999000	-0.000429000
N	3.923979000	1.776531000	-0.000849000
C	2.845274000	0.911955000	-0.000428000
C	1.445732000	1.159826000	-0.000507000
O	0.857804000	2.252977000	-0.001004000
N	0.707893000	-0.028602000	-0.000140000
H	-0.319163000	0.080015000	0.000051000
C	1.237272000	-1.295134000	0.000253000
N	0.362141000	-2.314013000	0.000551000
H	-0.649532000	-2.169999000	0.000499000
H	0.733819000	-3.246738000	0.001053000
N	2.538502000	-1.532976000	0.000373000
C	3.280920000	-0.411774000	0.000007000
C	5.567690000	-1.473147000	0.000815000
H	5.416065000	-2.085597000	0.887629000

H	6.584069000	-1.090289000	0.000562000
H	5.416003000	-2.086603000	-0.885289000
O	-2.477583000	-1.934897000	-0.000932000
N	-4.375999000	-0.685752000	-0.000176000
N	-2.233524000	0.328702000	-0.000093000
N	-2.011890000	2.608037000	0.000924000
C	-5.175462000	-1.910763000	-0.000595000
C	-4.960983000	0.538754000	0.000480000
C	-2.979511000	-0.799476000	-0.000428000
C	-4.228879000	1.676099000	0.000887000
C	-2.808949000	1.534603000	0.000567000
H	-4.952194000	-2.503523000	-0.884697000
H	-6.040619000	0.549084000	0.000661000
H	-4.704402000	2.643362000	0.001406000
H	-0.995896000	2.500279000	0.000606000
H	-2.408724000	3.530514000	0.001413000
H	-6.226200000	-1.638376000	-0.000454000
H	-4.952128000	-2.504185000	0.883048000

Gibbs free energy = -1016.345648 Hartree

Number of imaginary frequencies: 0

GU_c

	angstroms		
	X	Y	Z
O	-2.513278000	-1.948663000	-0.000693000
O	-1.875857000	2.562631000	0.000187000
N	-4.353641000	-0.604227000	0.000199000
N	-2.169760000	0.302414000	-0.000267000
C	-5.217106000	-1.787123000	0.000169000
C	-2.967591000	-0.798589000	-0.000270000
C	-4.872292000	0.647619000	0.000719000

C	-4.079311000	1.748752000	0.000765000
C	-2.681595000	1.519470000	0.000212000
H	-5.021218000	-2.388679000	-0.884060000
H	-5.949626000	0.714191000	0.001096000
H	-4.488975000	2.744453000	0.001176000
H	-5.020963000	-2.388933000	0.884172000
H	-6.252129000	-1.461270000	0.000364000
H	-0.899450000	2.302594000	-0.000304000
N	4.607962000	-0.280985000	0.000401000
C	4.872933000	1.071308000	0.000285000
H	5.887290000	1.435731000	0.000491000
N	3.797359000	1.812789000	-0.000095000
C	2.756033000	0.904270000	-0.000182000
C	1.351139000	1.084017000	-0.000617000
O	0.710356000	2.157062000	-0.000974000
N	0.666948000	-0.125126000	-0.000680000
H	-0.364723000	-0.045015000	-0.000722000
C	1.242935000	-1.368722000	-0.000216000
N	0.400588000	-2.415903000	-0.000127000
H	-0.610412000	-2.294538000	-0.000369000
H	0.794042000	-3.339474000	-0.000042000
N	2.552029000	-1.553891000	0.000134000
C	3.246724000	-0.402926000	0.000107000
C	5.576359000	-1.365712000	0.000775000
H	6.575013000	-0.938859000	0.000812000
H	5.450927000	-1.984255000	-0.885706000
H	5.450692000	-1.983867000	0.887494000

Gibbs free energy = -1036.232437 Hartree

Number of imaginary frequencies: 0

GM$_u$-wobble

	angstroms		
	X	Y	Z
N	4.870503000	-0.568742000	0.017558000
C	4.600353000	-1.918888000	0.000842000
H	5.398223000	-2.643531000	0.005765000
N	3.322377000	-2.193131000	-0.020810000
C	2.706713000	-0.957039000	-0.018655000
C	1.332499000	-0.594527000	-0.031828000
O	0.336018000	-1.333169000	-0.047761000
N	1.165687000	0.794749000	-0.023562000
H	0.190895000	1.123710000	-0.034589000
C	2.176085000	1.715639000	-0.002229000
N	1.808174000	3.020075000	-0.042889000
H	0.860405000	3.279530000	0.172032000
H	2.521397000	3.700075000	0.156741000
N	3.452110000	1.392451000	0.017167000
C	3.657956000	0.061068000	0.005021000
C	6.178708000	0.066460000	0.044125000
H	6.308488000	0.698986000	-0.831706000
H	6.940227000	-0.707778000	0.042431000
H	6.285695000	0.672853000	0.941318000
O	-1.503476000	1.709406000	-0.026559000
O	-4.319732000	-3.344054000	0.024901000
N	-2.386260000	-0.387886000	-0.010277000
N	-3.144964000	-2.538958000	0.006890000
C	-4.864962000	0.653767000	0.020246000
C	-2.492811000	0.968048000	-0.011653000
C	-4.763708000	-0.686317000	0.022238000
C	-3.454647000	-1.287622000	0.006866000

H	-5.821519000	1.153359000	0.031230000
H	-5.641817000	-1.306066000	0.035065000
H	-3.958020000	-4.235964000	0.022550000
C	-3.922541000	2.944782000	0.003357000
H	-3.448213000	3.376136000	0.881981000
H	-4.982474000	3.176478000	0.016012000
H	-3.470044000	3.372731000	-0.888331000
N	-3.770368000	1.488965000	0.004262000
H	-1.428391000	-0.762889000	-0.023521000

Gibbs free energy = -1091.530117 Hartree

Number of imaginary frequencies: 0

GU-wobble

	angstroms		
	X	Y	Z
N	4.681738000	-0.357417000	0.046519000
C	4.521320000	-1.724554000	0.004798000
H	5.374466000	-2.383073000	0.018452000
N	3.270357000	-2.099903000	-0.050196000
C	2.557625000	-0.917015000	-0.045838000
C	1.159734000	-0.665519000	-0.082406000
O	0.226713000	-1.482063000	-0.127274000
N	0.881082000	0.705840000	-0.060085000
H	-0.115155000	0.955038000	-0.087143000
C	1.813913000	1.704720000	-0.004402000
N	1.343957000	2.975856000	-0.035679000
H	0.374457000	3.156390000	0.161863000
H	1.996373000	3.707651000	0.187510000
N	3.110750000	1.483666000	0.038737000

C	3.423273000	0.173483000	0.013321000
C	5.933506000	0.380374000	0.113517000
H	6.052192000	1.005532000	-0.769307000
H	6.753227000	-0.330257000	0.161995000
H	5.951726000	1.009159000	1.001253000
O	-1.878941000	1.384024000	-0.091539000
O	-3.152452000	-2.989333000	0.048236000
N	-4.110162000	0.930569000	0.011685000
N	-2.547465000	-0.797173000	-0.023584000
C	-4.412384000	2.364792000	-0.001761000
C	-2.785517000	0.549908000	-0.037860000
C	-5.105159000	-0.008637000	0.076463000
C	-4.862384000	-1.333962000	0.093311000
C	-3.502990000	-1.812624000	0.040682000
H	-3.970527000	2.849339000	0.865624000
H	-6.108076000	0.388737000	0.113546000
H	-1.555730000	-1.077625000	-0.063113000
H	-5.668319000	-2.046746000	0.144551000
H	-4.014738000	2.821814000	-0.904414000
H	-5.489860000	2.487149000	0.023061000

Gibbs free energy = -1036.239138 Hartree

Number of imaginary frequencies: 0
